# Pediatric Refugee Health Care Delivery in the Community Setting: An Educational Workshop for Multidisciplinary Family-Centered Care During Resettlement

**DOI:** 10.15766/mep_2374-8265.10988

**Published:** 2020-11-03

**Authors:** Umbereen S. Nehal, Satoko Kanahara, Mihoko Tanabe, Grace Hayner, Brett D. Nelson

**Affiliations:** 1 Chief Medical Officer and Vice President of Medical Affairs, Community Healthcare Network; Assistant Professor, Department of Pediatrics, University of Massachusetts Medical School; 2 Medical Director of South Bronx Center, Community Healthcare Network; 3 Medical Student, Philadelphia College of Osteopathic Medicine; 4 Advanced Practice Nurse, Community Healthcare Network; 5 Associate Professor, Department of Pediatrics, Harvard Medical School

**Keywords:** Refugees, Trauma-Informed Care, Family-Centered Care, Shared Decision Making, Cultural Respect, Community, Ethics, Community-Based Medicine, Cultural Competence, Global Health, Pediatrics, Diversity, Inclusion, Health Equity

## Abstract

**Introduction:**

With 70.8 million people displaced worldwide, there is an increasing need for medical professionals to provide medical care to refugees. Insufficient training on refugee health poses a barrier to effective care delivery.

**Methods:**

This workshop addressed common challenges in providing family-centered pediatric refugee care in community settings as well as barriers related to policy changes. Presentations covered prearrival experiences, medical screening, and trauma-based care. In small groups, participants discussed cases that featured medical, behavioral health, social, and cultural factors impacting the provision of family-centered pediatric care that was culturally respectful and included shared decision-making. After the breakout session, each small group informed the larger group of topics discussed. Facilitators identified themes and reinforced key learning points. At the workshop's conclusion, participants were guided to create their own personalized action plan.

**Results:**

This workshop was presented at two international conferences to more than 47 participants, including clinicians, nurse practitioners, pediatric residents, and medical students. Evaluations were completed by 34 individuals. Participants' overall comfort level with taking care of refugee patients increased from 3.3 to 4.0 (on a 5-point scale, *p* = .24) during the 3-hour version of the workshop and from 3.8 to 4.0 (*p* = .43) in the 1-hour version of the workshop. Mean overall ratings of the 3- and 1-hour workshop versions on conference-administered evaluations were 4.8 and 4.2, respectively, on a 5-point scale.

**Discussions:**

This workshop was well received and equipped participants with knowledge, tools, and strategies regarding pediatric refugee health in a community setting.

## Educational Objectives

By the end of this activity, participants will be able to:

1. Identify key aspects of physical, behavioral, and social health that affect pediatric refugee patients.
2. Decide what diseases to screen for and what treatments to give to refugee patients prior to arrival.
3. Plan whole-person care for pediatric refugee patients that is trauma informed and culturally sensitive.
4. Improve effectiveness as an advocate for funding and resources needed to deliver care in their own setting.

## Introduction

The world is currently experiencing the highest levels of displacement in history. Of the 70.8 million individuals currently displaced, about half are under the age of 18. Numbers displaced are increasing in part due to deterioration or conflict in areas such as Yemen, Iraq, the Democratic Republic of Congo, South Sudan, Ethiopia, Myanmar, and Central America.^1^ Several professional associations and the *Lancet* editorial board have highlighted the refugee crisis as a human rights imperative and matter of professional ethics.^2–5^ Thus, a pressing need exists for the medical community to be equipped to care for refugee patients, who may present with complex social, behavioral health, and medical conditions.

Refugee and asylee patients face unique challenges, such as experiences of trauma and torture, which may go underdiagnosed due to inadequate training of our health care workforce. A targeted cross-sectional study conducted in an urban primary care setting in the U.S. found that 6.6% of patients born outside the U.S. reported a history of experiencing torture, none of which had been previously identified by their primary physicians.^6^ Psychosocial stressors may disproportionately affect vulnerable populations, such as children, pregnant women, and women with infants.^7^ Migrant women may experience postpartum depression in up to 42% of cases or two to four times higher than in the general population.^8^ Family dynamics and psychosocial factors have been associated with failure to thrive.^9^

At the time of arrival to their country of resettlement, refugees are entitled to timely, comprehensive medical, behavioral health, and social services. The U.S. Centers for Disease Control and Prevention (CDC) have developed guidelines for both overseas medical screening that occurs in the country of resettlement application and domestic medical screening once refugees arrive on U.S. soil. That domestic refugee health screenings must comply with specific standards^10^ can become a barrier to clinician comfort with seeing refugee patients. Clinician discomfort with asking mental health questions or with transcultural trauma-informed care is another barrier.^11^ Furthermore, the needs of pediatric and teenage refugee patients often differ from those of adult patients.

Our educational workshop aims to build upon existing curricula and teaching tools to update and advance education and training for provision of family-centered pediatric refugee health care in any community setting. Recent trends in payment models have driven rapid change in health care delivery, specifically, team-based care, behavioral health integration in primary care, and the patient-centered medical home.^12,13^ Behavioral health integration is associated with higher quality and improved access.^14,15^ The National Institute of Medicine has issued a call to action for improved mental health for pediatric refugees that includes engaging primary care clinicians.^16^ A review of the literature reveals a gap in curricula or training for whole-person, culturally sensitive, trauma-informed care for refugee children with behavioral health integration. A clinical guide for family physicians is comprehensive but does not address pediatric-specific issues.^17^ A recently published trauma-informed care training focuses on mental health without simultaneous teaching on physical health.^18^ Pediatric-specific guidance combines immigrant and refugee children as compared to the unique prearrival experiences of refugee children.^19^ A recent pediatric publication offers clinical pearls as compared to our workshop format designed for active learning through synthesis and reflection.^20^

Additionally, there is often insufficient clinician knowledge of system-level factors that determine health care resource allocation for refugee patients.^21^ Indeed, the literature notes that insufficient clinician training contributes to barriers for refugees to access care and reduces the quality of care provided to this population.^22,23^ This is particularly a concern at a time of increasing closure of dedicated refugee clinics due to changes in allowed refugee quotas and subsequent loss of funding.^24,25^ Refugee patients previously cared for in specialized centers are increasingly being cared for in primary care settings. This underscores the need for training of all primary care clinicians in refugee care that is trauma informed and culturally sensitive.

While previous publications in *MedEdPORTAL* provide excellent, comprehensive guidance on refugee health, none focus on the specific needs of pediatric and adolescent refugee health.^26–29^ The intention is for this workshop to offer complementary materials to be used in combination with previous *MedEdPORTAL* publications. Additionally, the workshop content aligns with several of the Core Competencies defined by the Accreditation of Council for Graduate Medical Education: Patient Care, Medical Knowledge, Interpersonal and Communication Skills, Professionalism, and Systems-Based Practice.^30^

The conceptual framework for refugee health developed by Suphanchaimat, Kantamaturapoj, Putthasri, and Prakongsai^21^ identifies the following levels of refugee health care delivery:

(1) individual patient level, (2) care team level (e.g., clinicians, pharmacists, and others), (3) organisation or workplace level (e.g., hospital, clinic, nursing home, etc.), including infrastructure and complementary resources, and (4) societal level (e.g., legal framework, cultural value, and country economics).^21^

Our workshop addresses each of these four levels and incorporates recommended best practices described in a review article on providing primary health care for refugees and asylum seekers in high-income countries.^23^

## Methods

Three clinical case scenarios were developed according to established best practices for case-based learning.^31^ The Haiti case was loosely based on presenter experience. To ensure each case included aspects for whole-person care while offering a diversity of patient experiences, we included the following domains in each scenario: country of displacement, prearrival threats and conditions, refugee age, medical conditions, mental health, and cultural awareness. We selected a combination of lecture and case discussion to promote a common baseline of knowledge prior to group work for problem-solving together. Case studies allowed flexibility to adapt to various learner types (student, trainee, faculty) in multidisciplinary teams (physician, nurse, social worker, mental health professional, administrator, etc.), varying experience with refugee populations, customization to specific context (local factors, different clinical settings), and a range of time allowance for instruction. Cases were designed to create some ambiguity in order to generate inquiry, inference, debate, and reflection. This aligned with higher levels of cognition of Bloom's taxonomy.^32^

The workshop was intended for learners participating in health care delivery for refugees either as a direct care provider (e.g., physician, nurse practitioner, nurse) or as part of a multidisciplinary team (e.g., manager, public health professional, social worker, therapist). Besides conference settings, these materials could be presented in clinical or institutional settings.

We developed these workshop materials as a multi-institutional, multidisciplinary team with significant experience in humanitarian assistance, refugee health, health care delivery, public sector leadership, and research in low-resource countries. We represent a diverse set of roles across the levels and settings of refugee health care delivery, ranging from an academic setting to a 14-site, inner-city federally qualified health center (FQHC) with behavioral health integration. The first author, a former Medicaid medical director, served as the chief medical officer of the FQHC acting as a designated refugee screening site in New York City, New York, embedded within a primary care clinic. One author is director of education and site director in a community health setting. The senior author directs a global health course and has overseen health initiatives in over a dozen conflict-affected regions. The narratives of the Honduras and Somalia cases were built on real examples described by the Women's Refugee Commission and partners.^33,34^

The audiovisual needs of the workshop included a screen, LCD projector, and laptop. An internet connection was not required. There should be either round tables with six to eight participants per table or chairs that can be arranged in circles for small-group discussions. A flip chart or whiteboard with markers is recommended.

The workshop agenda (Appendix A) outlined the workshop's content and schedule. In future offerings of the workshop, if group size allows, participants could also be invited to introduce themselves and describe any experience or interest in the topic. These self-introductions could be especially helpful in multidisciplinary settings where prior experience, perspectives, or baseline knowledge may differ.

We began with introductions, followed by sharing the workshop objectives and schedule. The first presentation (Appendix B) focused on the experiences and sources of trauma that refugees might experience prior to arrival in the host country. These traumas can be severe and include imprisonment, torture, loss of property, malnutrition, sexual violence, loss of livelihood, separation from family and friends, witnessing or surviving violence, and difficult living conditions.

This discussion was followed by a presentation that described common medical conditions faced by refugees (Appendix C), reviewed the CDC's domestic medical screening guidelines, and discussed evidence-based management of commonly seen conditions. This presentation prompted a group discussion of some challenges related to determining vaccination status, overcoming cost barriers to care, and the importance of shared decision-making when addressing the needs of newly arrived refugees.

The next portion of the workshop involved a small-group exercise. We assigned participants to one of three groups to discuss specific clinical scenarios, with each group having a unique case (Appendix D). We facilitated the small-group exercise (30 minutes) with one to two workshop facilitators at each table. This was followed by a large-group report-back and debrief (30 minutes).

The subsequent presentation discussed the importance of providing trauma-informed care when addressing the needs of at-risk children (Appendix E). This discussion described the SAFE model, developed by Betancourt and colleagues, which emphasizes a holistic and integrated approach to providing care for vulnerable children.^35,36^ The SAFE model identifies four equally important and interlocked needs, namely, (1) safety or freedom from harm, (2) access to basic physiological needs and health care, (3) family and connection to others, and (4) education and economic security. During this presentation, each of these four needs was described and illustrated by real-world examples among children affected by conflict and disaster.

The final part of the workshop reviewed policy, advocacy, and outreach (Appendix F). The latter included a section on sustainability of programs through assessment of funding and operational challenges as well as personal sustainability through self-care and communities of support. Partnership agencies and organizations, especially for community support or legal advising, were identified. We concluded the workshop with the development of a personal advocacy plan that set short- and long-term goals for engaging in refugee health advocacy, with goals at 3 weeks, 3 months, and 6 months. Finally, we invited open comments on the session.

The workshop was evaluated in multiple ways. Conference evaluations were collected electronically by conference organizers using a 5-point evaluation form standardized across all conference sessions. This conference-administered evaluation also provided space for open-ended comments from participants. North American Refugee Health Conference (NAHRC) organizers collected standardized paper-based evaluations that also used a 5-point scale.

Since standardized conference evaluations may not offer specific feedback tailored to the session topic or format and since there is often historically low response to conference-administered evaluations, we developed an additional evaluation (Appendix G) and invited participants to complete pre- and postworkshop self-assessments related to their understanding and comfort with the workshop content. This second facilitator-administered evaluation form was distributed at the beginning of the workshop and completed anonymously. Participants were asked to rate the overall quality of the workshop on a 5-point scale (1 = *poor,* 5 = *outstanding*). For qualitative feedback, participants were asked, “Please provide any feedback about the workshop (e.g., what worked well, what could be improved, etc.).”

## Results

This workshop was peer-reviewed and accepted for the Pediatric Academic Societies (PAS) 2019 meeting. In 2019, of 388 workshops submitted, only 85 (22%) were accepted for presentation. Additionally, the workshop was selected for presentation at the 2019 NARHC. For the latter, the content was shortened to fit the available 1 hour. In order to condense the materials into a 1-hour workshop and because it was assumed NARHC attendees would have more experience with refugee health, we did not include the PAS background presentations on refugees and trauma-informed care. Instead, the 1-hour workshop covered the integration of refugee health care in a community setting, the CDC's domestic medical screening guidelines, the three case studies with minor adaptations based on feedback from PAS, and advocacy. Conference- and facilitator-administered evaluations were similarly conducted for this workshop using the conference organizers' paper form and our previously discussed evaluation form.

### PAS Workshop

Seventeen learners participated in the 3-hour PAS workshop in April 2019, including a mix of clinicians, nurse practitioners, pediatric residents, medical students, and undergraduate students. Five participants (29%) completed the conference-administered evaluations, which showed a score of 4.8 for the overall quality of the workshop, on a 5-point scale (5 = *outstanding*; Table 1).

**Table 1.**
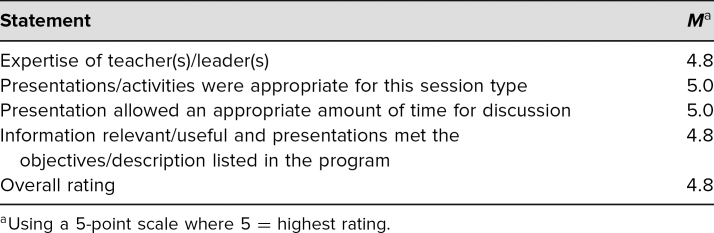
Results of Evaluation Conducted by Pediatric Academic Societies Meeting Organizers (*n* = 5)

Eleven participants completed the pre- and postworkshop evaluations administered by the PAS workshop facilitators (Table 2). On a 5-point scale (5 = highest rating), workshop participants evaluated their comfort with taking care of refugee patients as 3.3 before and 4.0 after the workshop (*p* = .24). They reported their understanding of refugees' prearrival experiences as 3.0 before and as 3.6 (*p* = .19) after the workshop and rated their understanding of screenings as 2.7 before and 3.6 after the workshop (*p* = .13). While change in each of these questions trended toward statistical significance, the difference in participant comfort with asking about the social and behavioral health of refugee patients was statistically significant and increased from 2.9 before the workshop to 4.2 after the workshop (*p* = .04).

**Table 2.**
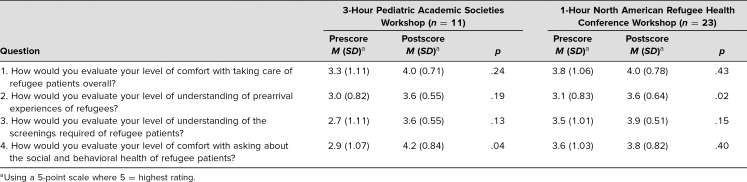
Results of Additional Evaluations Conducted by Workshop Facilitators

### NARHC Workshop

The NARHC workshop was attended by more than 30 participants, about 80% of whom were clinicians, with the others being policy makers and public health professionals. The conference-administered evaluations were completed by 22 (over 70%) participants. The mean overall rating for the NARHC workshop was 4.2 on a 5-point scale (5 = *excellent*; Table 3).

**Table 3.**
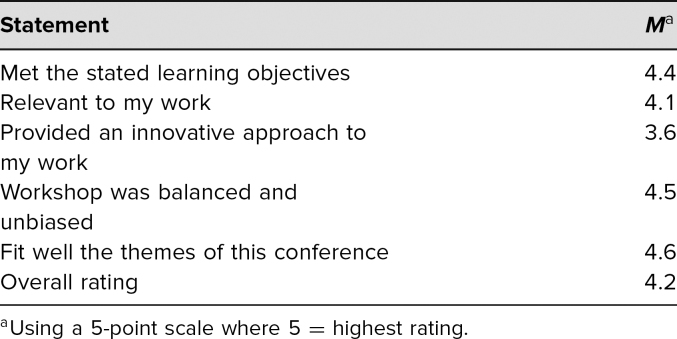
Results of Evaluation Conducted by North American Refugee Health Conference Meeting Organizers (*n* = 22)

Twenty-three participants completed the 5-point, facilitator-administered evaluation forms before and after the workshop. For all four self-assessment questions, NARHC workshop participants rated their preworkshop understanding and comfort related to the workshop topic higher than did PAS workshop participants. Nevertheless, all postworkshop self-assessments responses increased in both workshops. NARHC workshop participants evaluated their comfort with taking care of refugee patients as 3.8 before and 4.0 after the workshop (*p* = .43; Table 3). They assessed their understanding of refugees' prearrival experiences as 3.1 before and 3.6 after the workshop (*p* = .02) and rated their understanding of health screenings as 3.5 before and 3.9 after the workshop (*p* = .15). Their comfort with asking about the social and behavioral health of refugee patients increased from 3.6 before to 3.8 after the workshop (*p* = .40).

Additionally, 10 individuals provided anonymous open-response feedback in the evaluations after the two workshops. The majority of responses were positive and described the workshop as “easy to understand,” “excellent preparation and presentation of various challenges of refugee health,” and “helpful to hear how others handle things in different settings.” Participants were particularly pleased with the case studies: “I really think case studies are a good tool to facilitate understanding of the guidance,” “I am a nonclinician. Case-studies were helpful.” However, one NARHC participant reported that a portion of the 1-hour workshop felt rushed, likely as a consequence of the workshop's more compressed time line.

## Discussion

The workshop was evaluated in two settings with a diverse group of participants with different roles, levels of training, practice settings, and baseline knowledge of or experience with refugee health. The positive trend to the results implies this workshop could be applied and disseminated broadly. However, the sample size was relatively small and not powered to show many statistically significant differences between pre/post testing. The response result was higher in the NARHC attendees, allowing a more robust evaluation of this more specialized audience with better baseline knowledge and comfort regarding refugee health. However, given the higher baseline and smaller incremental change, the NARHC group was also underpowered to achieve significance. Thus, further evaluation would be required to assess effectiveness. Additionally, long-term impact and retention were not assessed.

Recommended adaptations fo or additions to this material include adding teaching on nutritional status and a robust exploration of legal needs of refugees, as well as the nuances for asylees, who are not granted the same protections as refugees. It is also recommended to invite an individual from a local legal aid agency or refuge advocacy group. This would serve to add content as well as foster relationships, promote coordination, and create partnerships at the local level. We presented the three cases simultaneously, which served to reduce the exposure of any single learner to the full discussion on each case. A recommended adaptation, if time allows, is to present this workshop as a lecture series or minicourse over several weeks or months. The added benefit would be to reduce cognitive load by spacing out learning and promoting additional time for deeper reflection.

Strengths of this workshop include a format that can easily be adapted for different settings, time allowances, and audiences. The design of the workshop does not require specialized training of facilitators, making it more easily implemented. The workshop is based on a multilevel framework for health care delivery. This approach takes into account the range from individual-level factors to system-level factors that affect effectiveness, quality, and sustainability of refugee health care, especially at a time of rapid and unpredictable changes to resources. Discussions allow exploration of clinician barriers to and comfort with integrating refugee care into general primary care. Additionally, there are challenging logistics of incorporating unique aspects of refugee health care into primary care work streams and operations. Among attendees at PAS were a medical clinic director, a residency director, and the dean of a medical school, indicating an interest among health care administrators in pragmatic tools to better serve refugee populations.

A challenge encountered during the workshop's guided discussions related to the diversity of participants at the international meetings where the workshop was conducted. If participants are from a single institution or location, this may be reduced. Participants represented a range of practice settings and differing policies across states or countries. A particular challenge was state-by-state variation of Medicaid in the U.S. as compared to countries with national health systems. However, such challenges also can become opportunities for participants to learn about other systems of care. Nonetheless a suggestion to future facilitators is to consider assigning participants to groups by similar clinical settings or geography for better sharing of applicable best practices. Another challenge is the amount of time allotted for discussion of a complex and sensitive topic. Self-reflection to identify personal fears or biases regarding care to refugees may need a longer time, smaller groups, or additional methods.

A limitation is that our workshops were presented at conferences with voluntary attendance. Thus, we evaluated a self-selected group of participants with interest or experience in refugee health. If the workshop is conducted as an assigned part of a curriculum, facilitators may need to be more proactive in eliciting engagement or responses. We wrote the facilitators' guide to include many potential prompts to support robust discussion. Another limitation was time constraints of a 3-hour workshop. Our intent was to cover the essential knowledge base while allowing sufficient time to discuss and explore trauma-informed care, behavioral health integration, and cultural respect. In instances where there might be insufficient time for the full 3-hour workshop offering, a portion can be presented rather than the full content. Facilitators may choose which section(s) might be of greatest relevance to their target audience. Participants seemed to enjoy the case-scenario discussions but suggested even more information could be provided in each case regarding the patient's health status (e.g., height, weight, laboratory values) and legal status (e.g., visa, unregistered). While more information on patient health status was added to the case studies for NARHC, the feedback highlights important areas for further development or a need for use of complementary materials. The Refugee Health Elective materials published by Stone and colleagues in *MedEdPORTAL* provide more in-depth teaching and case-based learning, while Fitzgerald and colleagues offer robustly evaluated materials with a focus on culturally competent care.^26,29^

Potential future directions include adding more domains to the cases and expanding the time allotted for discussion. Additionally, the delivery and evaluation of the workshop could be adapted for an online format with videoconferencing. This could reduce barriers to access at community sites that may not be able to send attendees to national conferences. Online teaching would align with the change in medical education that has occurred with the COVID-19 pandemic.^37^

At a time of historic increases in refugees and displaced persons plus constricted funding for dedicated refugee health care and reduction of stand-alone refugee clinics, the development and dissemination of easily accessible training materials that can be applied in any setting across the U.S. are of particular importance.

## Appendices

Agenda.docxPresentation 1 Intro to Refugees.pptxPresentation 2 Health Screening.pptxCases.docxPresentation 3 Trauma-Informed Care.pptxPresentation 4 Refugee Health Advocacy.pptxRefugee Workshop Evaluation.docx
All appendices are peer reviewed as integral parts of the Original Publication.
