# Supplementary material for: Pediatric Refugee Health Care Delivery in the Community Setting: An Educational Workshop for Multidisciplinary Family-Centered Care During Resettlement
Source: MedEdPORTAL. 2020 Nov 3;16:10988. doi: 10.15766/mep_2374-8265.10988 (PMC7666829; doi:10.15766/mep_2374-8265.10988)
Supplement: Supplementary file 1 — Agenda.docxPresentation 1 Intro to Refugees.pptxPresentation 2 Health Screening.pptxCases.docxPresentation 3 Trauma-Informed Care.pptxPresentation 4 Refugee Health Advocacy.pptxRefugee Workshop Evaluation.docx [file mep_2374-8265.10988-s001.zip › A. Agenda.docx]

**Delivering Refugee Healthcare in a Community Setting: Workshop Agenda**

1. Introduction (10 minutes)

2. Who are the refugees? (15 minutes)

3. Refugee medical screenings (20 minutes)

4. Breakout session: Clinical care scenarios (60 minutes)

a. Central America

b. Somalia

c. Haiti

5. Trauma-informed care (20 minutes)

6. Advocacy and outreach in refugee health (20 minutes)

7. Introduction to advocacy, policy, social media (20 minutes)

8. Reflections (15 minutes)
