# Supplementary material for: Pediatric Refugee Health Care Delivery in the Community Setting: An Educational Workshop for Multidisciplinary Family-Centered Care During Resettlement
Source: MedEdPORTAL. 2020 Nov 3;16:10988. doi: 10.15766/mep_2374-8265.10988 (PMC7666829; doi:10.15766/mep_2374-8265.10988)
Supplement: Supplementary file 1 — Agenda.docxPresentation 1 Intro to Refugees.pptxPresentation 2 Health Screening.pptxCases.docxPresentation 3 Trauma-Informed Care.pptxPresentation 4 Refugee Health Advocacy.pptxRefugee Workshop Evaluation.docx [file mep_2374-8265.10988-s001.zip › B. Presentation 1 Intro to Refugees.pptx]

## Slide 1
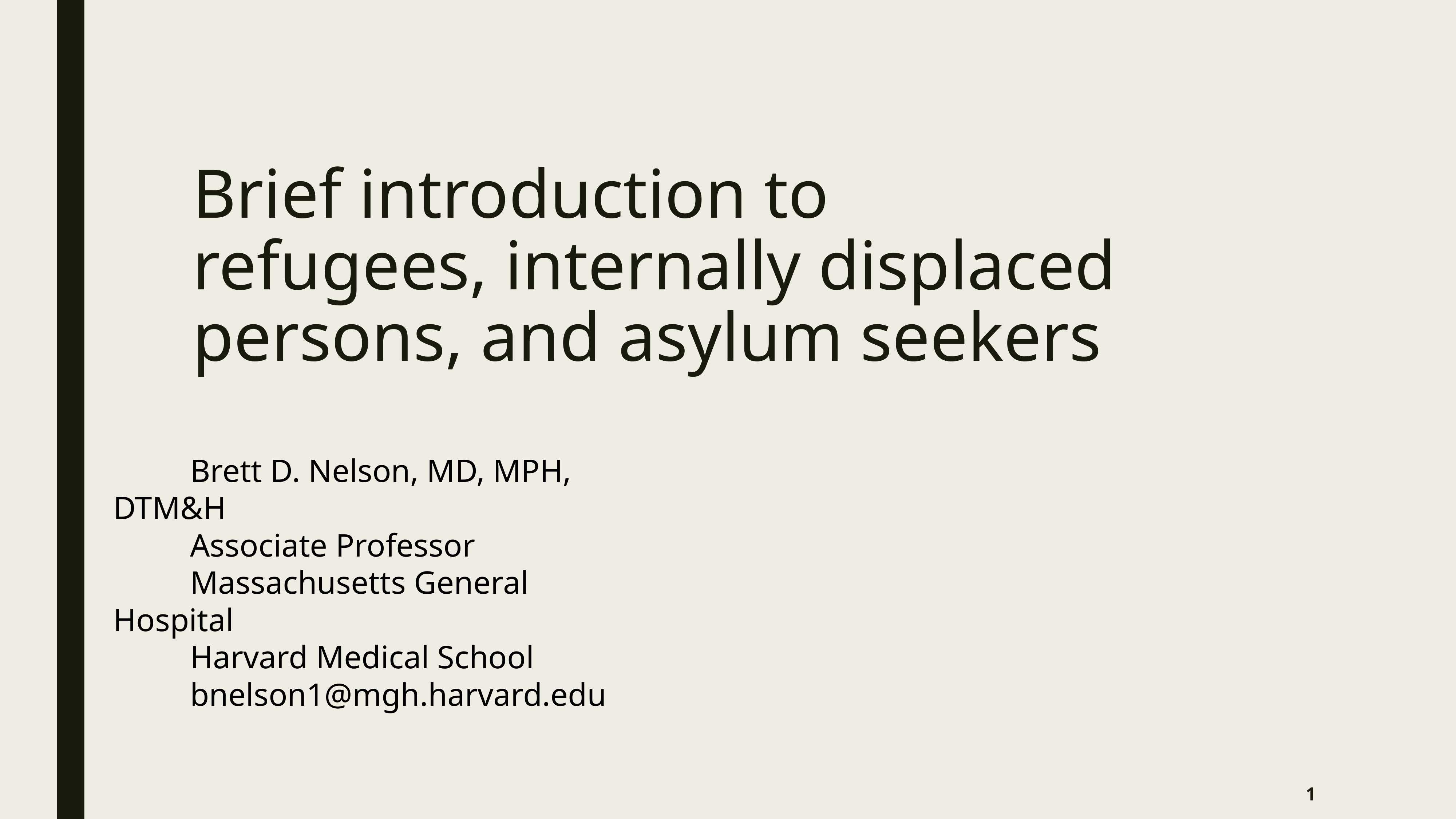

# Brief introduction to refugees, internally displaced persons, and asylum seekers
Brett D. Nelson, MD, MPH, DTM&H
Associate Professor
Massachusetts General Hospital
Harvard Medical School
bnelson1@mgh.harvard.edu
1

## Slide 2
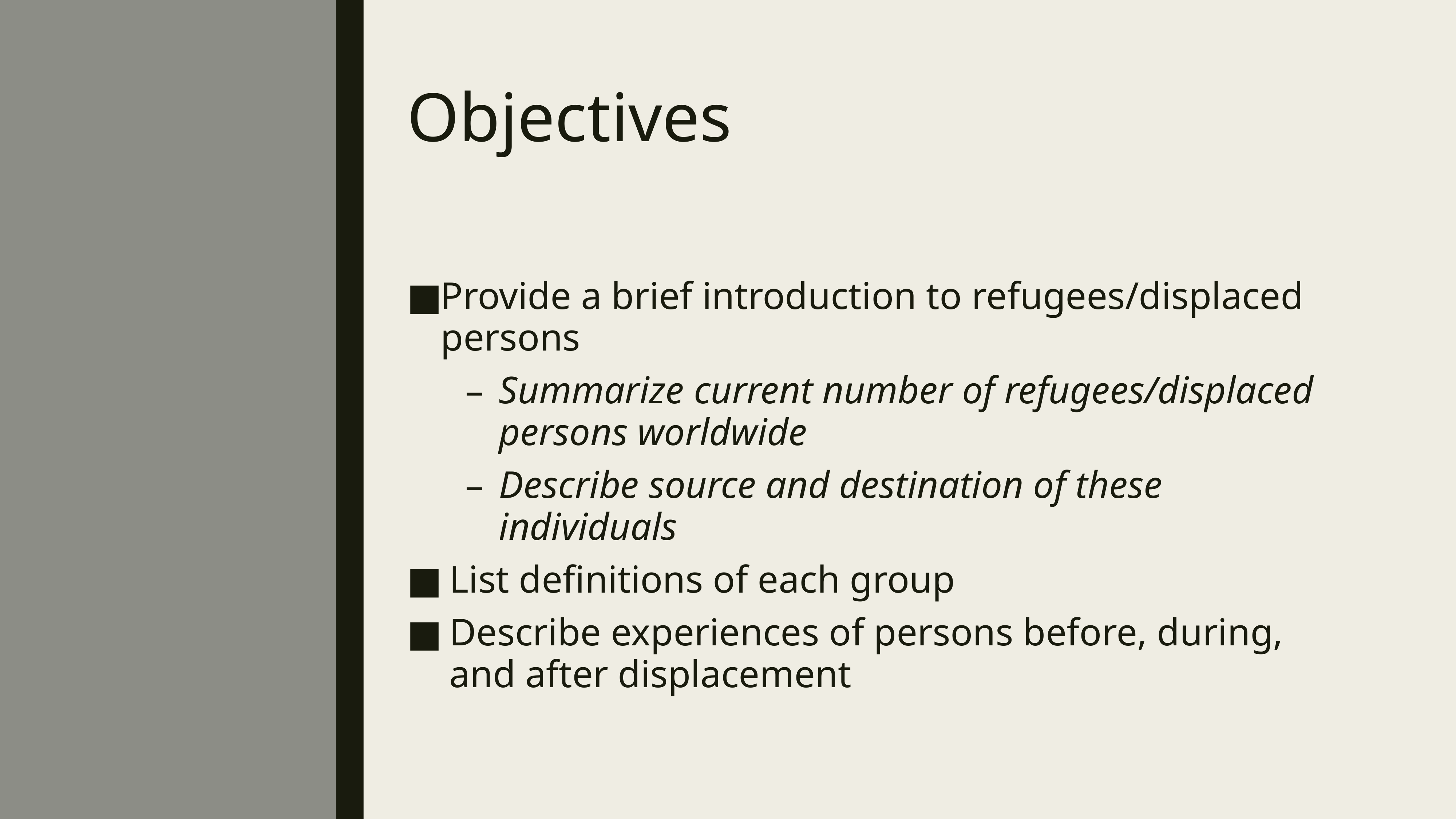

# Objectives
Provide a brief introduction to refugees/displaced persons
Summarize current number of refugees/displaced persons worldwide
Describe source and destination of these individuals
List definitions of each group
Describe experiences of persons before, during, and after displacement
2

## Slide 3
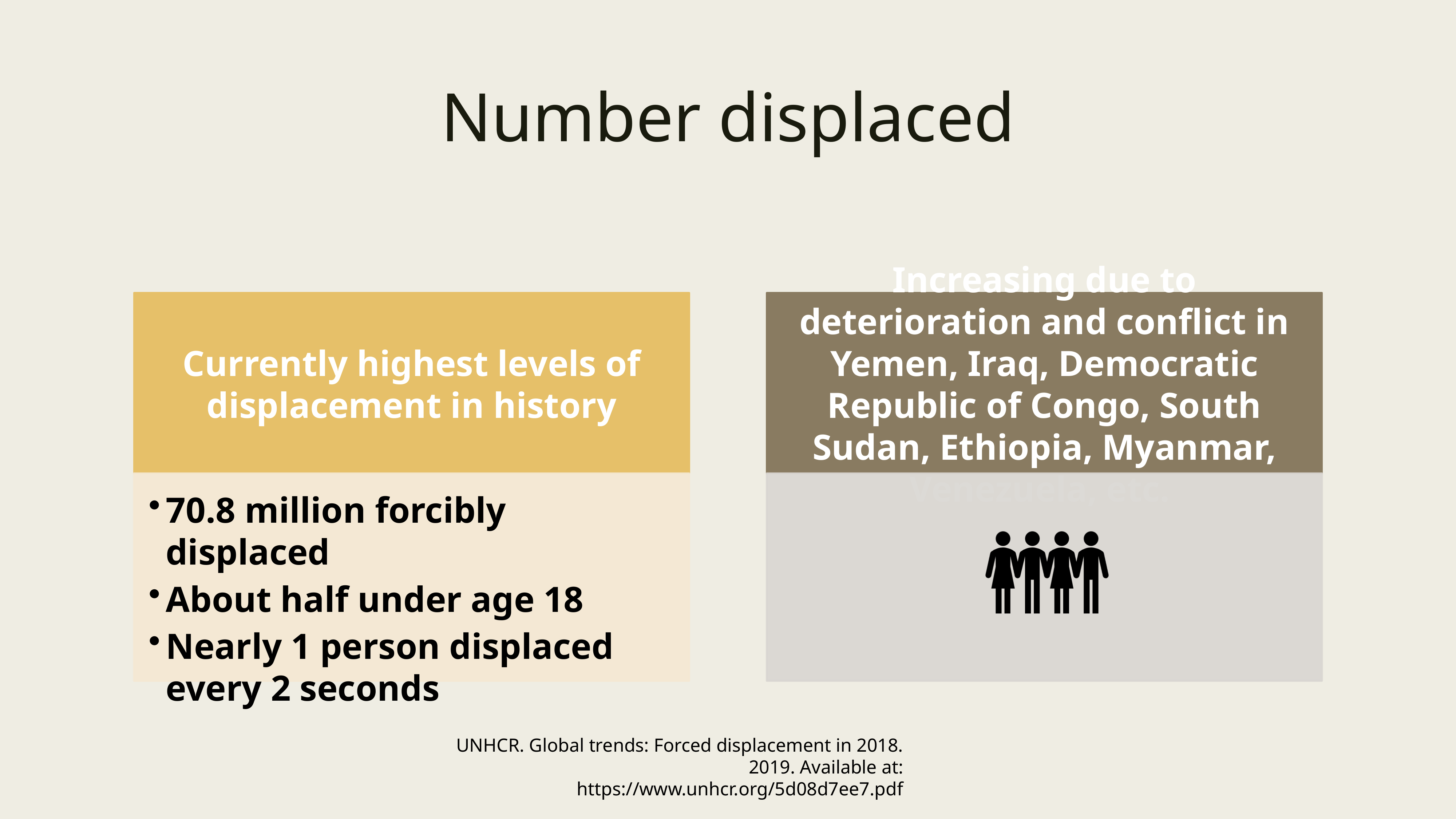

# Number displaced
UNHCR. Global trends: Forced displacement in 2018. 2019. Available at: https://www.unhcr.org/5d08d7ee7.pdf
3

## Slide 4
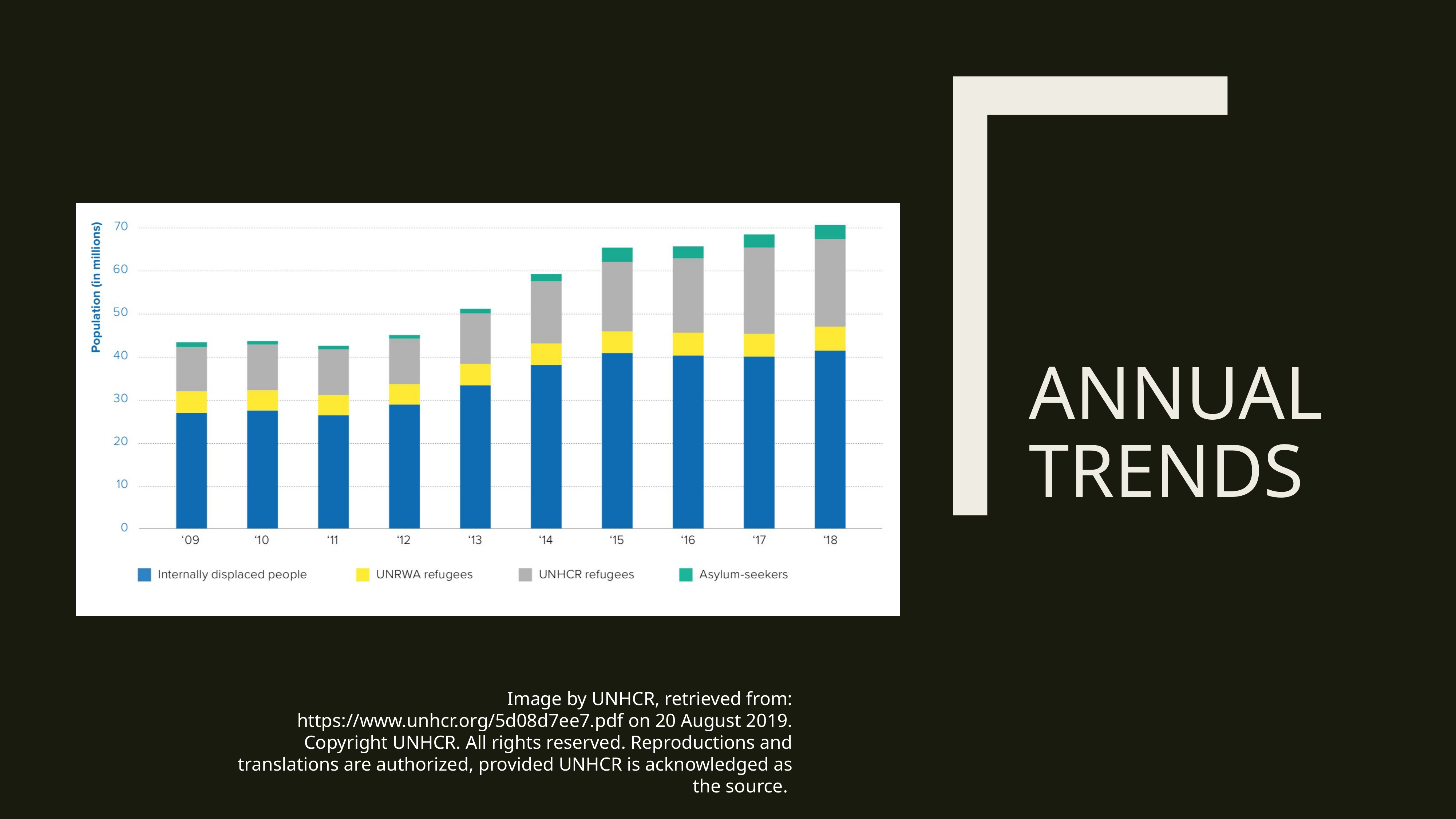

# Annual trends
Image by UNHCR, retrieved from: https://www.unhcr.org/5d08d7ee7.pdf on 20 August 2019. Copyright UNHCR. All rights reserved. Reproductions and translations are authorized, provided UNHCR is acknowledged as the source.
4

## Slide 5
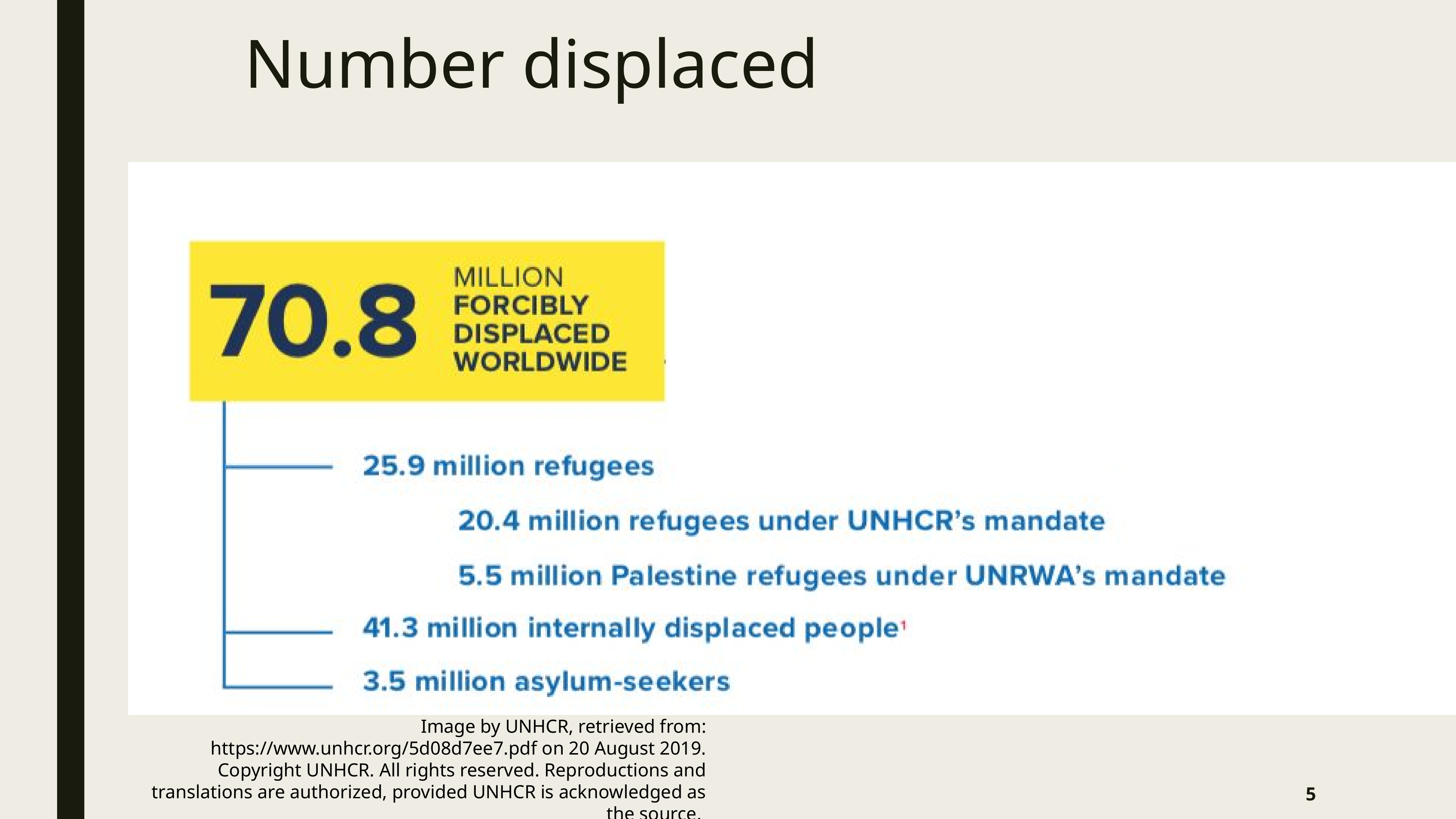

# Number displaced
Image by UNHCR, retrieved from: https://www.unhcr.org/5d08d7ee7.pdf on 20 August 2019. Copyright UNHCR. All rights reserved. Reproductions and translations are authorized, provided UNHCR is acknowledged as the source.
5

## Slide 6
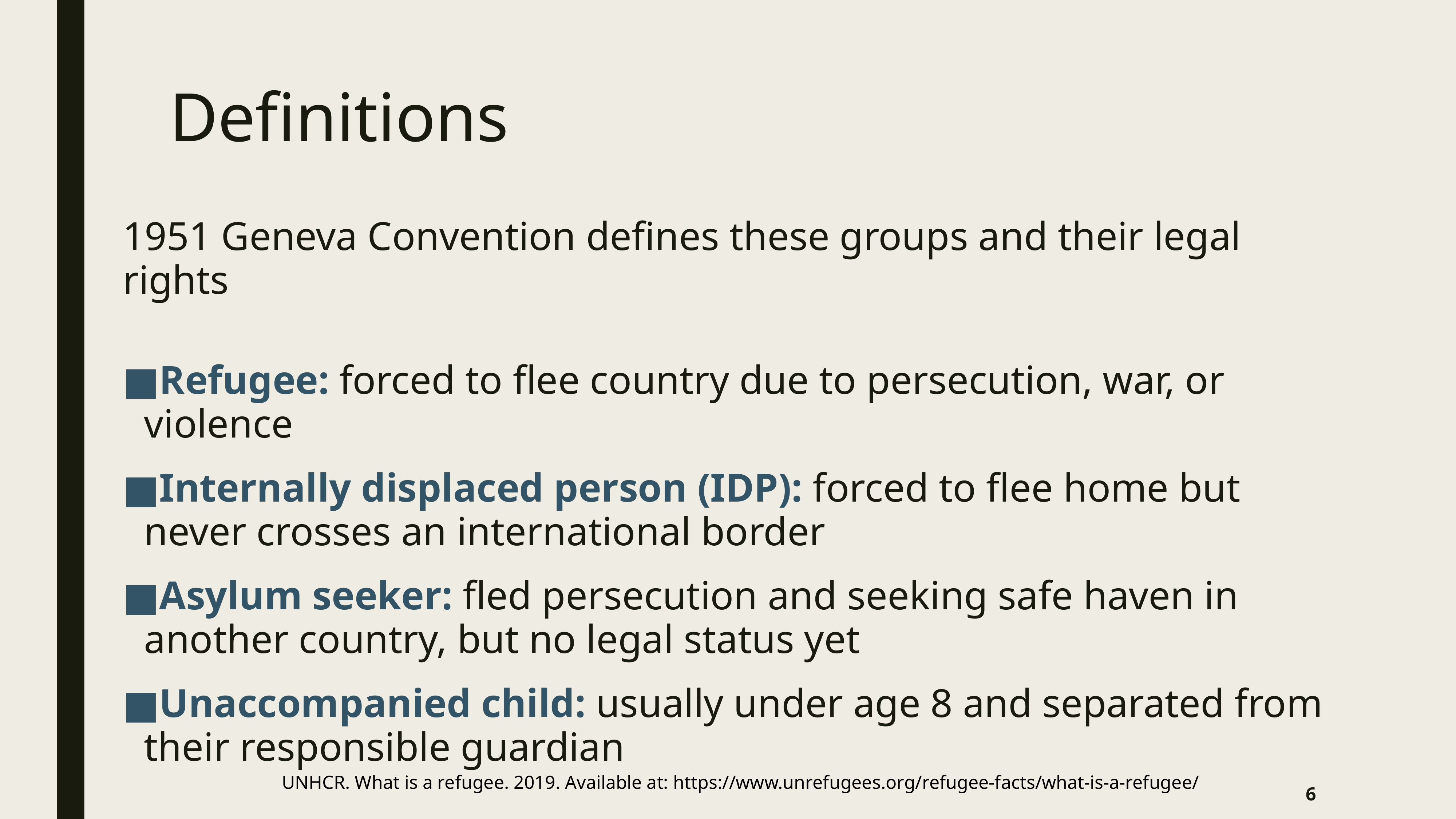

# Definitions
1951 Geneva Convention defines these groups and their legal rights
Refugee: forced to flee country due to persecution, war, or violence
Internally displaced person (IDP): forced to flee home but never crosses an international border
Asylum seeker: fled persecution and seeking safe haven in another country, but no legal status yet
Unaccompanied child: usually under age 8 and separated from their responsible guardian
UNHCR. What is a refugee. 2019. Available at: https://www.unrefugees.org/refugee-facts/what-is-a-refugee/
6

## Slide 7
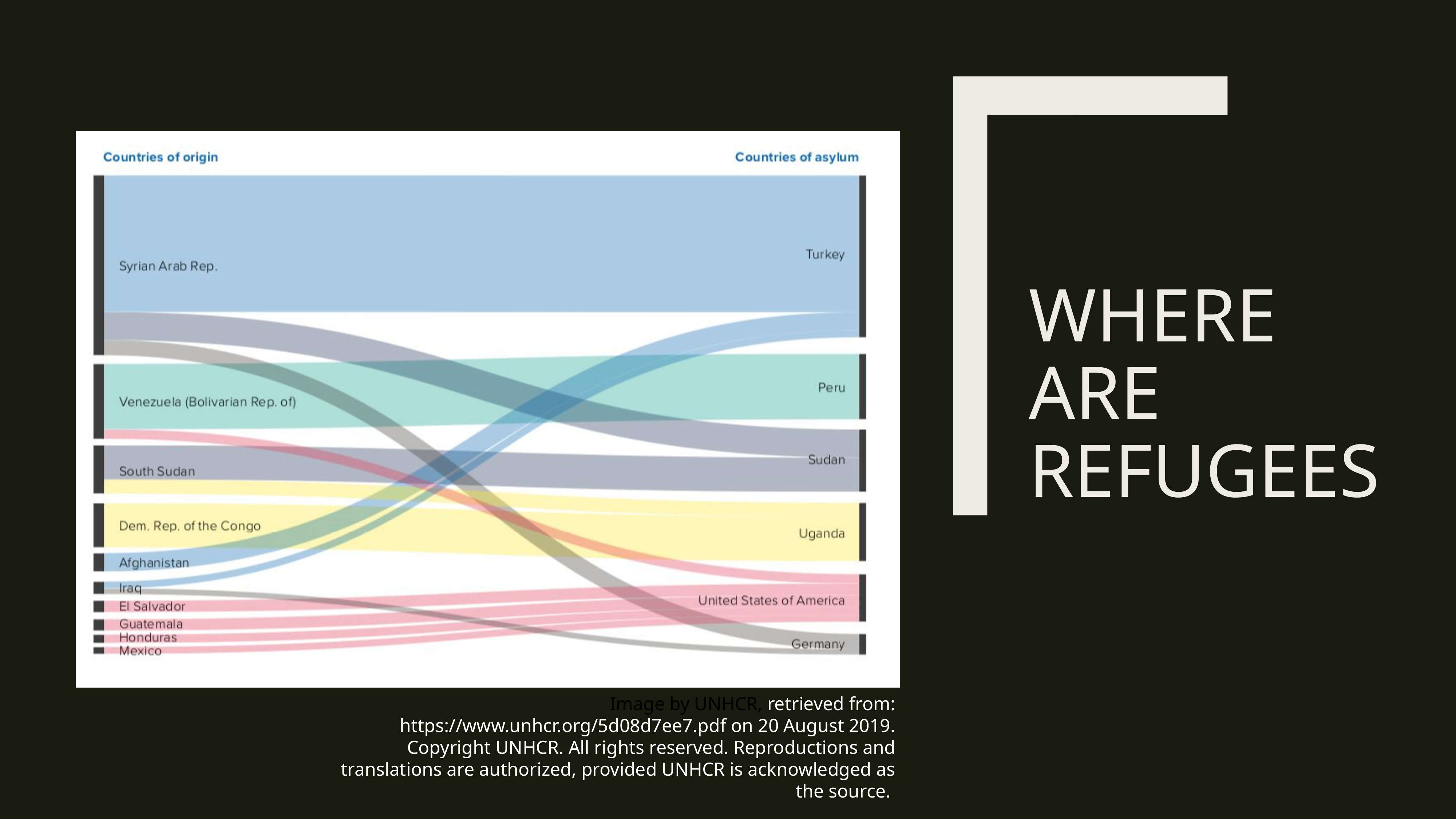

# Where are refugees
Image by UNHCR, retrieved from: https://www.unhcr.org/5d08d7ee7.pdf on 20 August 2019. Copyright UNHCR. All rights reserved. Reproductions and translations are authorized, provided UNHCR is acknowledged as the source.
7

## Slide 8
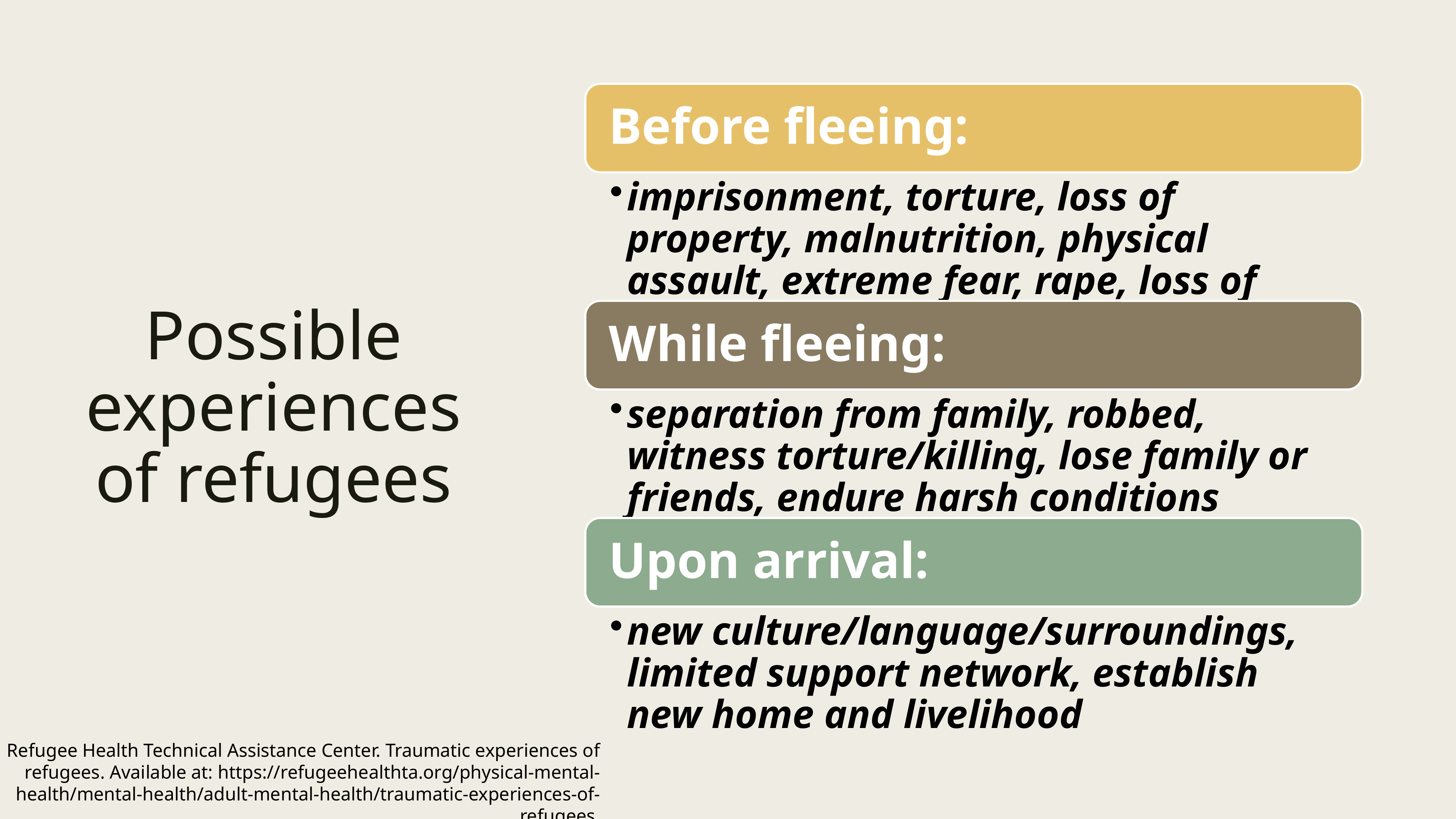

# Possible experiences of refugees
Refugee Health Technical Assistance Center. Traumatic experiences of refugees. Available at: https://refugeehealthta.org/physical-mental-health/mental-health/adult-mental-health/traumatic-experiences-of-refugees.
8

## Slide 9
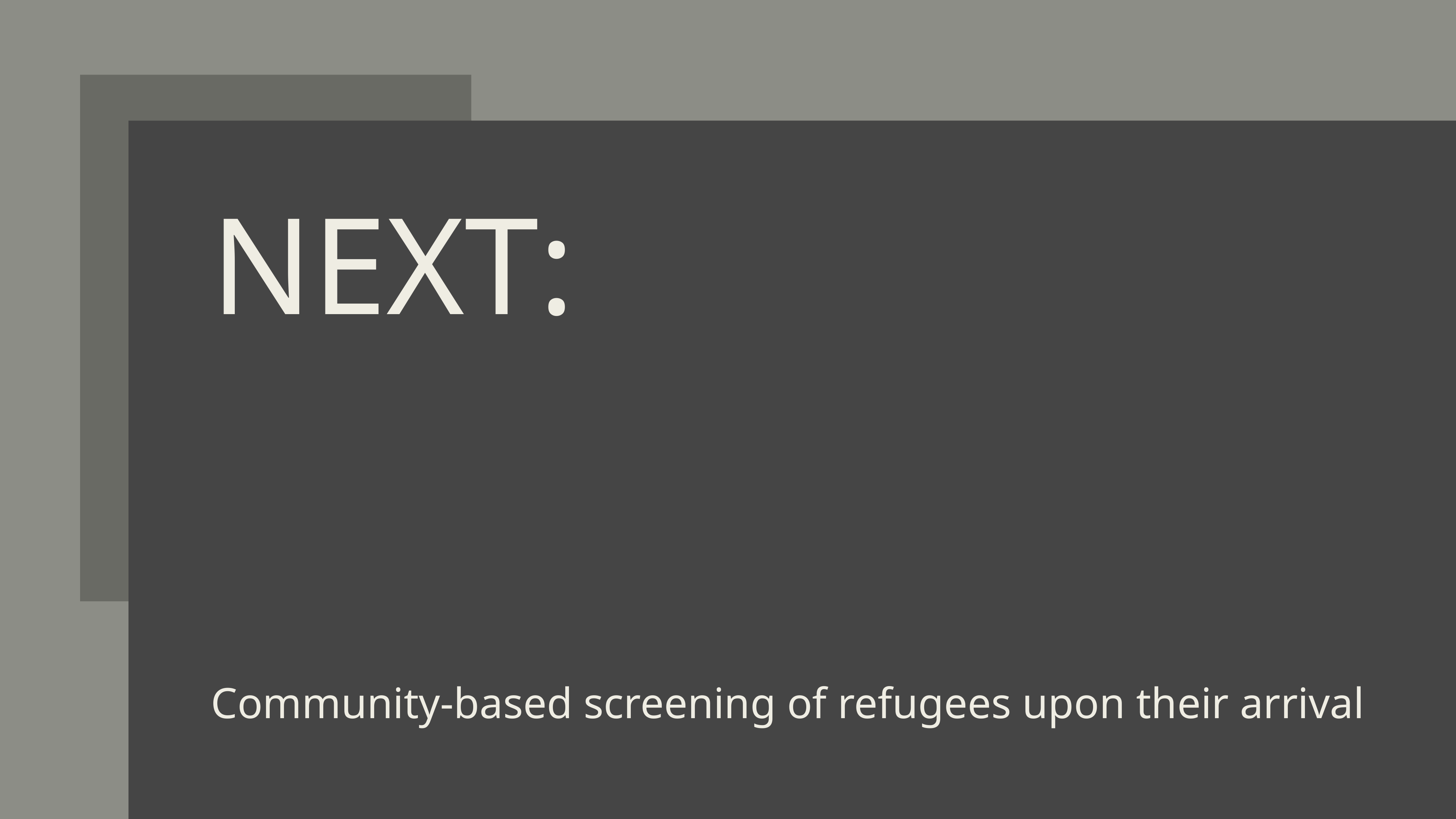

# Next:
Community-based screening of refugees upon their arrival
9
