# Supplementary material for: Pediatric Refugee Health Care Delivery in the Community Setting: An Educational Workshop for Multidisciplinary Family-Centered Care During Resettlement
Source: MedEdPORTAL. 2020 Nov 3;16:10988. doi: 10.15766/mep_2374-8265.10988 (PMC7666829; doi:10.15766/mep_2374-8265.10988)
Supplement: Supplementary file 1 — Agenda.docxPresentation 1 Intro to Refugees.pptxPresentation 2 Health Screening.pptxCases.docxPresentation 3 Trauma-Informed Care.pptxPresentation 4 Refugee Health Advocacy.pptxRefugee Workshop Evaluation.docx [file mep_2374-8265.10988-s001.zip › C. Presentation 2 Health Screening.pptx]

## Slide 1
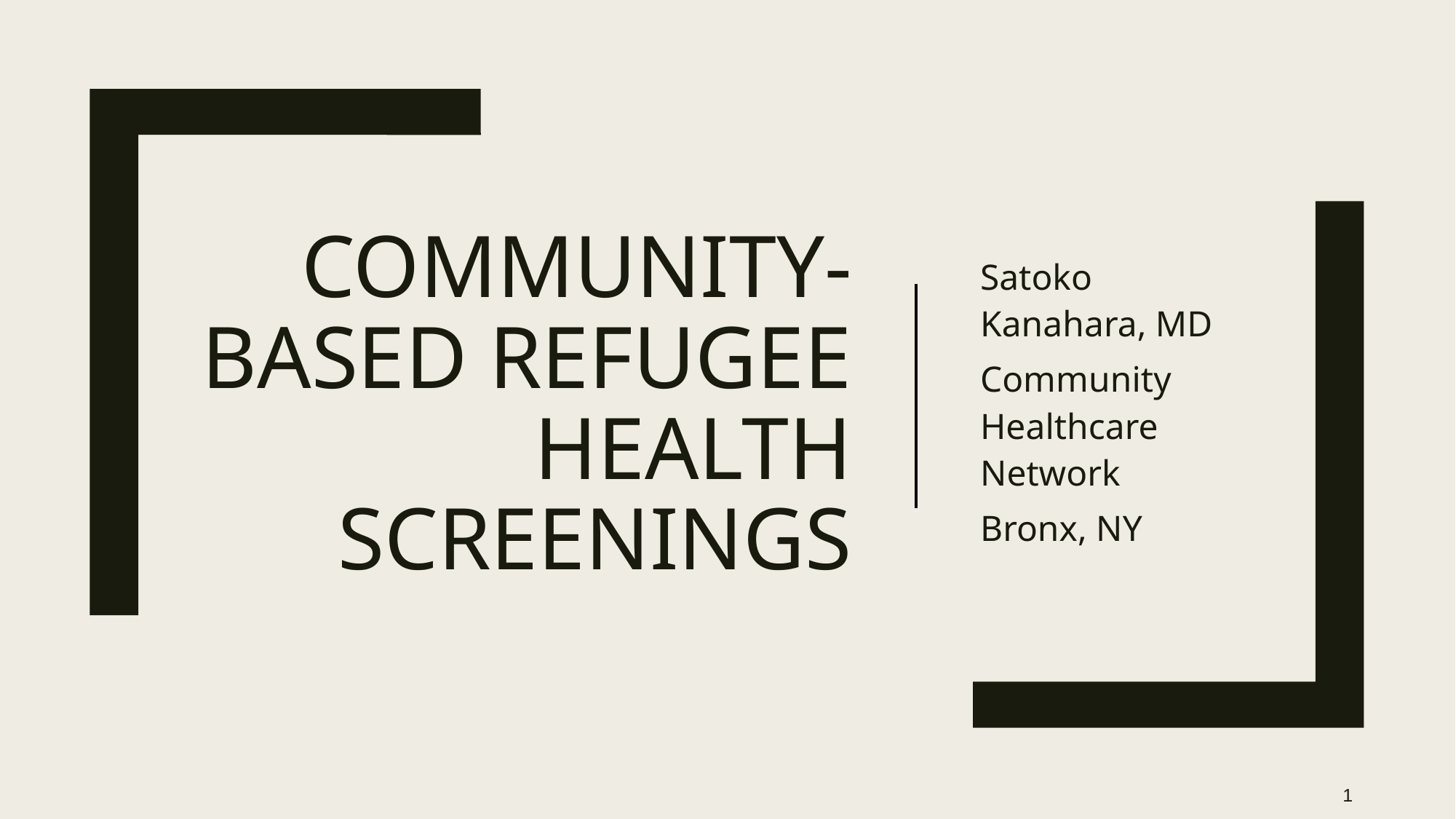

Satoko Kanahara, MD
Community Healthcare Network
Bronx, NY
# Community-based Refugee Health Screenings
1

## Slide 2
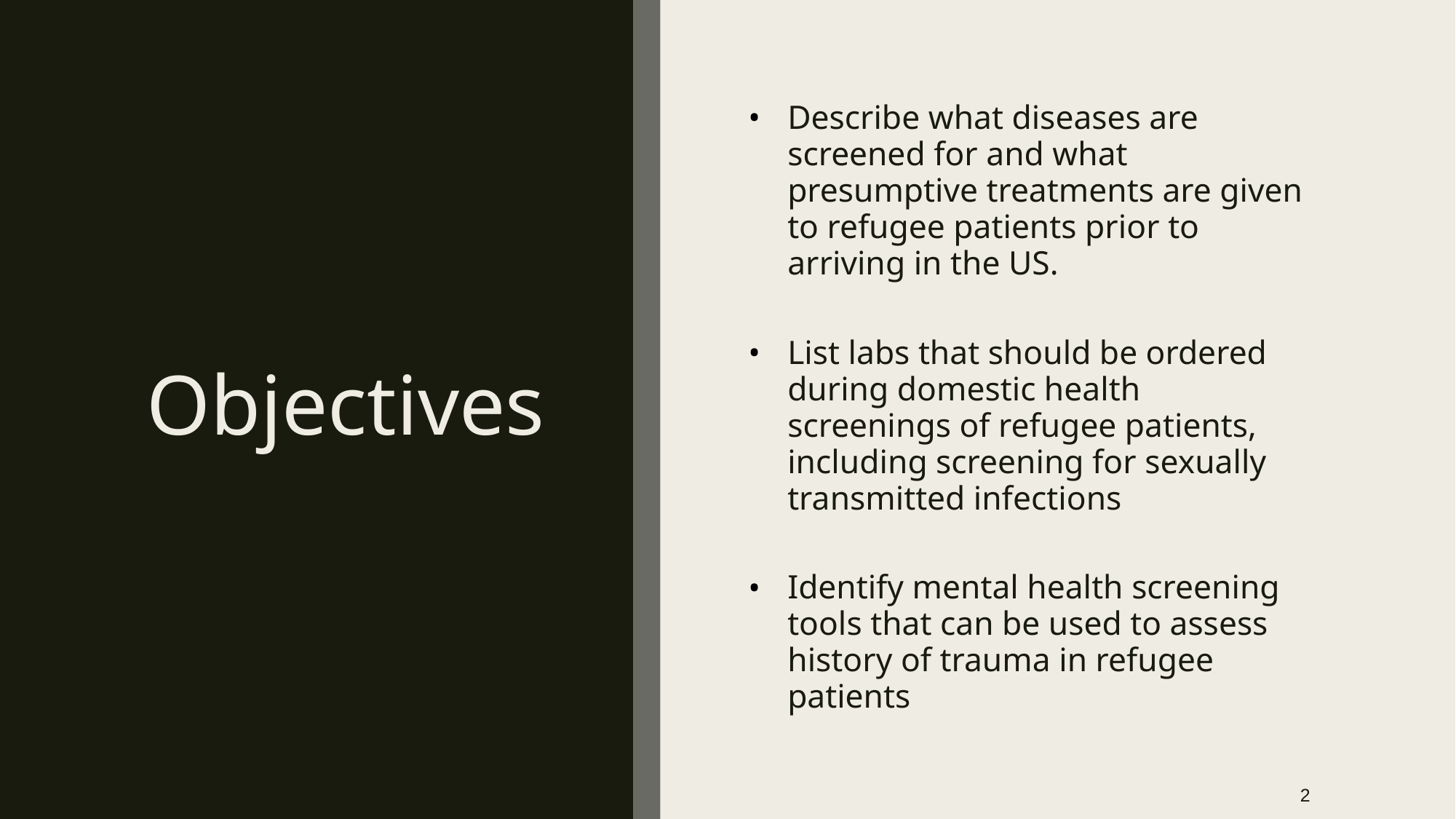

# Objectives
Describe what diseases are screened for and what presumptive treatments are given to refugee patients prior to arriving in the US.
List labs that should be ordered during domestic health screenings of refugee patients, including screening for sexually transmitted infections
Identify mental health screening tools that can be used to assess history of trauma in refugee patients
2

## Slide 3
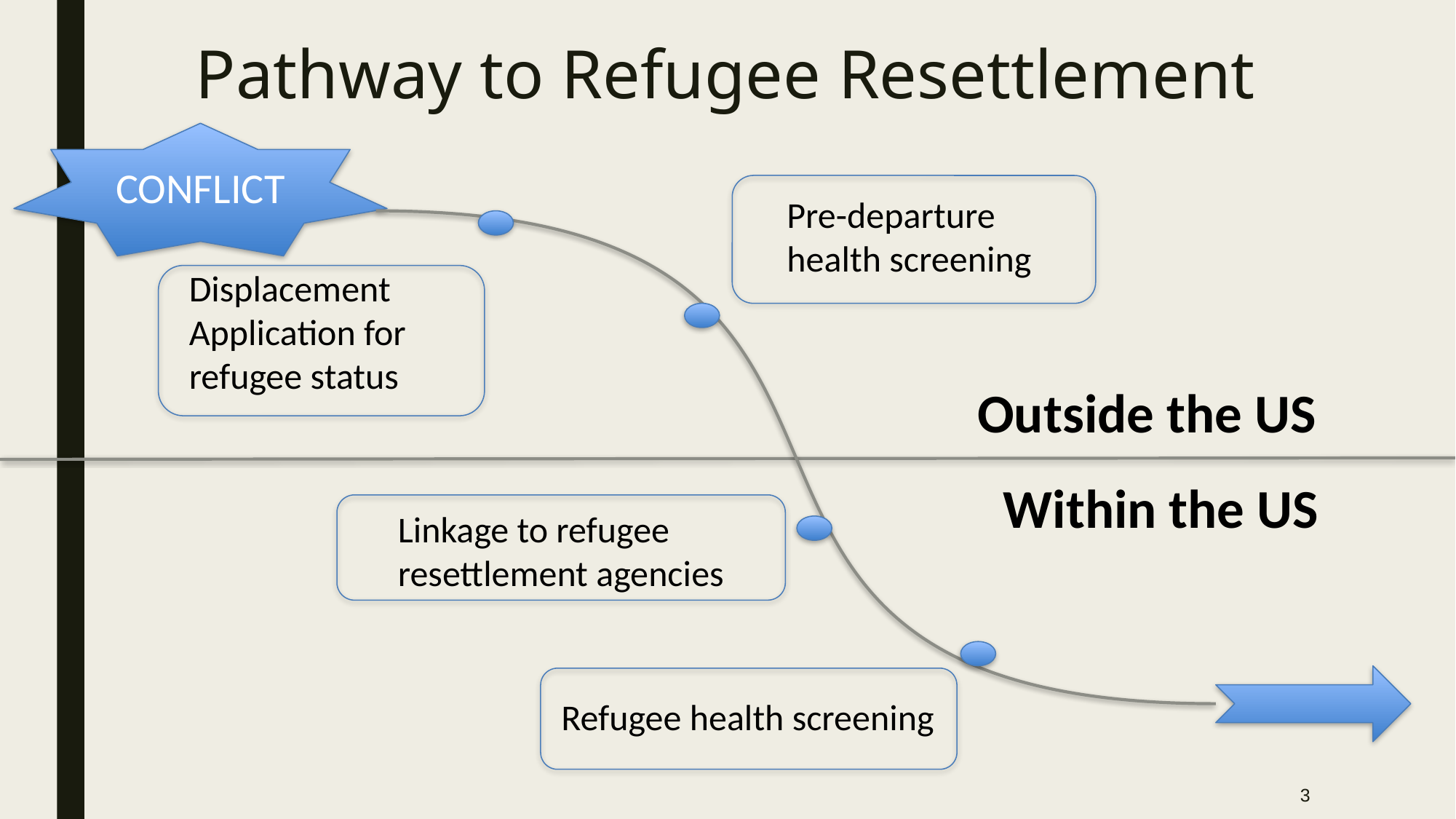

# Pathway to Refugee Resettlement
CONFLICT
Pre-departure health screening
Displacement Application for refugee status
Outside the US
Within the US
Linkage to refugee resettlement agencies
Refugee health screening
3

## Slide 4
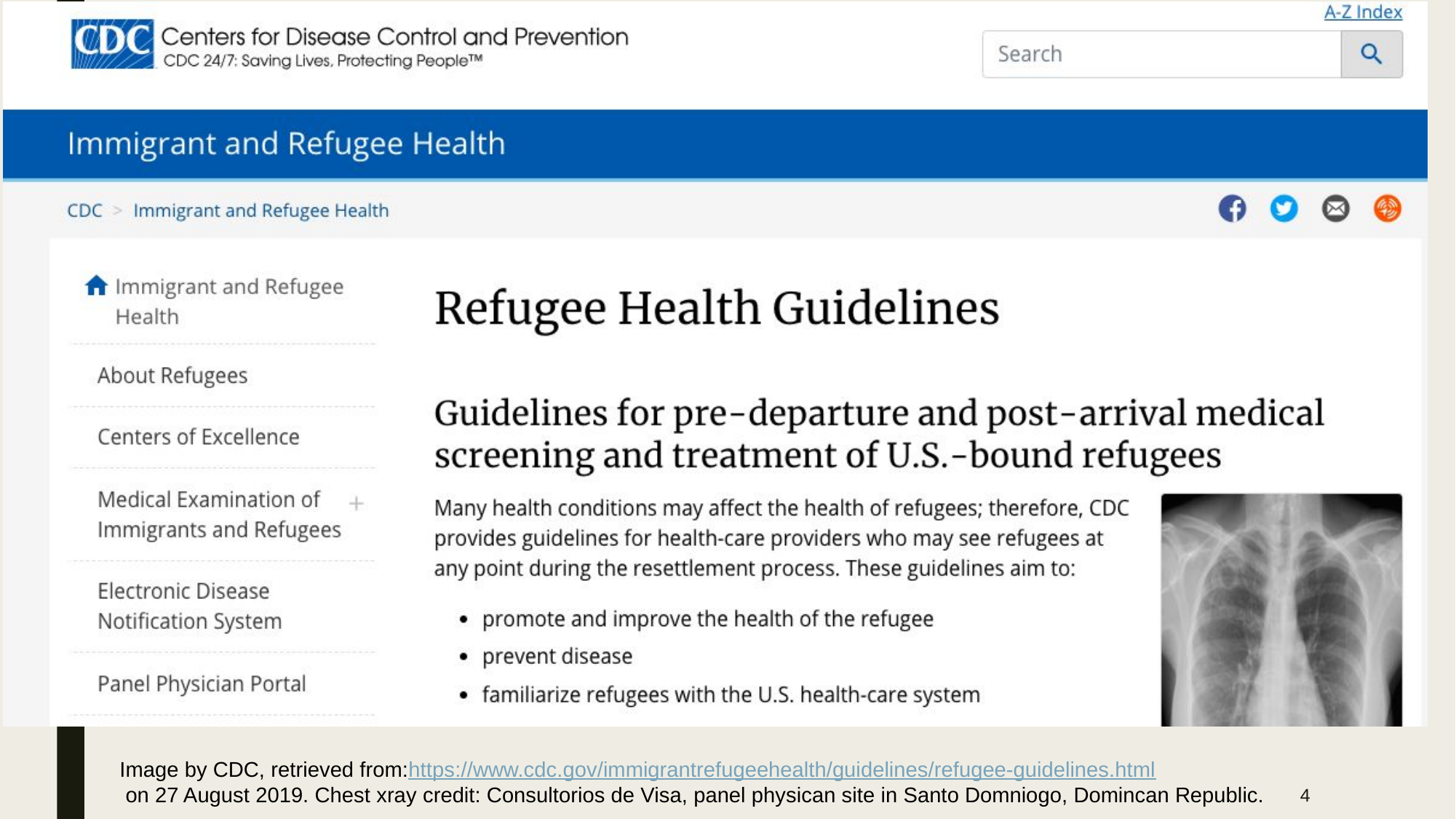

Image by CDC, retrieved from:https://www.cdc.gov/immigrantrefugeehealth/guidelines/refugee-guidelines.html
 on 27 August 2019. Chest xray credit: Consultorios de Visa, panel physican site in Santo Domniogo, Domincan Republic.
4

## Slide 5
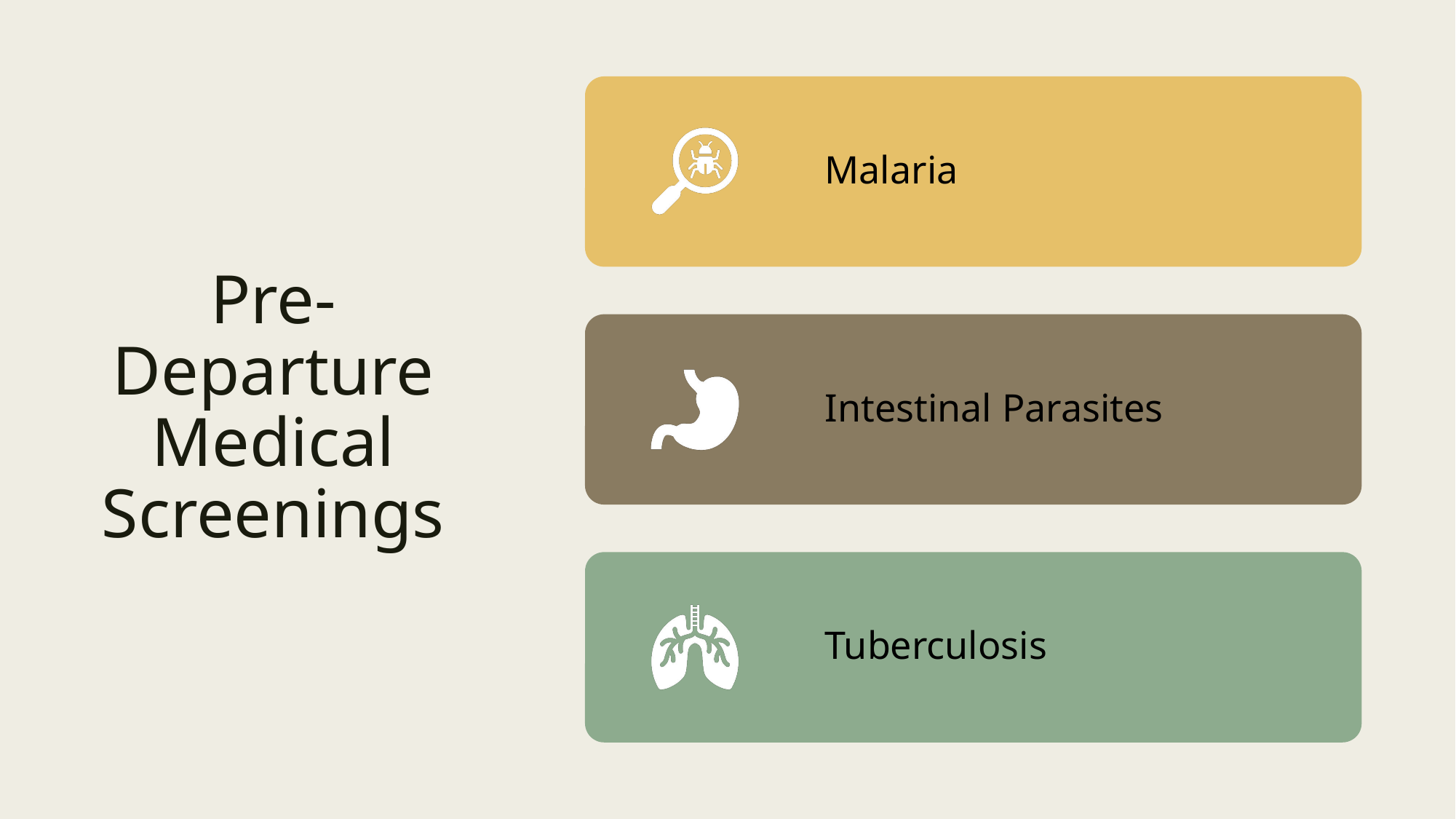

# Pre-Departure Medical Screenings

## Slide 6
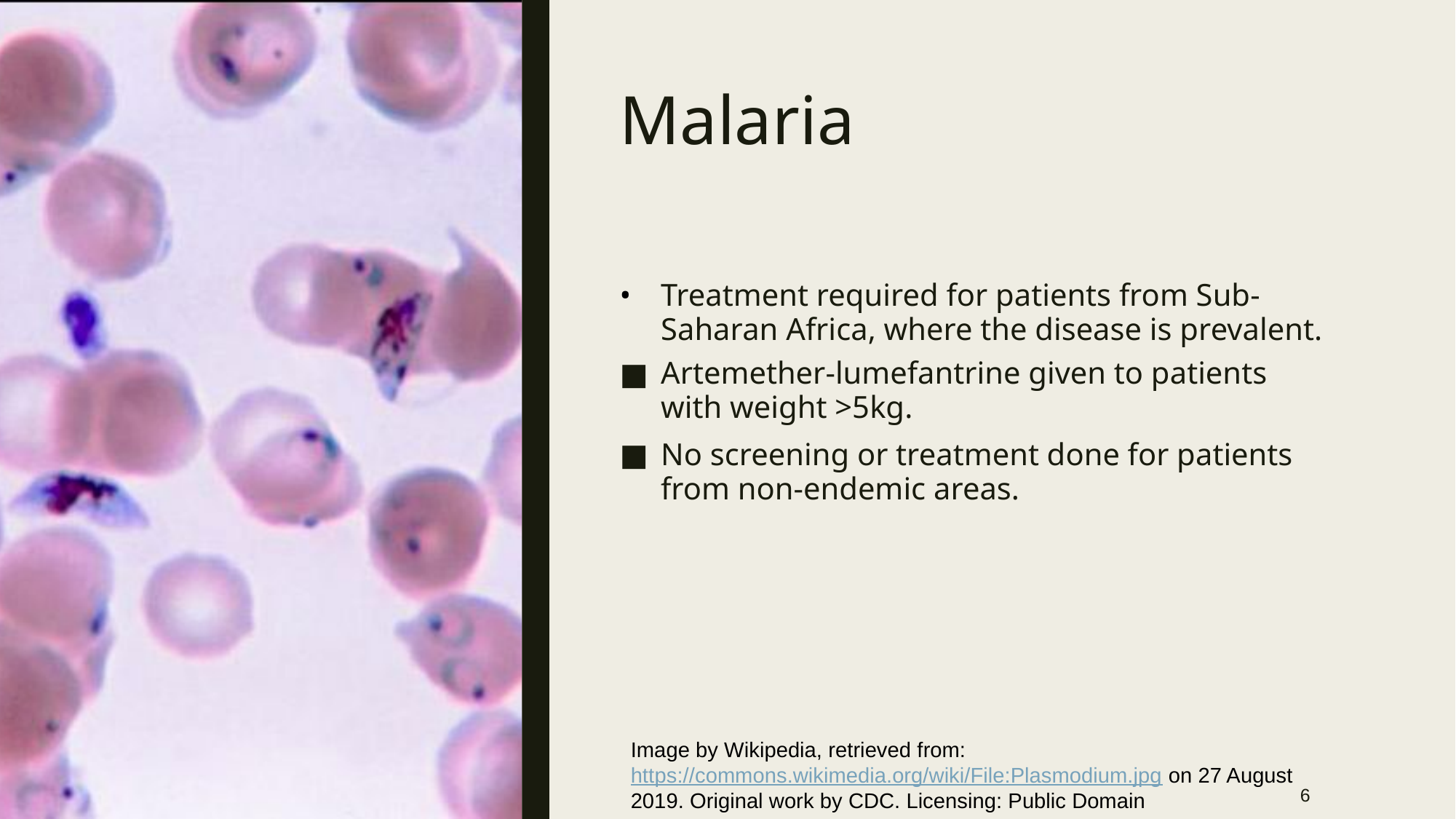

# Malaria
Treatment required for patients from Sub-Saharan Africa, where the disease is prevalent.
Artemether-lumefantrine given to patients with weight >5kg.
No screening or treatment done for patients from non-endemic areas.
Image by Wikipedia, retrieved from: https://commons.wikimedia.org/wiki/File:Plasmodium.jpg on 27 August 2019. Original work by CDC. Licensing: Public Domain
6

## Slide 7
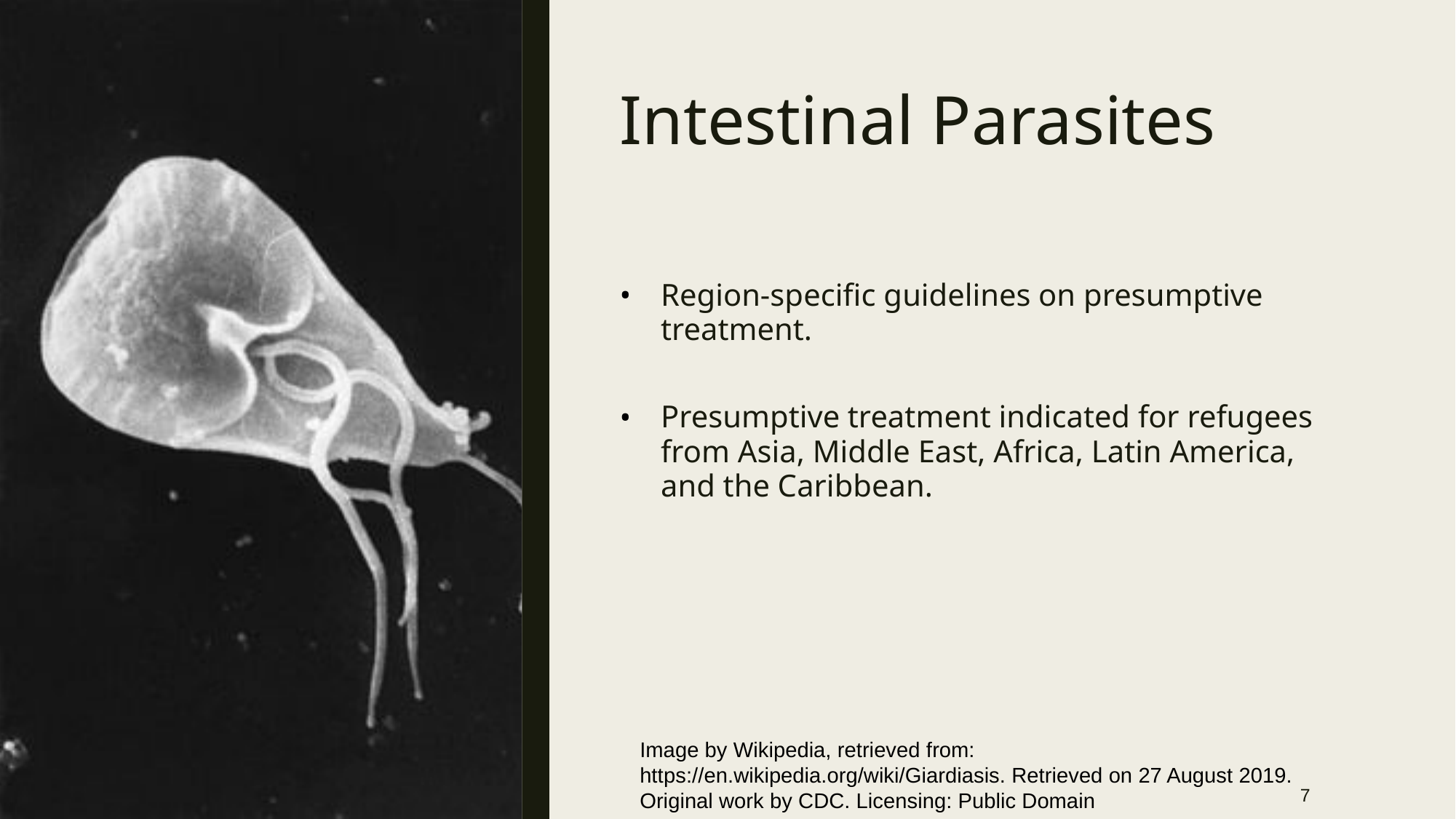

# Intestinal Parasites
Region-specific guidelines on presumptive treatment.
Presumptive treatment indicated for refugees from Asia, Middle East, Africa, Latin America, and the Caribbean.
Image by Wikipedia, retrieved from: https://en.wikipedia.org/wiki/Giardiasis. Retrieved on 27 August 2019. Original work by CDC. Licensing: Public Domain
7

## Slide 8
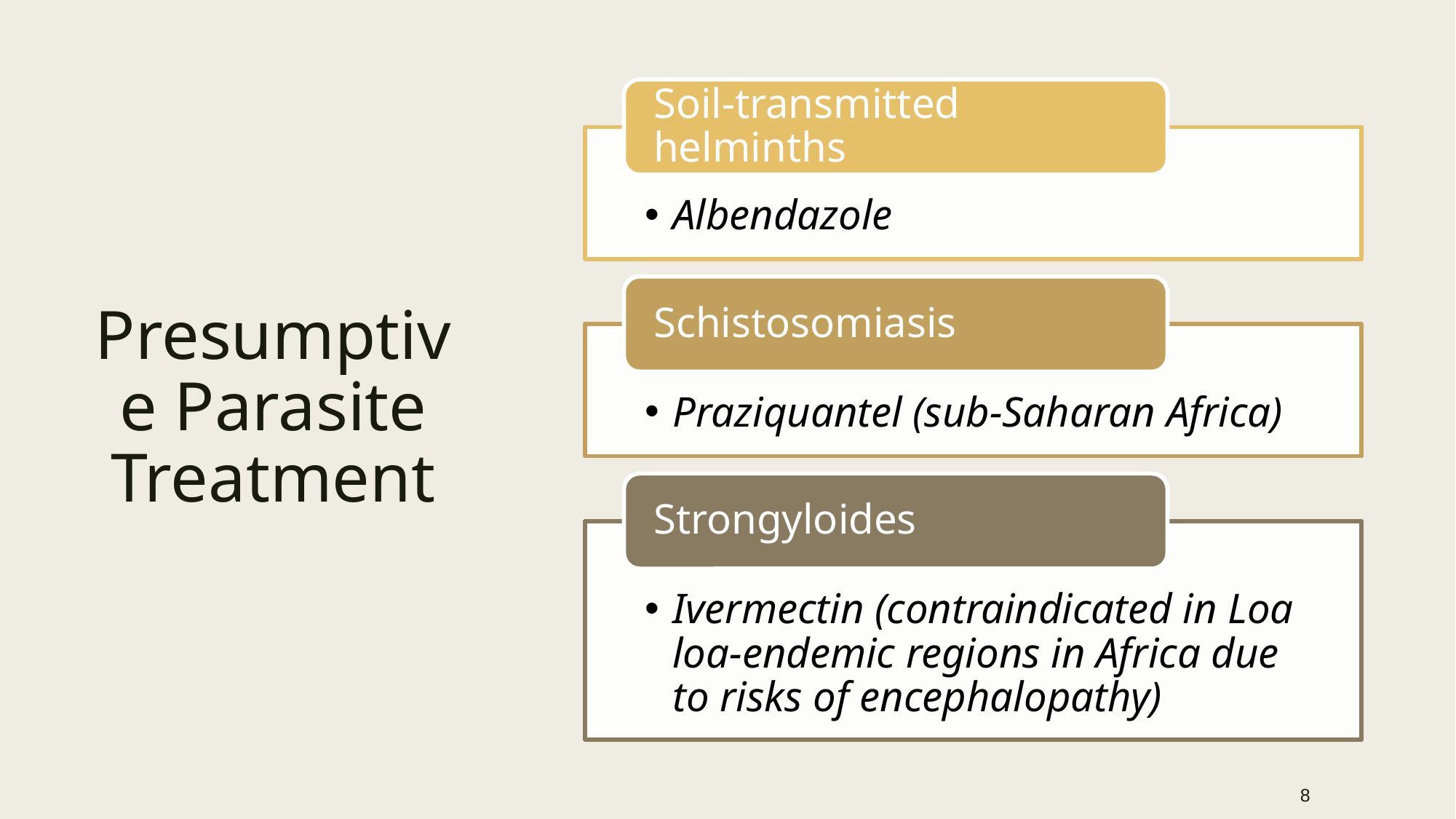

# Presumptive Parasite Treatment
8

## Slide 9
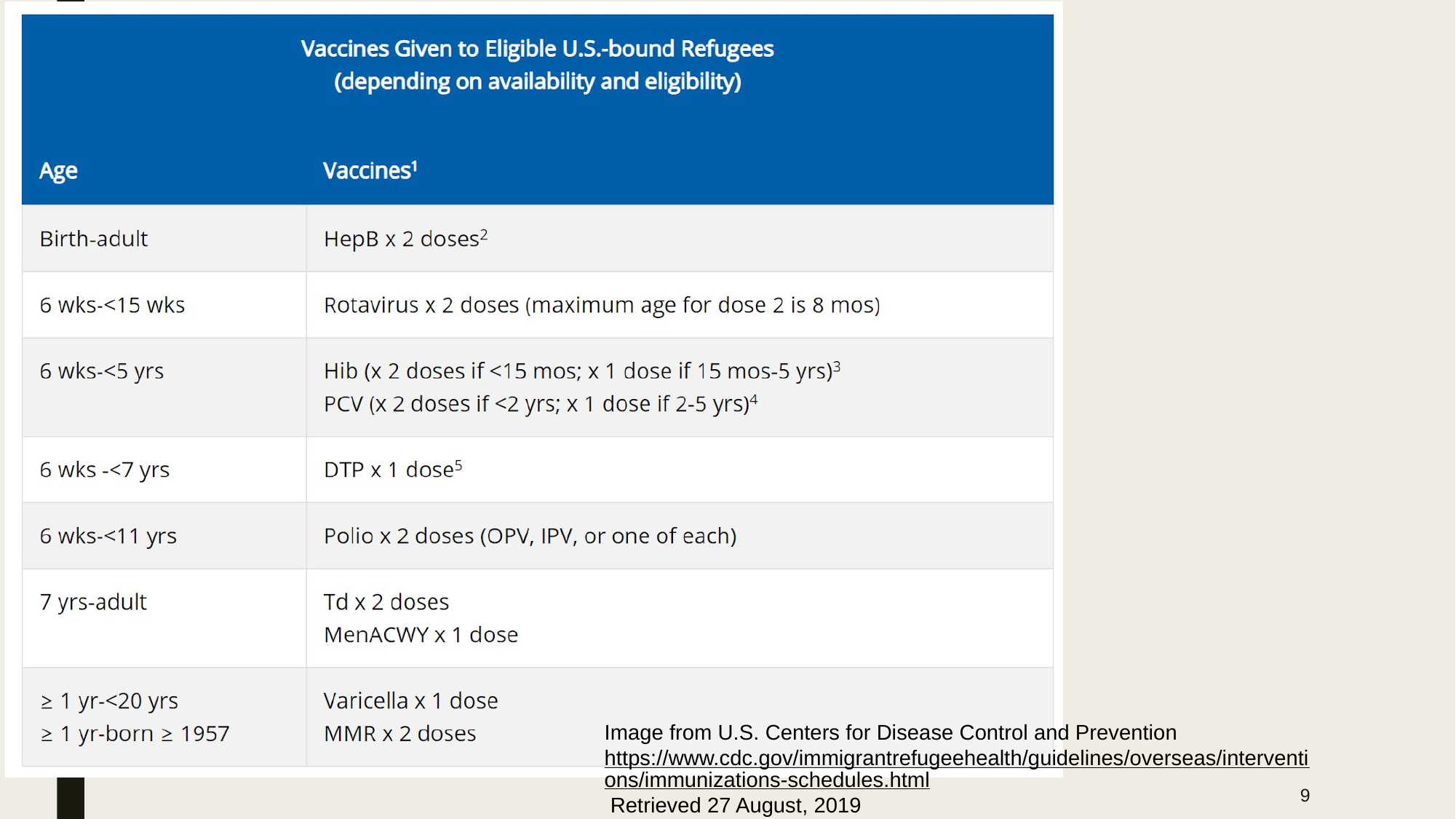

Image from U.S. Centers for Disease Control and Prevention
https://www.cdc.gov/immigrantrefugeehealth/guidelines/overseas/interventions/immunizations-schedules.html Retrieved 27 August, 2019
9

## Slide 10
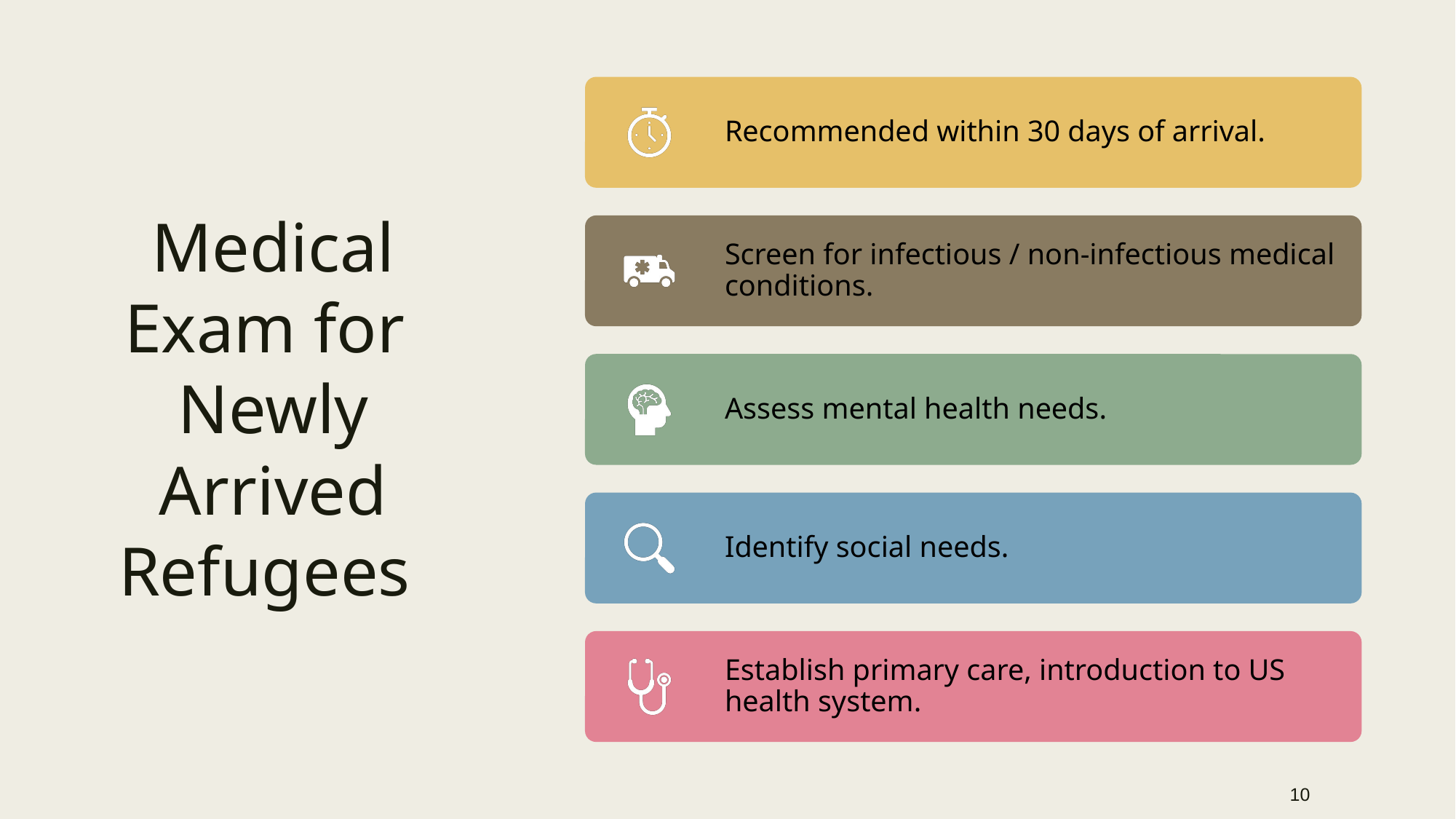

# Medical Exam for Newly Arrived Refugees
10

## Slide 11
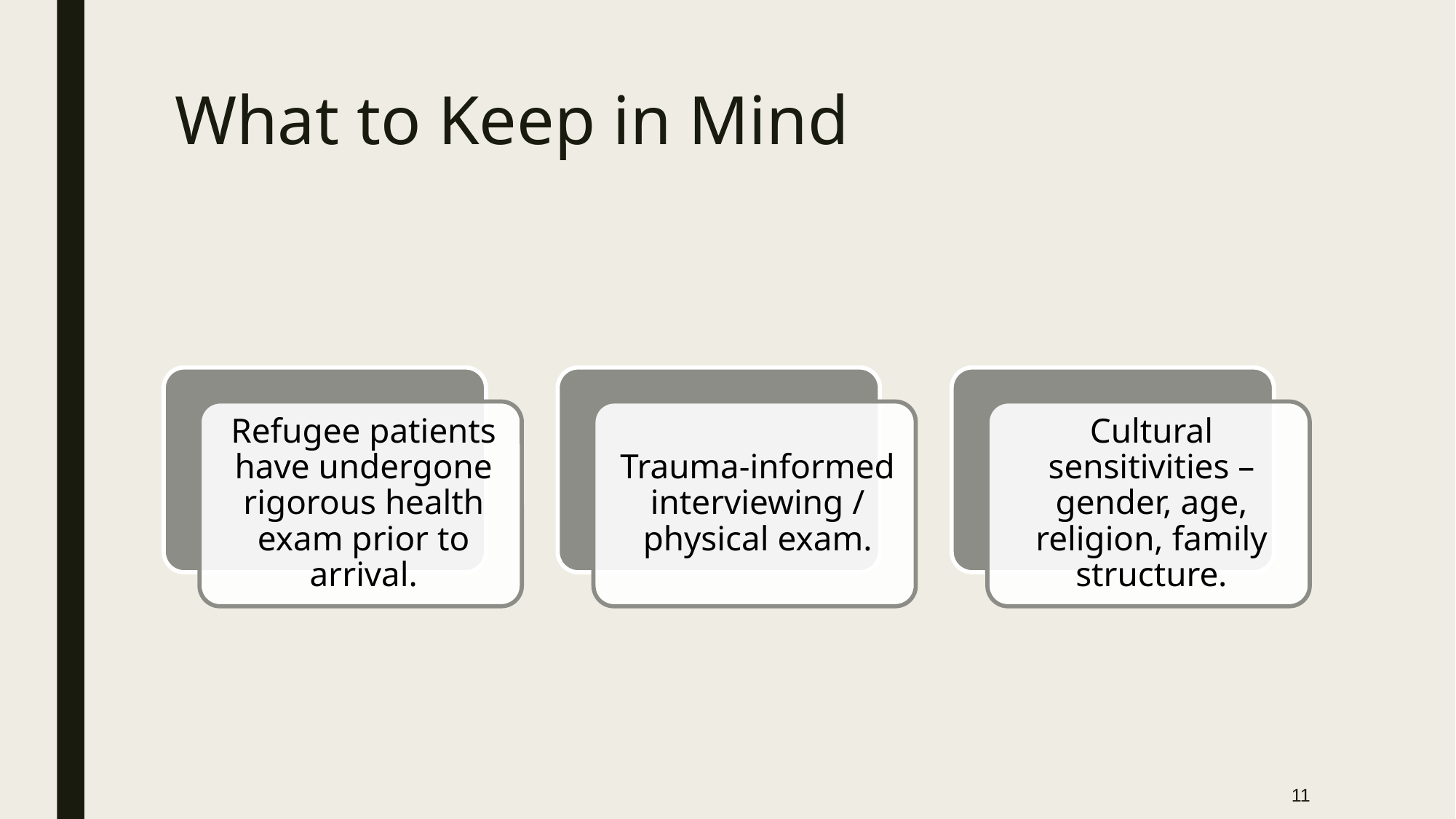

# What to Keep in Mind
11

## Slide 12
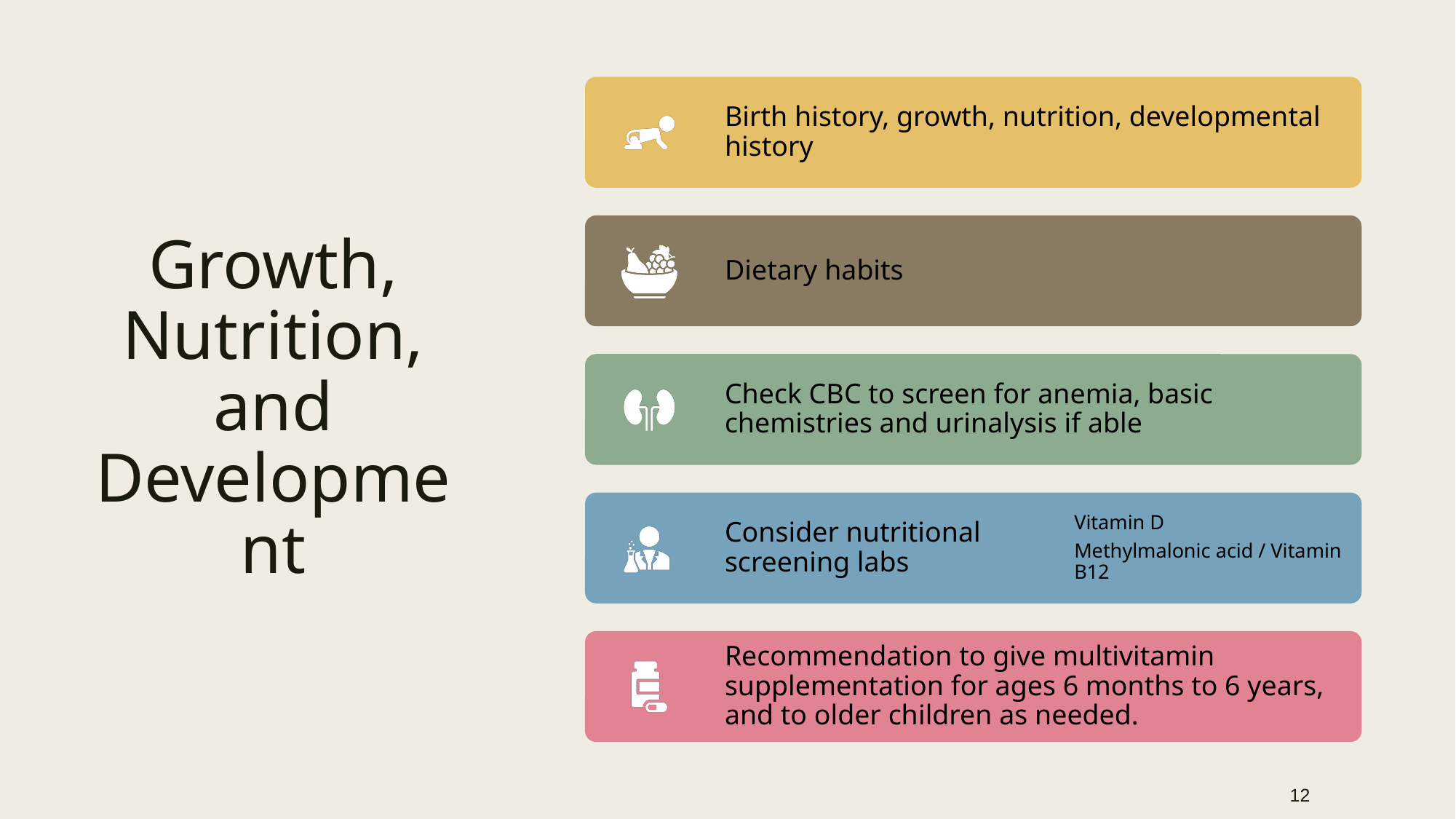

# Growth, Nutrition, and Development
12

## Slide 13
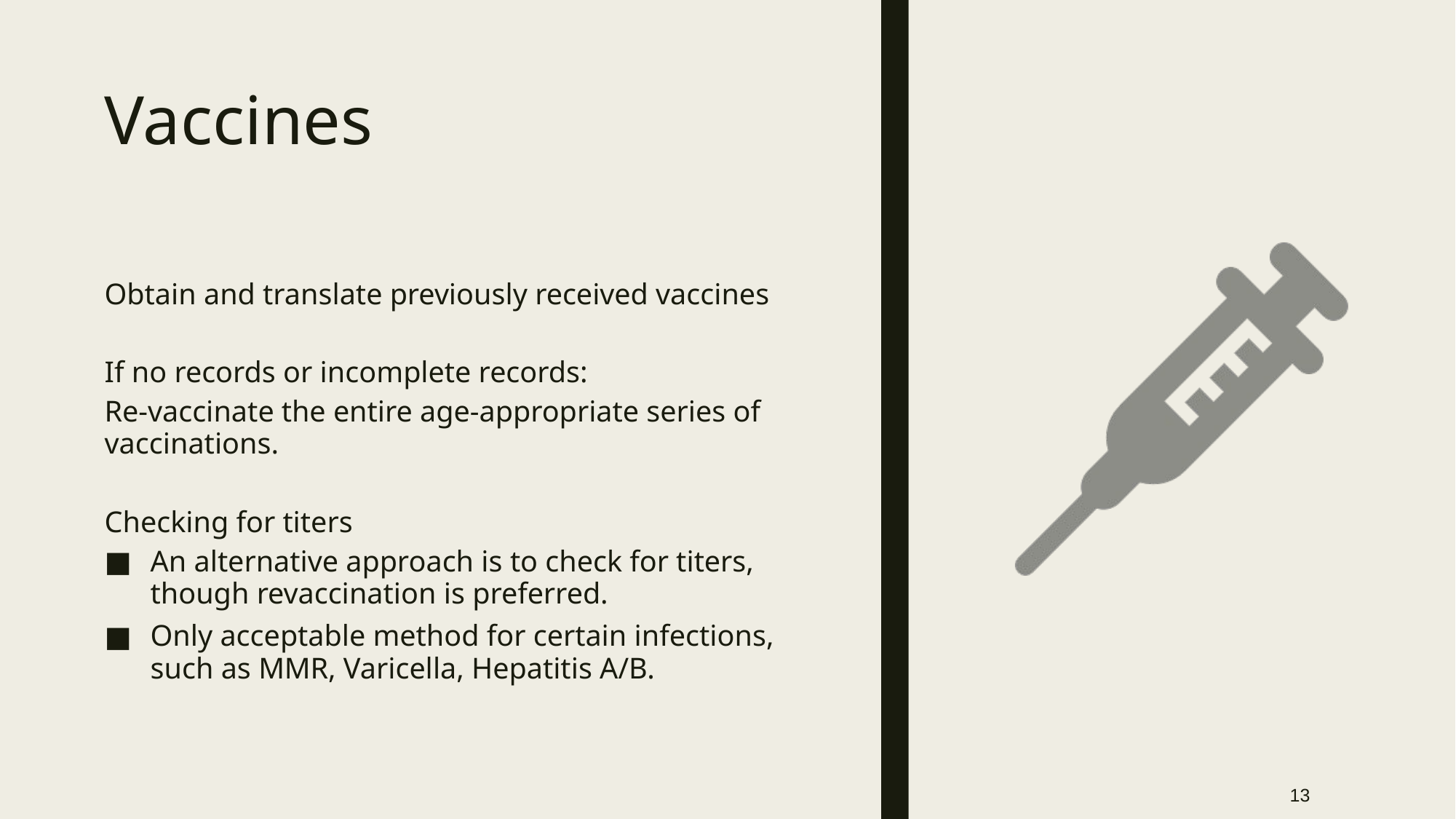

# Vaccines
Obtain and translate previously received vaccines
If no records or incomplete records:
Re-vaccinate the entire age-appropriate series of vaccinations.
Checking for titers
An alternative approach is to check for titers, though revaccination is preferred.
Only acceptable method for certain infections, such as MMR, Varicella, Hepatitis A/B.
13

## Slide 14
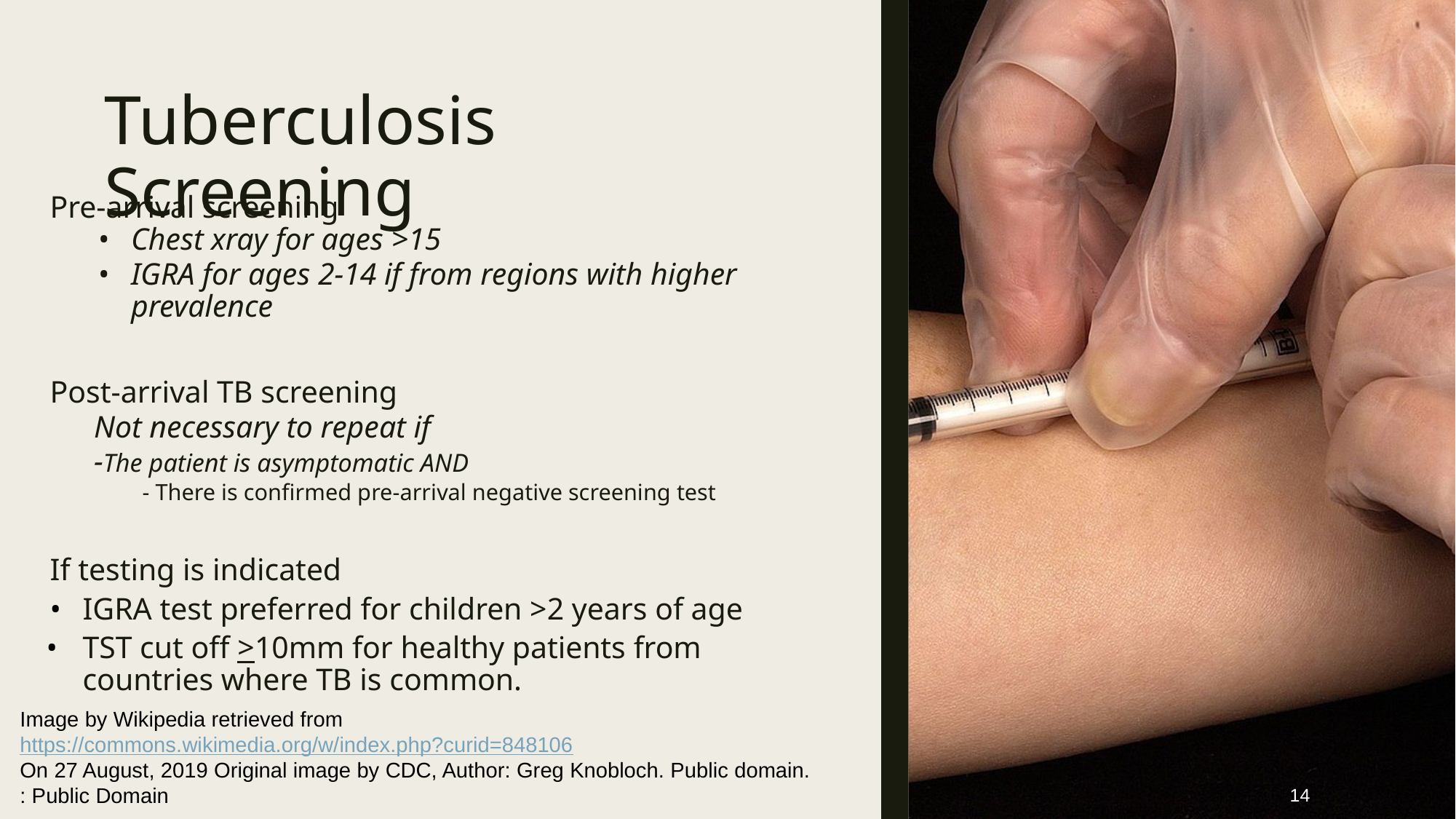

# Tuberculosis Screening
Pre-arrival screening
Chest xray for ages >15
IGRA for ages 2-14 if from regions with higher prevalence
Post-arrival TB screening
Not necessary to repeat if
	-The patient is asymptomatic AND
- There is confirmed pre-arrival negative screening test
If testing is indicated
IGRA test preferred for children >2 years of age
TST cut off >10mm for healthy patients from countries where TB is common.
Image by Wikipedia retrieved from https://commons.wikimedia.org/w/index.php?curid=848106
On 27 August, 2019 Original image by CDC, Author: Greg Knobloch. Public domain.
: Public Domain
14

## Slide 15
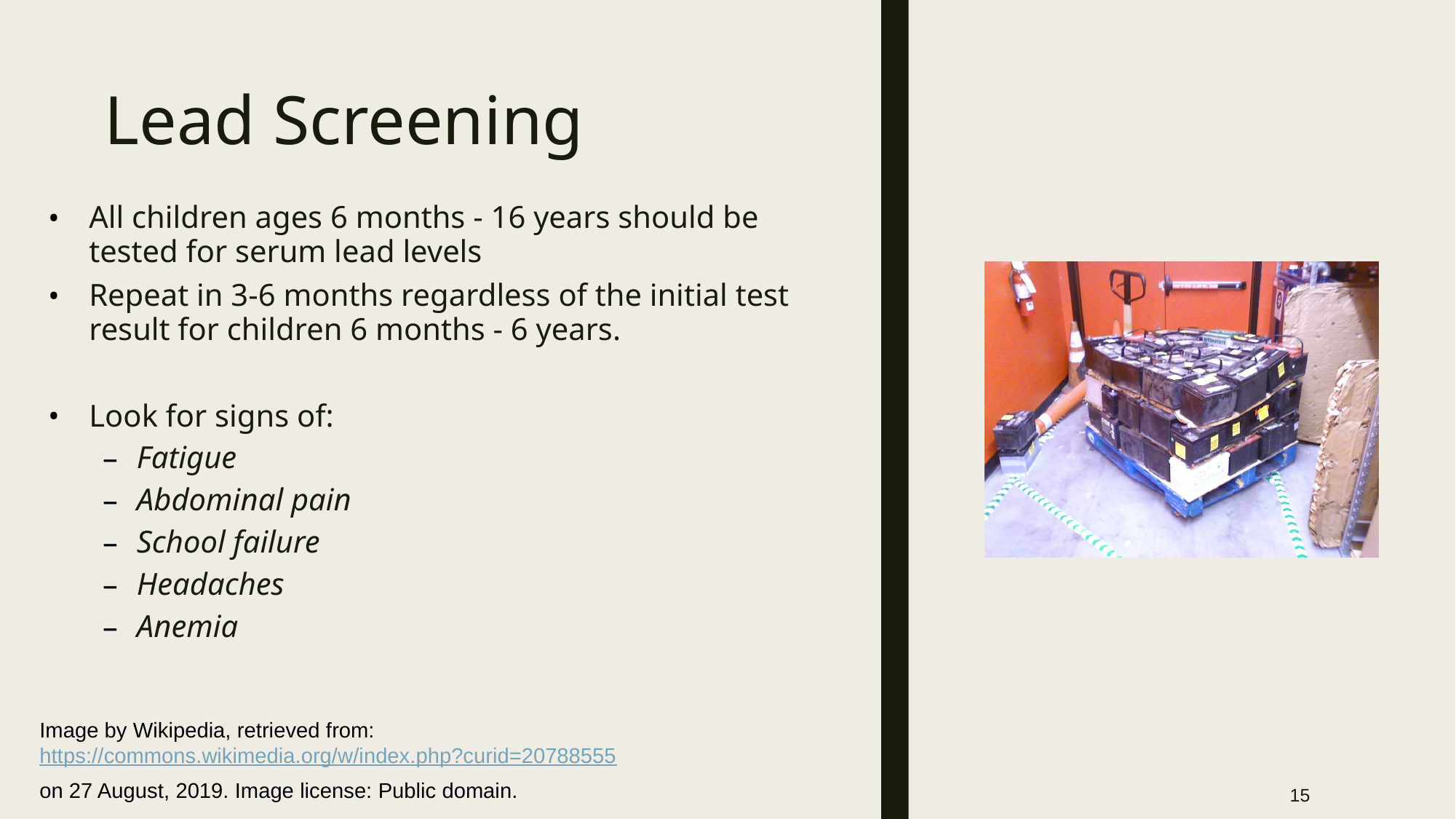

# Lead Screening
All children ages 6 months - 16 years should be tested for serum lead levels
Repeat in 3-6 months regardless of the initial test result for children 6 months - 6 years.
Look for signs of:
Fatigue
Abdominal pain
School failure
Headaches
Anemia
Image by Wikipedia, retrieved from: https://commons.wikimedia.org/w/index.php?curid=20788555
on 27 August, 2019. Image license: Public domain.
15

## Slide 16
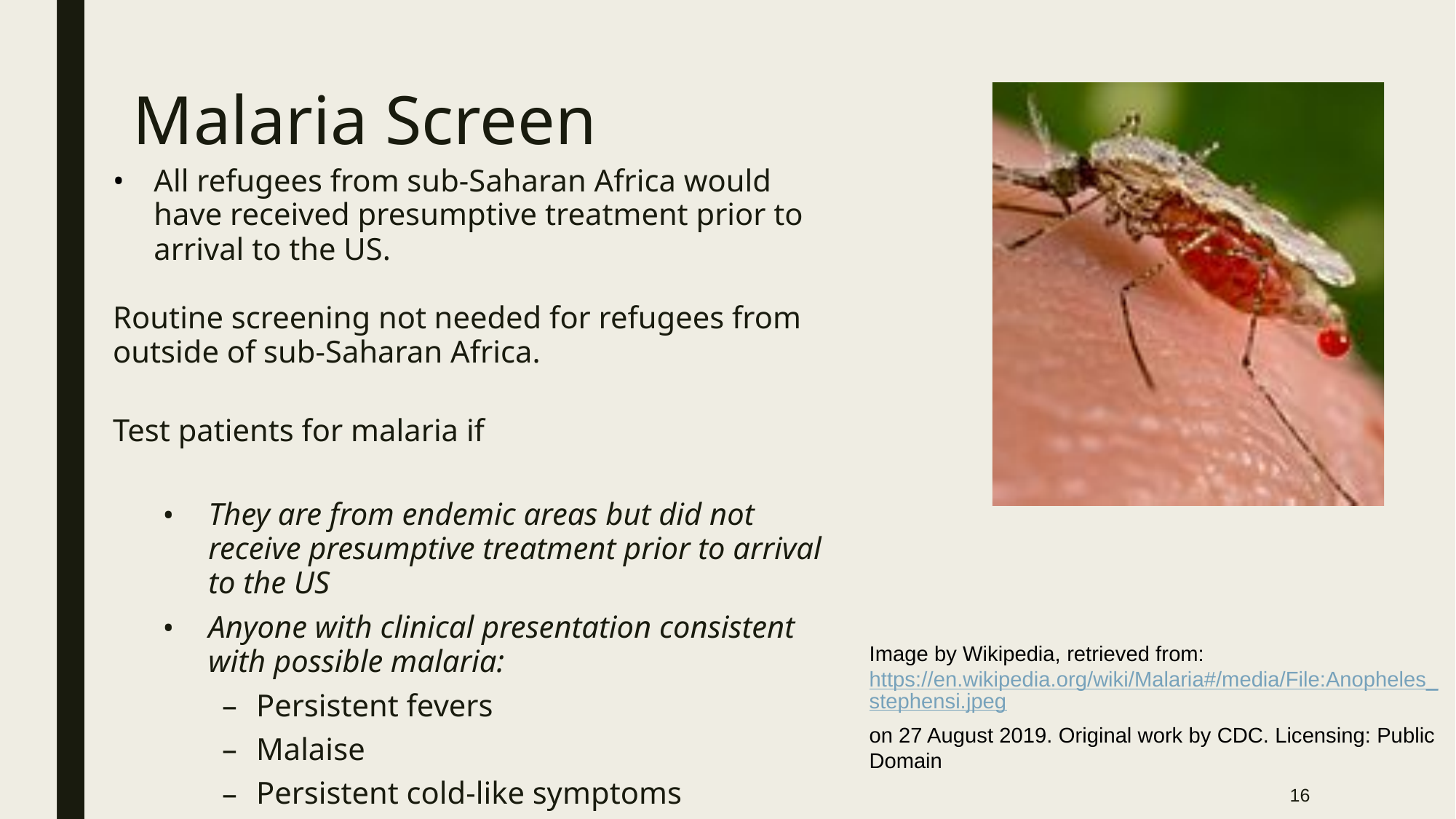

# Malaria Screen
All refugees from sub-Saharan Africa would have received presumptive treatment prior to arrival to the US.
Routine screening not needed for refugees from outside of sub-Saharan Africa.
Test patients for malaria if
They are from endemic areas but did not receive presumptive treatment prior to arrival to the US
Anyone with clinical presentation consistent with possible malaria:
Persistent fevers
Malaise
Persistent cold-like symptoms
Image by Wikipedia, retrieved from: https://en.wikipedia.org/wiki/Malaria#/media/File:Anopheles_stephensi.jpeg
on 27 August 2019. Original work by CDC. Licensing: Public Domain
16

## Slide 17
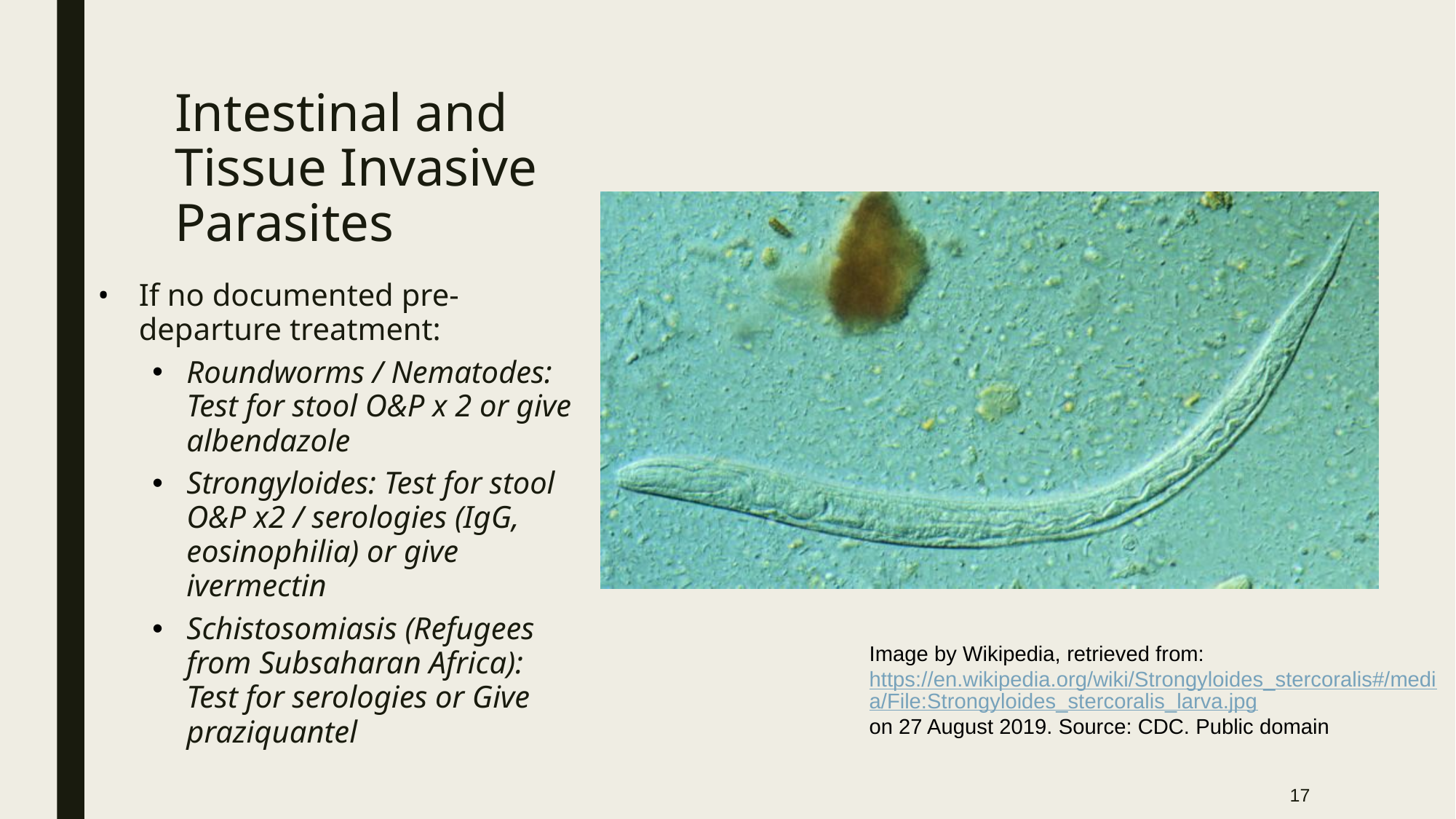

# Intestinal and Tissue Invasive Parasites
If no documented pre-departure treatment:
Roundworms / Nematodes: Test for stool O&P x 2 or give albendazole
Strongyloides: Test for stool O&P x2 / serologies (IgG, eosinophilia) or give ivermectin
Schistosomiasis (Refugees from Subsaharan Africa): Test for serologies or Give praziquantel
Image by Wikipedia, retrieved from:
https://en.wikipedia.org/wiki/Strongyloides_stercoralis#/media/File:Strongyloides_stercoralis_larva.jpg
on 27 August 2019. Source: CDC. Public domain
17

## Slide 18
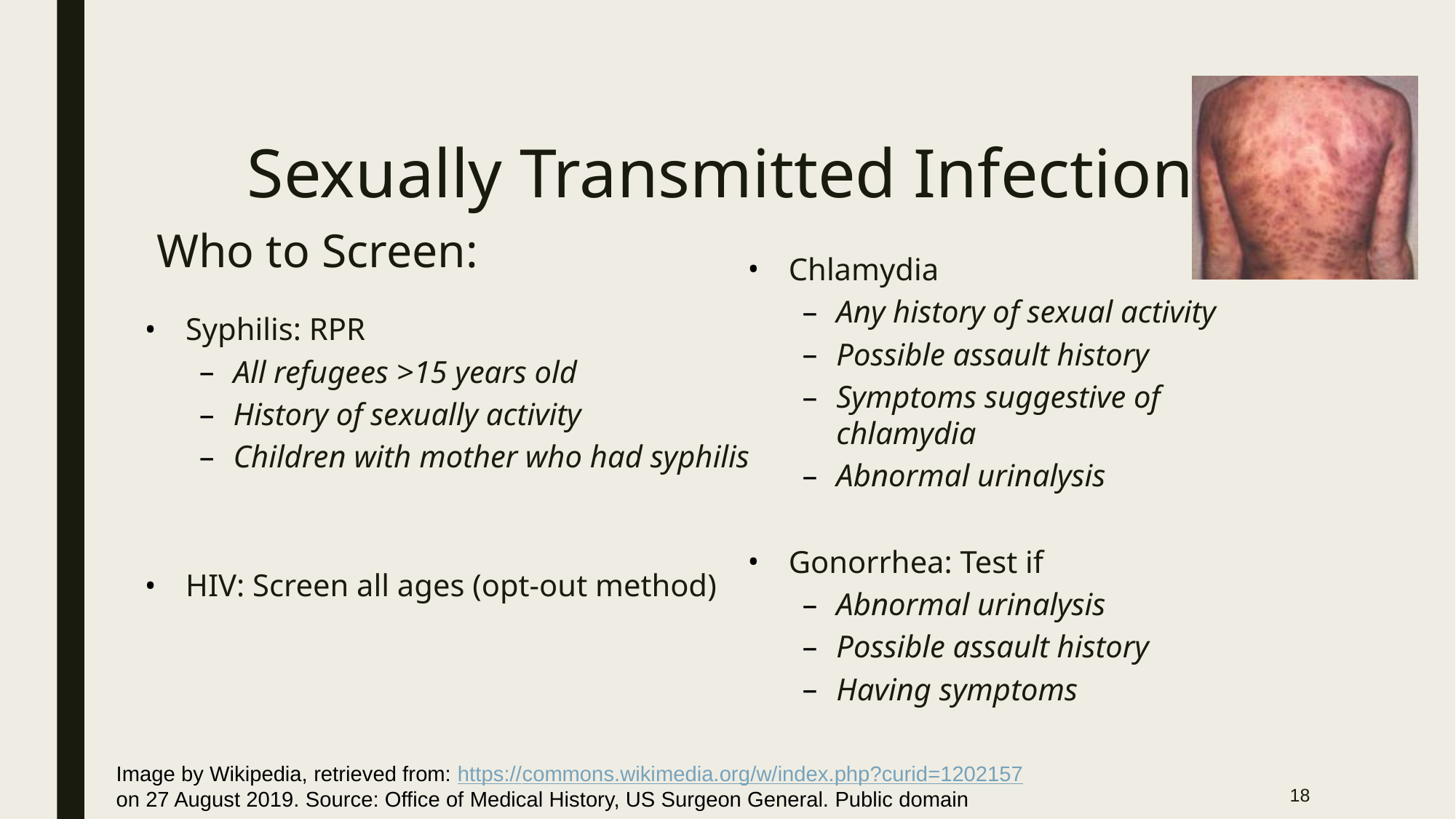

# Sexually Transmitted Infections
Who to Screen:
Chlamydia
Any history of sexual activity
Possible assault history
Symptoms suggestive of chlamydia
Abnormal urinalysis
Gonorrhea: Test if
Abnormal urinalysis
Possible assault history
Having symptoms
Syphilis: RPR
All refugees >15 years old
History of sexually activity
Children with mother who had syphilis
HIV: Screen all ages (opt-out method)
Image by Wikipedia, retrieved from: https://commons.wikimedia.org/w/index.php?curid=1202157
on 27 August 2019. Source: Office of Medical History, US Surgeon General. Public domain
18

## Slide 19
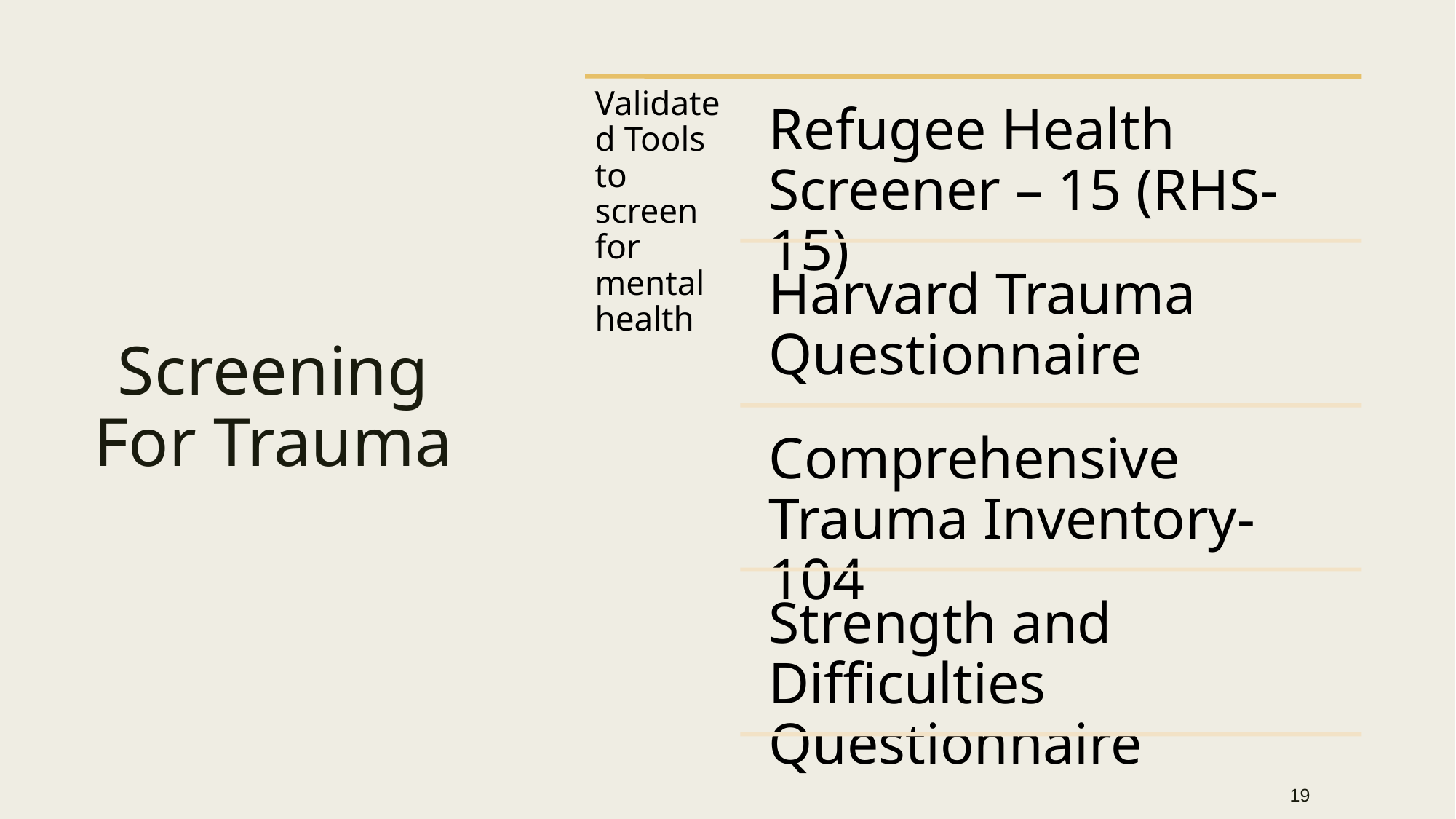

# Screening For Trauma
19

## Slide 20
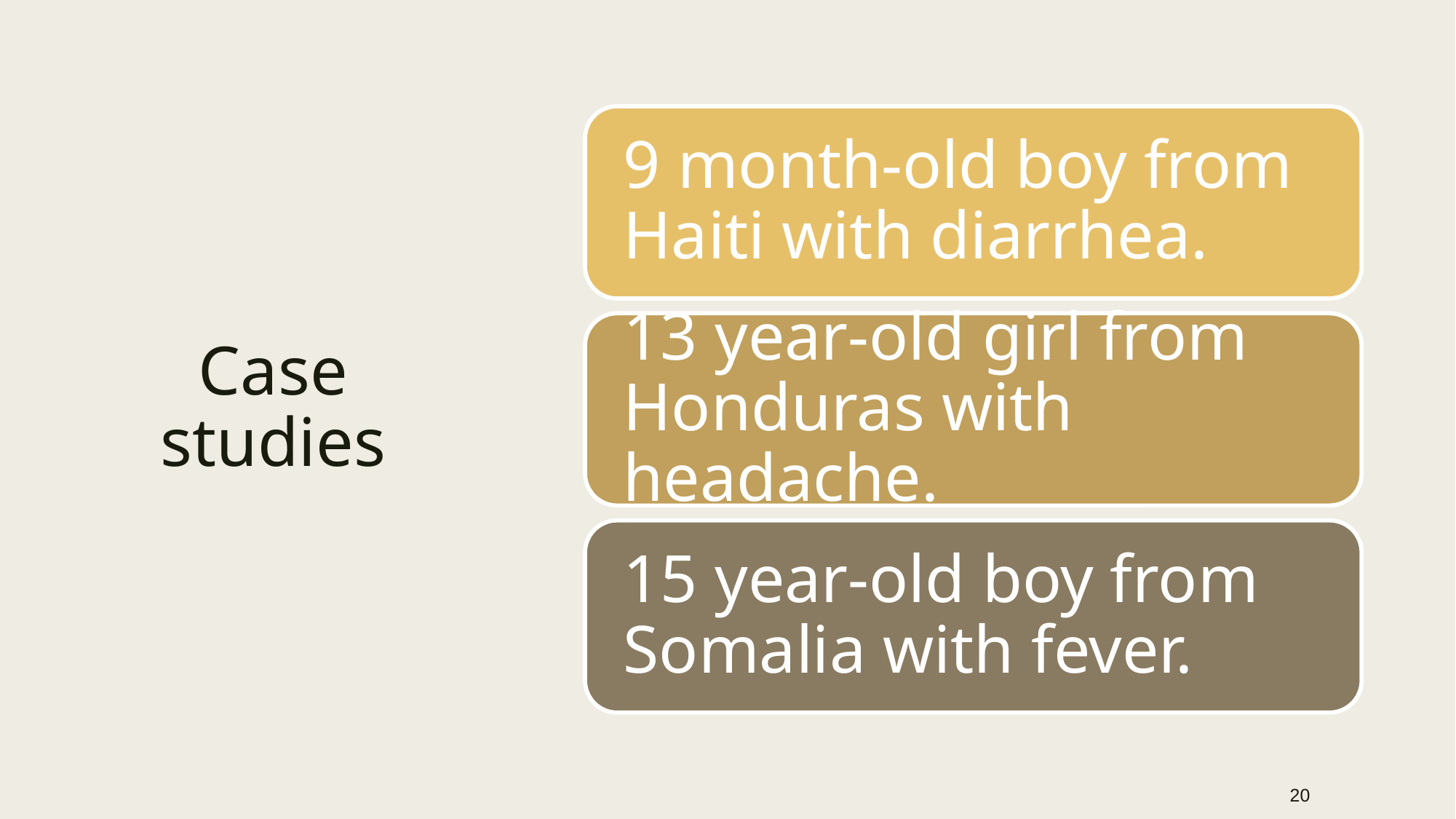

# Case studies
20
