# Supplementary material for: Pediatric Refugee Health Care Delivery in the Community Setting: An Educational Workshop for Multidisciplinary Family-Centered Care During Resettlement
Source: MedEdPORTAL. 2020 Nov 3;16:10988. doi: 10.15766/mep_2374-8265.10988 (PMC7666829; doi:10.15766/mep_2374-8265.10988)
Supplement: Supplementary file 1 — Agenda.docxPresentation 1 Intro to Refugees.pptxPresentation 2 Health Screening.pptxCases.docxPresentation 3 Trauma-Informed Care.pptxPresentation 4 Refugee Health Advocacy.pptxRefugee Workshop Evaluation.docx [file mep_2374-8265.10988-s001.zip › D. Cases.docx]

**Appendix D. Patient Cases**

Domains of cases

|  | **Country** | **Age** | **Pre-arrival experiences** | **Behavioral health issues** | **Medical issues** | **Cultural considerations** | **Additional issues** |
| --- | --- | --- | --- | --- | --- | --- | --- |
| **Case 1** | Honduras | 13 years | Exposure to violence, lack of agency | Suicidal ideation | Psychosomatic symptoms | Trauma-informed exam, shared decision making | Sexual abuse, Pregnancy, STIs |
| **Case 2** | Somalia | 15 years | Exposure to violence, trauma | PTSD, insomnia | Malaria, dengue, anemia, TB, parasites, hepatitis, measles, etc. | Trauma-informed exam, tribal healing practices,  language barriers | Vaccine hesitancy |
| **Case 3** | Haiti | 9 months | Pre-term birth, exposure to pathogens, maternal stressors | Maternal mental health concerns in Failure To Thrive (FTT) | Giardia, strongyloidiasis, schistosomiasis, shigella | Family unit, restavèk culture |  |

**Honduras Case Study**


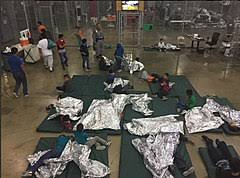


**Example of a detention facility in McAllen, Texas. Image from U.S. Customs and Border Protection retrieved from https://commons.wikimedia.org/wiki/File:Ursula_(detention_center)_2.jpg on June 24, 2019. Image is in the public domain.

**History of Present Illness**: Maria is a 13-year-old female from Honduras with no known past medical history, who presents to the clinic accompanied by a caseworker from a local non-profit organization. She entered the U.S. with her father in Arizona. She and her father were separated after they were apprehended by U.S. Customs and Border Protection. Her father has since been detained by U.S. Immigration and Customs Enforcement. Maria was transferred to the Office of Refugee Resettlement and was placed in a program run by a local non-profit.

Maria has recently begun school, but she often erupts into tears. She has shared that since being separated from her father, she wants to die. She became inconsolable, expressing that she could not breathe, that her head hurt, and her “heart hurt.” She vomited on several occasions. She denies cold symptoms, abdominal pain, or diarrhea. She is unsure of any family history of medical conditions, although she notes she was previously healthy in Honduras.

**U.S. Customs and Border Protection of the Department of Homeland Security**: Includes U.S. Border Patrol responsible for admissions at ports, and the Office of Field Operations responsible for ports of entry.

**U.S. Immigration and Customs Enforcement**: Branch of U.S. Department of Homeland Security that is responsible for investigative and enforcement activity.

**U.S. Department of Health and Human Services Office of Refugee Resettlement**: Responsible for the care and custody of unaccompanied children.

**Medications**: None.

**Vaccinations**: Up to date.

**Allergies**: No known drug allergies.

**Physical Exam**:

Respiratory: Lungs clear to auscultation. No wheezing, rales, or rhonchi.

Cardiac: No murmurs.

Abdomen: Soft, non-tender. Normoactive bowel sounds.

**Vital Signs**:

Temperature: 36.4 C/97.5 F

Blood pressure: 95/70 mmHg

Pulse: 74/min

Respiratory Rate: 16/min

**Labs**: Within normal limits.

**Growth chart**:


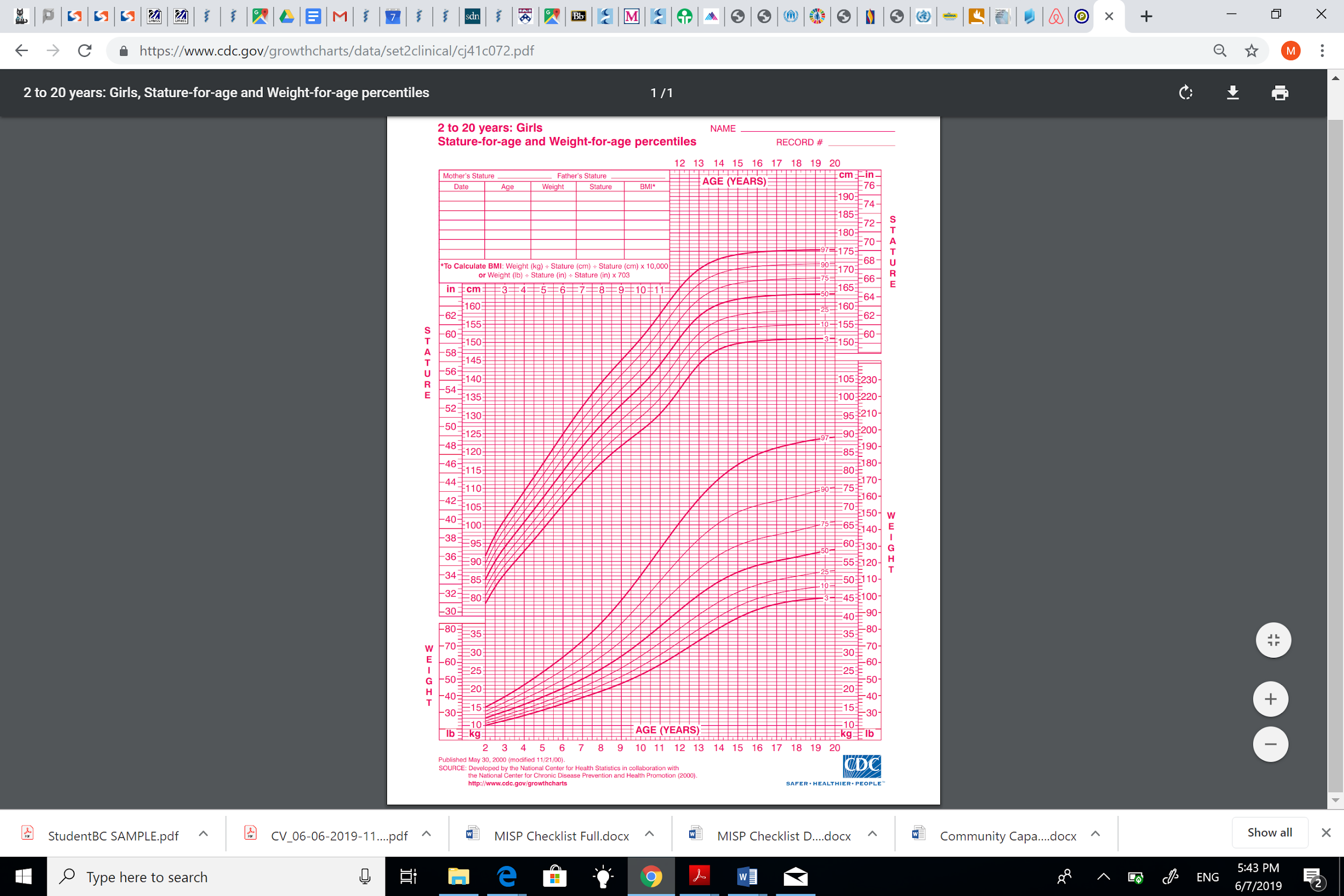



Questions for discussion:

- What health issues are likely present for an adolescent girl such as Maria?
- If Maria were to be relocated to your city or town, what barriers would you need to address to ensure she is able to receive appropriate care? What resources would you need to be able to do this?
- What capacities do you think Maria has to be able to overcome her current challenges?

Narrative adapted from Kids in Need of Defense, Lutheran Immigration and Refugee Service, and Women’s Refugee Commission. [Betraying family values: How Immigration Policy at the United States Border is Separating Families](https://www.womensrefugeecommission.org/rights/resources/1450-betraying-family-values). Washington, DC, 2017.

**Somalia Case Study**



**History of Present Illness**: Abdul is a 15-year-old Somali male who presents with fever. He is accompanied by his mother. He and his mother, as well as four siblings, arrived from Uganda three weeks ago as they were granted resettlement. Abdul’s mother explains that her son has been sick for two years. A Somali leader in his village diagnosed him as having yellow fever, and he had used a hot object for burning the disease. Abdul talked to himself, especially about the killing of his brother and grandmother. He cried a lot. The family therefore left Somalia for Kenya, but due to continuous threats by the Al-Shabaab and Abdul’s worsening condition, they fled to Uganda. They first joined a refugee camp in Uganda (Nakivale), but due to tribalism, they left for Kampala, the capital city. In Kampala, Abdul rarely left his home since he feared abuse. He had problems with armed men and had nightmares, which prevented him from sleeping. He was even scared of himself. Fearing potential suicide, his mother attempted to seek help at a local hospital in Kampala. The doctors told her they could not do anything. She felt it was because she and Abdul did not speak the language, lacked money, and were refugees.

This visit, Abdul has been lethargic with fever, nausea, and headache for the past four days. He has had several episodes of vomiting and watery diarrhea each day, and high fevers, chills, myalgia, and drenching sweats every other day. He denies cough, hemoptysis, or hematochezia.

**Medications**: Recently finished albendazole for parasitic treatment that was prophylactically prescribed prior to resettlement. He had not taken malaria prophylaxis since his mother felt it unnecessary, as Abdul was asymptomatic.

**Allergies**: No known drug allergies.

**Vaccines**: On track from domestic medical screening conducted during the first week of arrival.

**Physical Exam**:

Eyes: Jaundice.

Respiratory: Lungs clear to auscultation. No wheezing, rales, or rhonchi.

Cardiac: No murmurs.

Abdomen: Soft, non-tender. Enlarged spleen and liver.

Musculoskeletal: Normal range of motion.

Skin: No petechiae.

**Vital Signs**:

Temperature: 40.2 C /104.4 F

Blood pressure: 92/62 mmHg

Pulse: 130/min

Respiratory Rate: 24/min

**Labs**: Mild anemia, low platelets.

**Growth chart**:


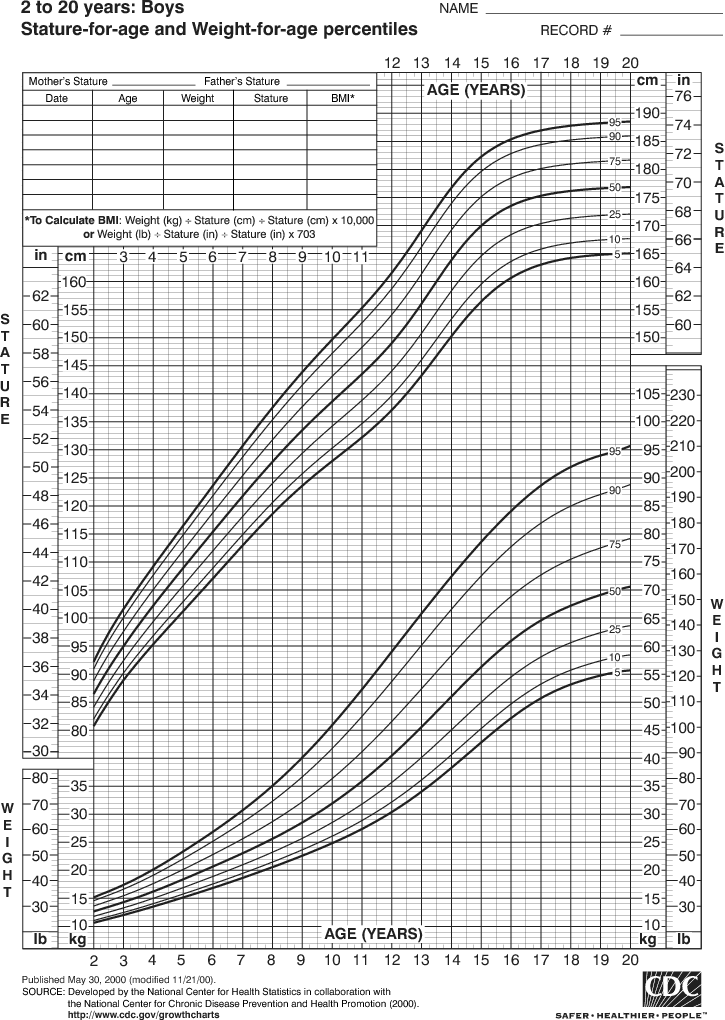



Questions for discussion:

- What health issues are likely present for an adolescent boy such as Abdul?
- If Abdul were to be resettled in your city or town, what barriers would you need to address to ensure he is able to receive adequate care? What resources would you need to be able to do this?
- What capacities do you think Abdul and his family have to overcome their current challenges?
- If Abdul were a Somali adolescent girl, what additional concerns would we like to consider?

Narrative adapted from: Tanabe, Mihoko, Yusrah Nagujjah, Nirmal Rimal, et al. “Intersecting Sexual and Reproductive Health and Disability: Needs, Risks, and Capacities of Refugees with Disabilities in Kenya, Nepal, and Uganda," *Sexuality and Disability* 2015, 33(4):411-427.

**Haiti Case Study**


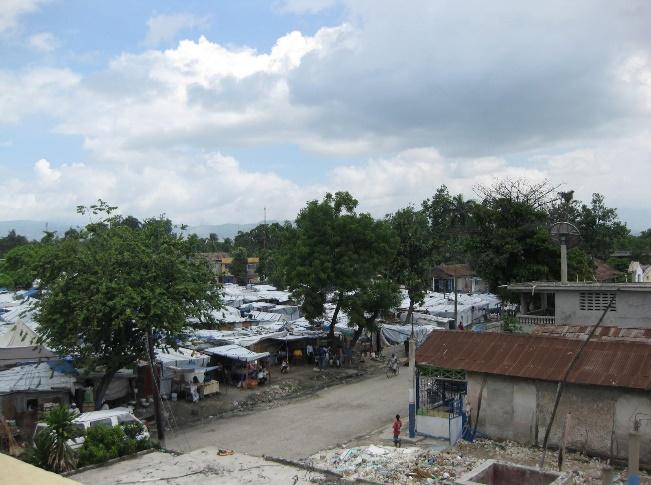

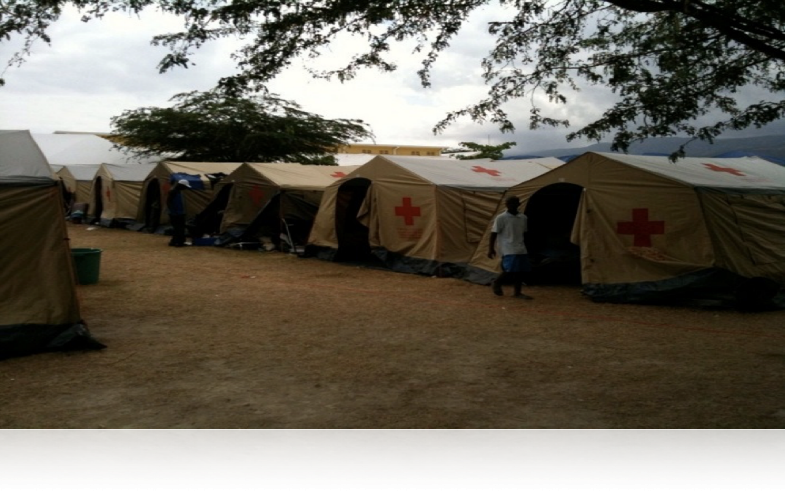


(Photo credit: Author owned.)

**History of Present Illness**: Jean-Louis is a 9 month-old Haitian male with a past medical history of preterm birth and no other complications, who presents to your primary care clinic with his mother, father, and two older siblings for a sick visit. The family had presented for their initial refugee screening some weeks earlier where you learned that the family had emigrated to the US through sponsorship of extended family after their home and livelihood had been destroyed. Prior to emigrating, the family lived for several months in a tent village in Port-au-Prince where resources such as access to food and clean water were limited. In order to support themselves, Jean-Louis’s older brother and sister had to work as domestic servants (*restav*è*ks*) for a family in the city. It was while they were living in the tent village that Jean-Louis was born at 35 weeks gestation.

Jean-Louis presents today with diarrhea for the past 7 days. The family reports that he has had diarrhea off and on since he was born--it will resolve for several weeks and then return. They report his stools sometimes appear greasy. His feeding does diminish during his episodes of diarrhea, and they are concerned that he is not gaining weight the way his siblings did when they were his age. His mother expresses concern that, due to the circumstance of his birth and early life, she was not able to give him the same attention she gave to his siblings. She denies any fever, vomiting, constipation, or blood in his stool.

**Medications**: None.

**Vaccinations**: Up to date.

**Allergies**: No known drug allergies.

**Physical Exam**:

Head: Anterior fontanelle mildly sunken; posterior fontanelle closed. Normal shape and size.

Eyes: No jaundice. Red light reflex present. Normal eye alignment.

Chest/heart: Normal s1 and s2.

Abdomen: Bowel sounds present. Soft, non-tender to palpation. No hernia apparent.

Musculoskeletal: No hip dysplasia.

Neuro: Plantar grasp reflex present.

Skin: Dry to touch; no rash.

**Vital Signs**:

Temperature: 98.2F (36.7C)

Blood pressure: 80/55 mmHg

Pulse: 150/min

Respiratory rate: 25/min

Weight: 18.7 lbs (8.5 kg); lost 2.5 lbs since last visit 2 weeks ago.

**Labs**: Pending


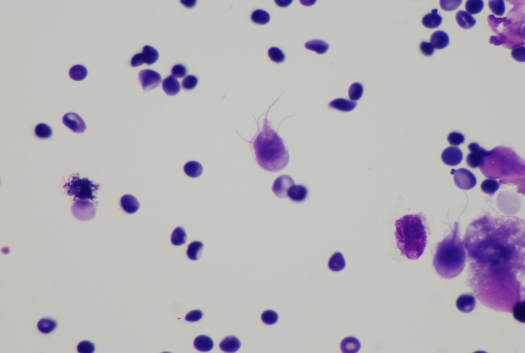
(Photo credit: “Image by Jared M Gardner, MD, retrieved from:

https://commons.wikimedia.org/wiki/File:Giardia_lamblia_cytology.jpg on 8/22/2019. Creative Commons License associated: CC-BY-SA-3.0.)

**Growth Chart:**


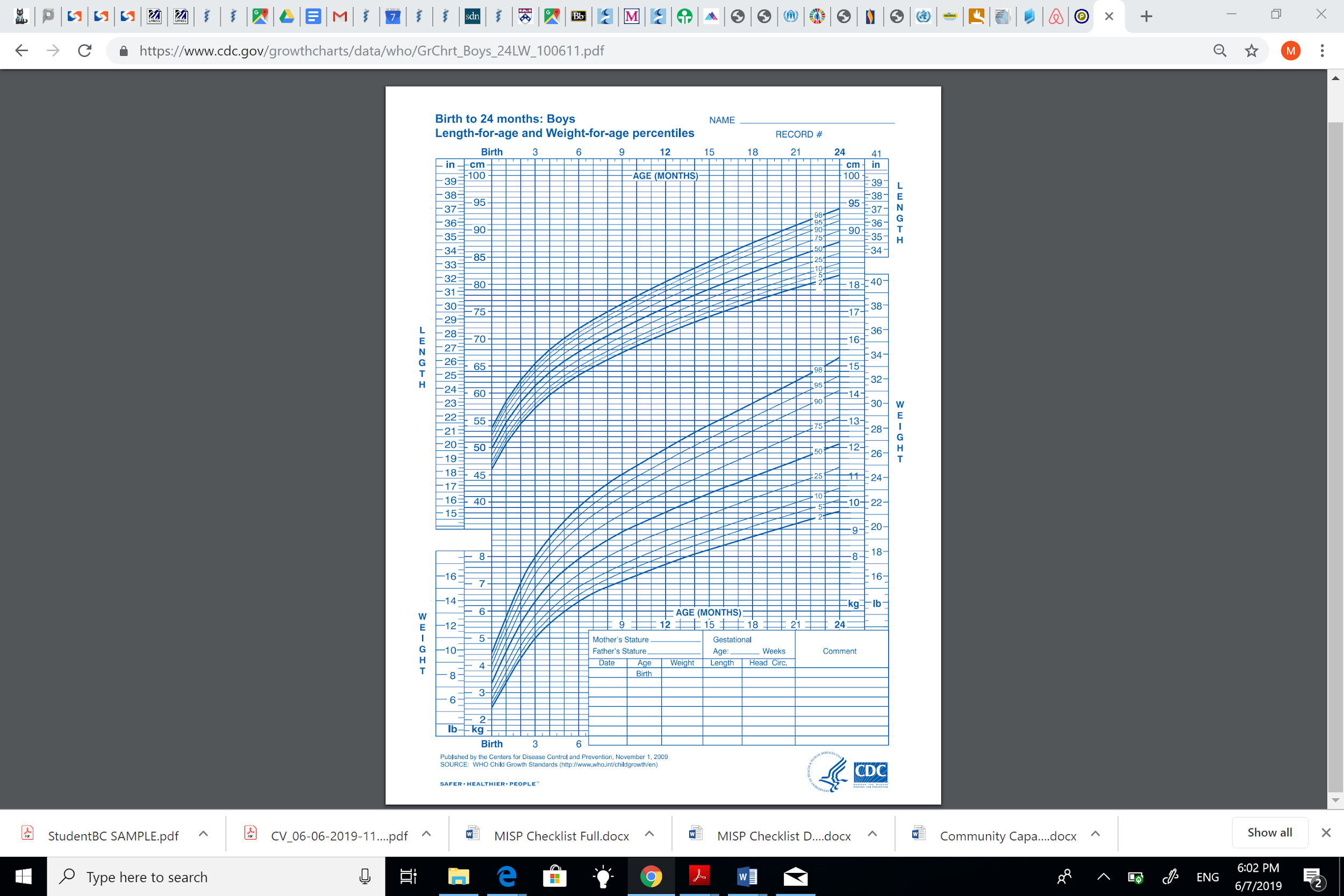



Questions for discussion:

- What are your top 3 differentials for Jean-Louis’s complaint and why? How would you confirm your diagnosis and manage it if correct?
- What are some of the biological risk factors Jean-Louis faced prior to his arrival in the US? What are some of the psychosocial risk factors?
- What are some of his ongoing biopsychosocial risk factors?
- What about current protective factors? What resources might the family be able to access?
- Are there any referrals you would consider making for the patient or family?

**Questions for Discussion**

**Suggested Framework, Approach, and Responses for Facilitators**

**Overall Approach:**

There are many possible educational frameworks for effective teaching. One that is well suited for case discussions is Bloom’s Taxonomy originally described by educational psychologist, Benjamin Bloom. Below is a table based on Bloom’s Taxonomy to offer guidance to facilitators to encourage higher levels of cognition and synthesis during the discussion. The facilitator should support learners to assemble the components from the slide presentations, plus learners’ own knowledge, to formulate plans for family-centered, culturally sensitive, trauma-informed care.

| Level 1 | Remembering | Describe, Tell, List, |
| --- | --- | --- |
| Level 2 | Understanding | Discuss, Outline, Summarize |
| Level 3 | Applying | Categorize, Use, Demonstrate, Employ |
| Level 4 | Analyzing | Illustrate, Interpret, Complete, Contrast, Compare, Connect |
| Level 5 | Evaluating | Infer, Summarize, Prioritize, Rate, Justify, Decide, Recommend |
| Level 6 | Creating | Rearrange, Plan, Develop, Devise, Design, Create, Formulate, Imagine |

This guide is designed for facilitators and participants with any level of experience with refugee health. If the participants are of different levels of training/experience, provider types, clinical settings, or personal backgrounds, then this is an opportunity to promote perspective taking.

The cases are constructed to incorporate multiple domains (physical, mental, cultural, etc) and some ambiguity for learner-centered and learner-driven education. Some suggested prompts are included below. Not all of the prompts below can be covered in the suggested time of 20 minutes. Please customize and guide the discussion according to context and to learner needs:

- Ask participants what data or information are not included in the cases that they may need to elicit.
- Encourage participants to engage the patient or parent in data collection (symptom diaries).
- Encourage discussion in absence of these data as this is often true in actual practice. This is an important part of reducing clinician barriers to care of refugee populations.
- Encourage participants to offer examples from their own experiences, whether from refugee health or from caring for another population.
- Promote finding commonalities with vulnerable populations with which participants may have familiarity. These may include language barriers, housing insecurity, transportation barriers, affordability, social isolation, experience of trauma, bullying, stigma.
- Ask participants to create the ideal care team needed for comprehensive care and/or to compare examples of care teams from their own settings. Where is there a gap between ideal and existing resources? Are there innovative approaches to address gaps?
- Elicit range of potential options for evaluation or treatment.
- Remind of shared decision making with the family when there is more than one reasonable option.
- Elicit ways to promote development of relationships and trust with the patient and family for a step-wise approach to care (multiple visits, who is in the room during the visit, chaperones, etc).
- Support recognition that the parent or other family members may have mental health conditions.
- While cases may describe the obvious trauma of displacement and/or separation, explore for other sources of psychological trauma from relationships or bullying within or outside the home.
- Consider positive and negative roles of social media, news, and social connections on mental health.
- Encourage awareness of historic and current gender biases in healthcare that may cause providers to discount physical symptoms as psychological.
- Encourage awareness that gender norms, gender identity, and sexuality of children or teens can vary not only by culture but by individuals.
- Explore the ambiguity and overlap of medical and psychological symptoms.
- Imagine the experiences of displacement, resettlement, screening, that may cause a patient or family to distrust medical professionals or authorities.
- Offer an opportunity for self reflection on various types of biases or assumptions.
- Consider fears or barriers healthcare personnel may have about accepting a refugee patient or family.
- Consider potential of healthcare personnel or staff having anti-refugee, anti-immigrant, anti-Muslim, xenophobic, or racist attitudes that may add barriers.
- Encourage recognition of diversity within any culture. For example, two Haitian patients may come from different subcultures, socioeconomic backgrounds, religious communities, or family dynamics thus emphasizing the importance of being family-centered.
- Recognize that refugees may have had different social standing or economic stability prior to displacement. Identity loss and grief may occur in many ways.
- Consider the role of faith-based groups as both potential resources for and barriers to medically valid and culturally competent care.
- Elicit suggestions of who may be trusted and respected authorities or influencers for the family.
- Consider the role of local or municipal government, which may have a different position on refugees than state or federal governments.

**Trauma-Informed Teaching and Managing Conflict:**

Recognize the possibility that participants themselves may have personal experience of refugee status, abuse or trauma, PTSD, or other mental health conditions. Be mindful of trauma-informed approaches to teaching.

- Facilitate respectful debate when there is ambiguity or difference of opinion. There may be a need to diffuse tension or to redirect. Helpful phrases include:
  - “*I hear you both saying similar things in different ways*”
  - “*Can you expand on that?*”
  - “*Can you help us understand why you say that?*”
  - “*What information in the case or from your experience supports your perspective on this?*”
  - “*There is no one clear right answer here*”
  - *“These are challenging topics”*
  - *“In the interest of time, let’s move on to the next question.”*

**Honduras Case Study:**

- What health issues are likely present for an adolescent girl such as Maria?
  - Maria may be experiencing significant emotional stress from being separated from her father; her physical ailments may be a manifestation of psychosomatic symptoms. We may be additionally concerned if she is depressed or has suicidal ideations or intent. Stay aware that true medical issues can coexist with psychosomatic complaints to ensure nothing gets missed.
- If Maria were to be relocated to your city or town, what barriers would you need to address to ensure she is able to receive appropriate care? What resources would you need to be able to do this?
  - Discuss with participants to see if they have staff who are trained in trauma-informed care; interpreters; social workers; and legal support to help reunite Maria with her father. See if there are political, policy, financial, social, or other barriers to receiving a child like Maria in their community. Consider resources outside the traditional healthcare system that may be culturally aligned such as faith-based groups.
- What capacities do you think Maria has to be able to overcome her current challenges?
  - Maria may have experienced gang violence and other social upheaval in her community, which is a common reason that children and adults attempt to flee Honduras. She has managed to survive thus far, and has thus demonstrated resilience. Enabling Maria to be around children her age who understand her language and culture may also help her find a sense of community.
  - For more examples of children’s experiences in Central America and upon arrival in the United States, please see: Kids in Need of Defense, Lutheran Immigration and Refugee Service, and Women’s Refugee Commission. [Betraying family values: How Immigration Policy at the United States Border is Separating Families](https://www.womensrefugeecommission.org/rights/resources/1450-betraying-family-values). Washington, DC, 2017.

**Somalia Case Study**:

- What health issues are likely present for an adolescent boy such as Abdul?
  - Abdul may be showing symptoms of malaria, which is common in Sub-Saharan Africa. Other possibilities could include dengue, anemia, tuberculosis, parasitic infections, or hepatitis. He also appears to have post-traumatic stress or another mental health illness from witnessing the killing of his family members. Consider whether any herbal or traditional remedies are being used currently. Consider herbal-drug interactions.
- If Abdul were to be resettled in your city or town, what barriers would you need to address to ensure he is able to receive adequate care? What resources would you need to be able to do this?
  - Discuss with participants to see if they have staff who are trained in trauma-informed care; interpreters, and mental health providers who understand the context of conflict and displacement in Somalia and Uganda. Abdul’s mother may also not have had much exposure to Western biomedicine, and may benefit from longer visits so that she is introduced to different medical practices in the United States. See if there are political, policy, financial, social, or other barriers to receiving an adolescent such as Abdul in their community. Consider the experience of Somali refugees who often are both black and Muslim, thus may experience multiple forms of discrimination. This is an opportunity to discuss “intersectionality” or when multiple barriers become additive.
- What capacities do you think Abdul and his family have to overcome their current challenges?
  - Abdul and his family have experienced tremendous hardship as they fled conflict and endured discrimination. The mother has shown much perseverance to bring her family to the United States in search of a better life. Abdul has a family that cares for him. If the community may have resettled Somali refugees in the past, they may serve as a resource for Abdul and his family. Consider who may be a trusted authority figure for a teen male. This may be someone outside of the family, such as a role model in the community.
- If Abdul were a Somali adolescent girl, what additional concerns would we like to consider?
  - An adolescent girl is at increased risk of sexual and gender-based violence during displacement. In some contexts, she may also not have had much autonomy to make her own decisions, and thus, it may take some time to develop trust and the space for her to feel comfortable sharing her feelings and opinions. Recognize the risk of sexual and gender-based violence exists for boys as well. Consider role of “Orientalism”in perceptions of Muslim girls and women.
  - For more information on challenges for adolescents in displacement contexts, please see: Plan International. [A time of Transition: Adolescents in Emergencies](https://plan-international.org/publications/time-transition-adolescents-humanitarian-settings#download-options). 2016.
  - For more information on Orientalism, please see: <https://rlp.hds.harvard.edu/faq/orientalism> (Accessed April, 5, 2020)

**Haiti Case Study:**

- What are your top 3 differentials for Jean-Louis’s complaint and why? How would you confirm your diagnosis and manage it if correct?
  - The most likely diagnosis is Giardia, given the intermittent diarrhea, greasy stools, poor weight gain, and the stool ova and parasite results. Other differentials include strongyloidiasis, schistosomiasis, or other infections. Management of Giardia includes metronidazole, tinidazole, or nitazoxanide. Obtain a detailed diet history. Consider asking the mother to keep a diet and symptom journal over a specific period of time (3 days, 2 weeks, etc). Clarify if mother is breastfeeding or bottle feeding or both. If the mother is bottle feeding with formula, ask the mother if she is mixing formula as directed or is overdiluting it.
- What are some of the biological risk factors Jean-Louis faced prior to his arrival in the US? What are some of the psychosocial risk factors?
  - Some biological risk factors that Jean-Louis faces include preterm birth, lack of access to clean water and good sanitation, and limited nutrition while in Haiti. Psychosocial risk factors include the family’s experiences post-earthquake and limited resources to care for Jean-Louis. Consider the role of maternal health and maternal mental health.
- What are some of his ongoing biopsychosocial risk factors?
  - Ongoing risk factors include past trauma, cultural adjustments, and the family’s financial resources. Consider potential postpartum depression in case. Consider the infant and mother as part of an interdependent dyad.
- What about current protective factors? What resources might the family be able to access?
  - Jean Louis’s immediate and extended family may serve as a protective resource as he grows in a new country. The family may be able to access resources from the resettlement center or other community centers/community-based organizations.
- Are there any referrals you would consider making for the patient or family?
  - Referrals to Social Work may be helpful for the family as they adjust to life, navigate health care, and are linked to services in the United States.
  - For more information around experiences of children post-earthquake, please see Satchit Balsari, Jay Lemery, Timothy P. Williams, Brett D. Nelson. Protecting the Children of Haiti. *New England Journal of Medicine.* 2010 Mar 4;362(9):e25. <https://www.nejm.org/doi/pdf/10.1056/NEJMp1001820>

**General Whole Group discussion for Report Back After Break Out Sessions:**

- What were elements of the case on which you wanted more information?
- What are some of the differences of opinion that existed within your group? Did you resolve them? If so, how did you resolve them?
- What did you learn from others in your group?
- What will you take back to apply to your own practice or setting going forward?

**Complementary Curricula on MedEdPORTAL:**

Farokhi M, Vivanco R, Muck A. An interprofessional collaborative student-run clinic by dental, nursing and medical faculty and students at University of Texas Health Science Center at San Antonio for refugees. MedEdPORTAL. 2013;9:9497.

Stone H, Choi R, Aagaard E, et al. Refugee health elective. MedEdPORTAL. 2013;9:9457.

Nicklas D, Wong C, Moloo J, Kaul P. The refugee health II elective. MedEdPORTAL. 2014;10:9940.

Fitzgerald SN, Leslie KF, Simpson R, Jones VF, Barnes ET. Culturally effective care for refugee populations: interprofessional, interactive case studies. MedEdPORTAL. 2018;14:10668.

**Additional Reading and References:**

Anderson, L. W., Krathwohl, D. R., & Bloom, B. S. (2001). A taxonomy for learning, teaching, and assessing: A revision of Bloom's Taxonomy of educational objectives (Complete ed.). New York: Longman.

Policy Statement: Patient- and family-centered care and the pediatrician’s role. Pediatrics. 2012;129(2):394–404. Available at: <http://pediatrics.aappublications.org/content/129/2/394>. Reaffirmed February 2018

Elwyn G, Frosch D, Thomson R, et al. Shared decision making: a model for clinical practice. J Gen Intern Med. 2012;27(10):1361–1367pmid:22618581

Suphanchaimat R, Kantamaturapoj K, Putthasri W, Prakongsai P. Challenges in the provision of healthcare services for migrants: a systematic review through providers' lens. BMC Health Serv Res. 2015;15:390.

Robertshaw L, Dhesi S, Jones LL Challenges and facilitators for health professionals providing primary healthcare for refugees and asylum seekers in high-income countries: a systematic review and thematic synthesis of qualitative research. BMJ Open. 2017;7:e015981. doi: 10.1136/bmjopen-2017-015981

Im, H., Swan, L.E.T. Capacity building for refugee mental health in resettlement: implementation and evaluation of cross-cultural trauma-informed care training. J Immigrant Minority Health. 2020.

Howard, J. A., & Renfrow, D. G. (2014). Intersectionality. In J. D. McLeod, E. J. Lawler, & M. & Schwalbe (Eds.), Handbook of the social psychology of inequality (pp. 95–121). Springer.

Harvard Divinity School Religious Literacy Project. Orientalism. Available at: <https://rlp.hds.harvard.edu/faq/orientalism> Accessed April 5, 2020

Reliefweb. Faith groups speak out together on World Refugee Day. Available at: <https://reliefweb.int/report/world/faith-based-groups-speak-out-together-world-refugee-day> Accessed April 6, 2020
