# Supplementary material for: Pediatric Refugee Health Care Delivery in the Community Setting: An Educational Workshop for Multidisciplinary Family-Centered Care During Resettlement
Source: MedEdPORTAL. 2020 Nov 3;16:10988. doi: 10.15766/mep_2374-8265.10988 (PMC7666829; doi:10.15766/mep_2374-8265.10988)
Supplement: Supplementary file 1 — Agenda.docxPresentation 1 Intro to Refugees.pptxPresentation 2 Health Screening.pptxCases.docxPresentation 3 Trauma-Informed Care.pptxPresentation 4 Refugee Health Advocacy.pptxRefugee Workshop Evaluation.docx [file mep_2374-8265.10988-s001.zip › E. Presentation 3 Trauma-Informed Care.pptx]

## Slide 1
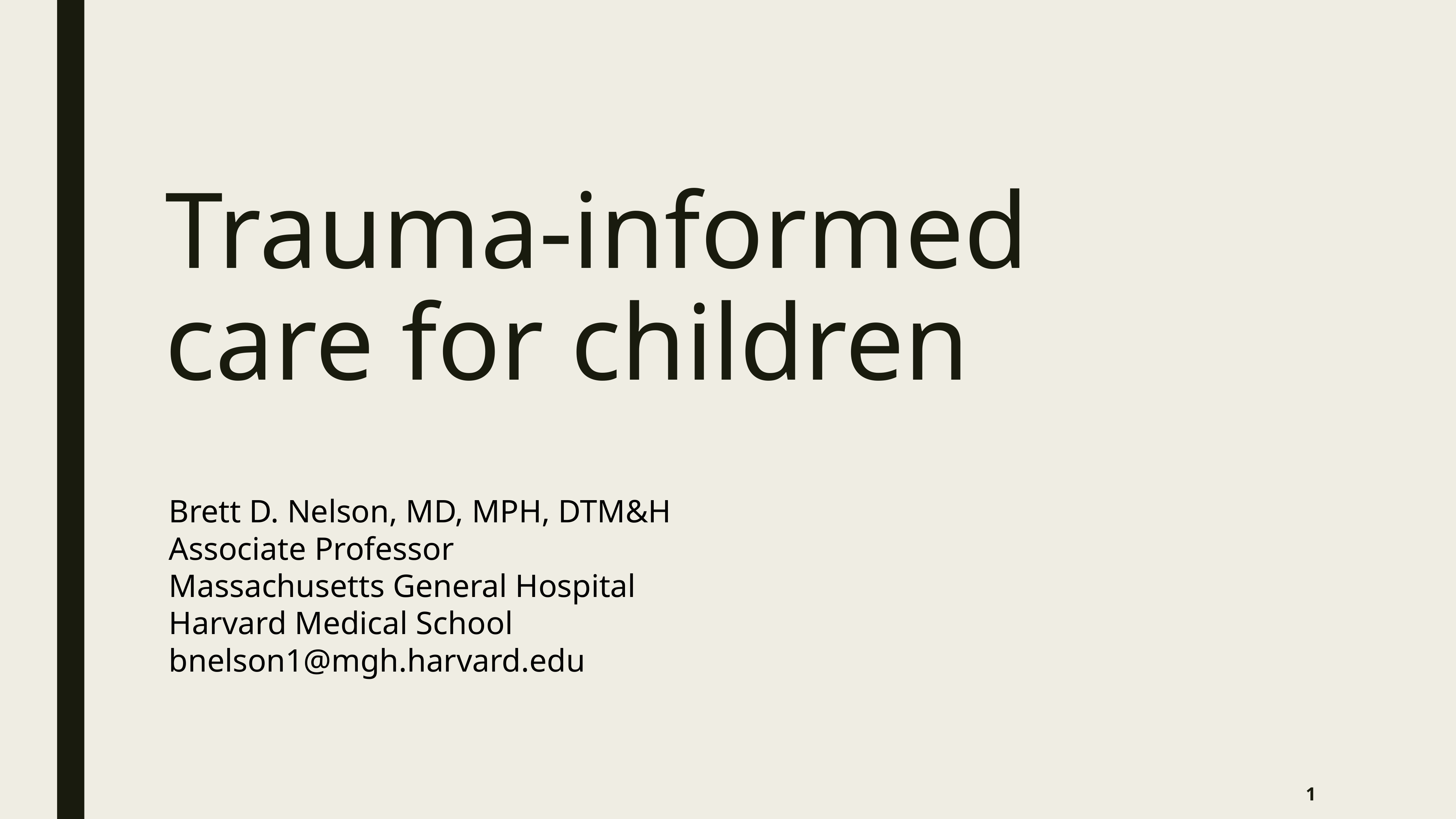

# Trauma-informed care for children
Brett D. Nelson, MD, MPH, DTM&H
Associate Professor
Massachusetts General Hospital
Harvard Medical School
bnelson1@mgh.harvard.edu
1

## Slide 2
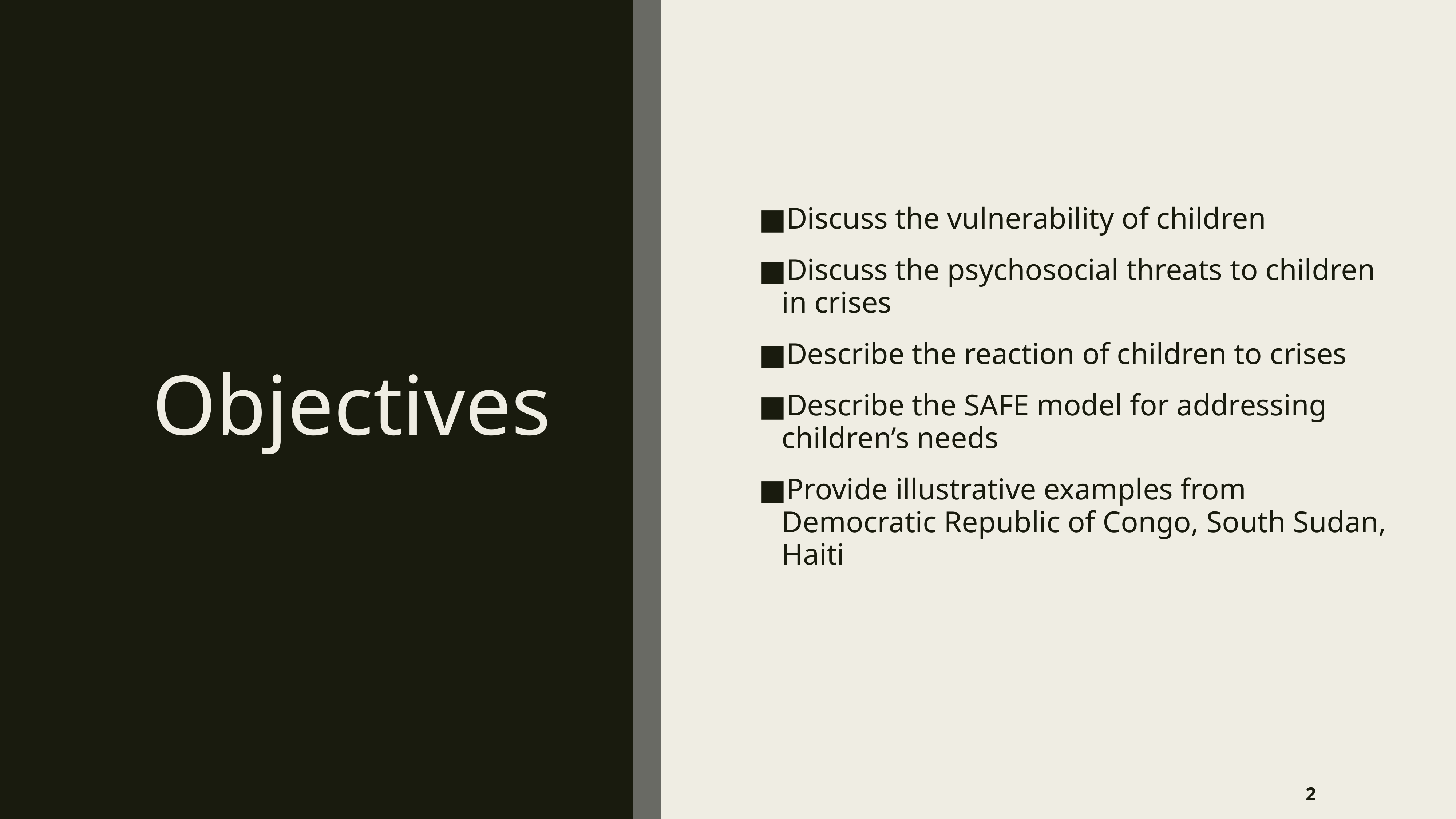

Discuss the vulnerability of children
Discuss the psychosocial threats to children in crises
Describe the reaction of children to crises
Describe the SAFE model for addressing children’s needs
Provide illustrative examples from Democratic Republic of Congo, South Sudan, Haiti
# Objectives
2

## Slide 3
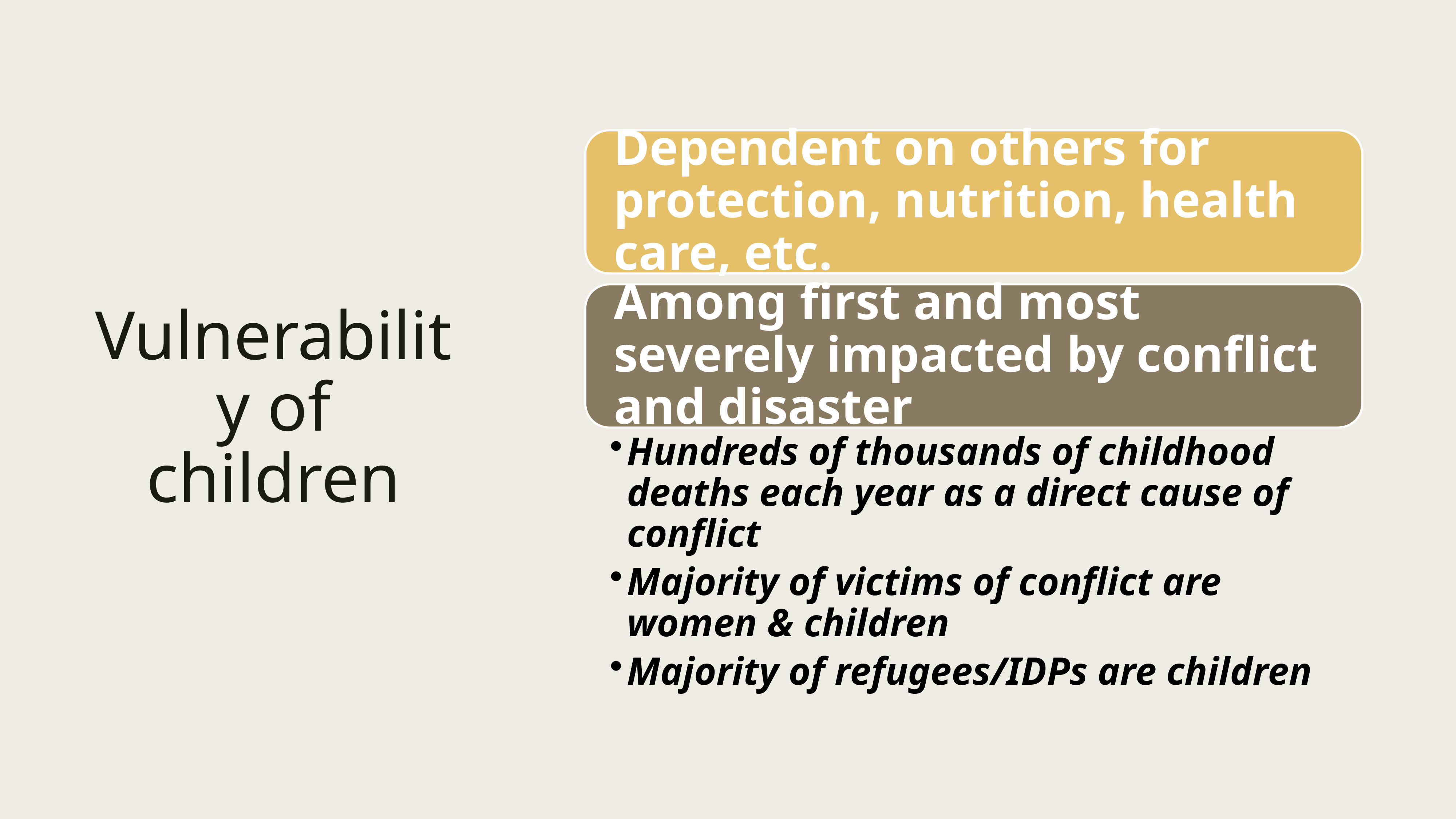

# Vulnerability of children
3

## Slide 4
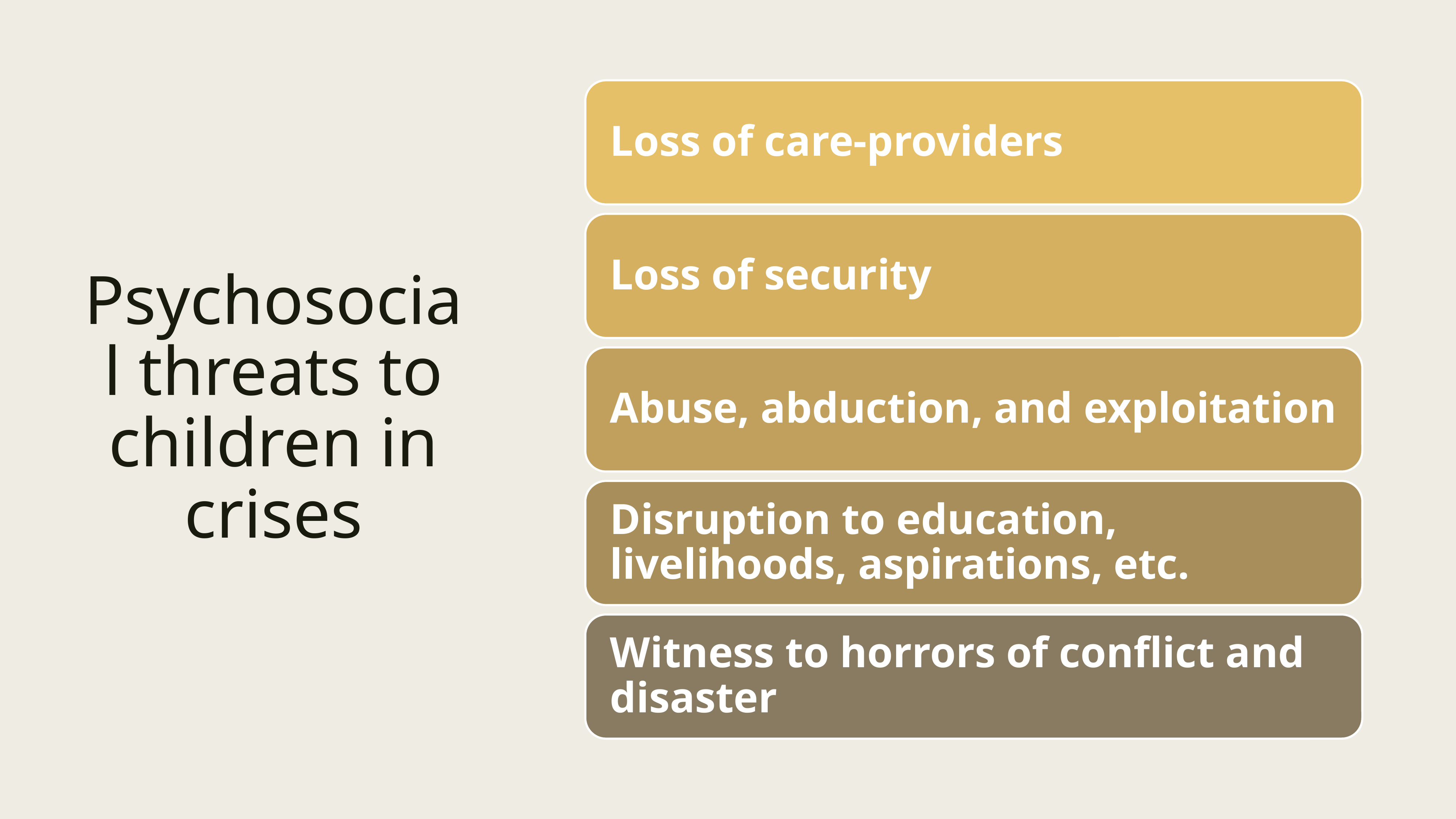

# Psychosocial threats to children in crises
4

## Slide 5
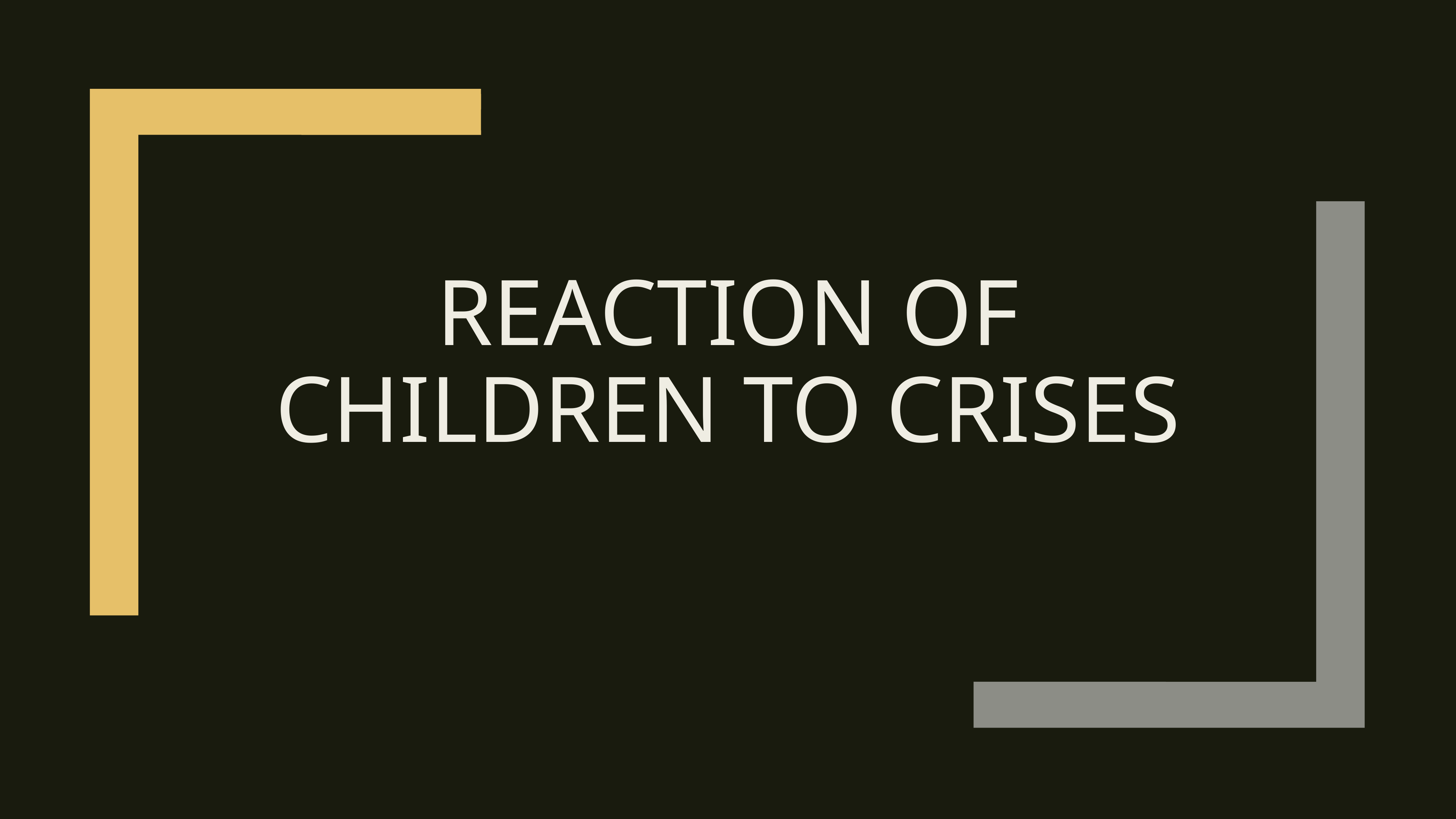

# Reaction of children to crises

## Slide 6
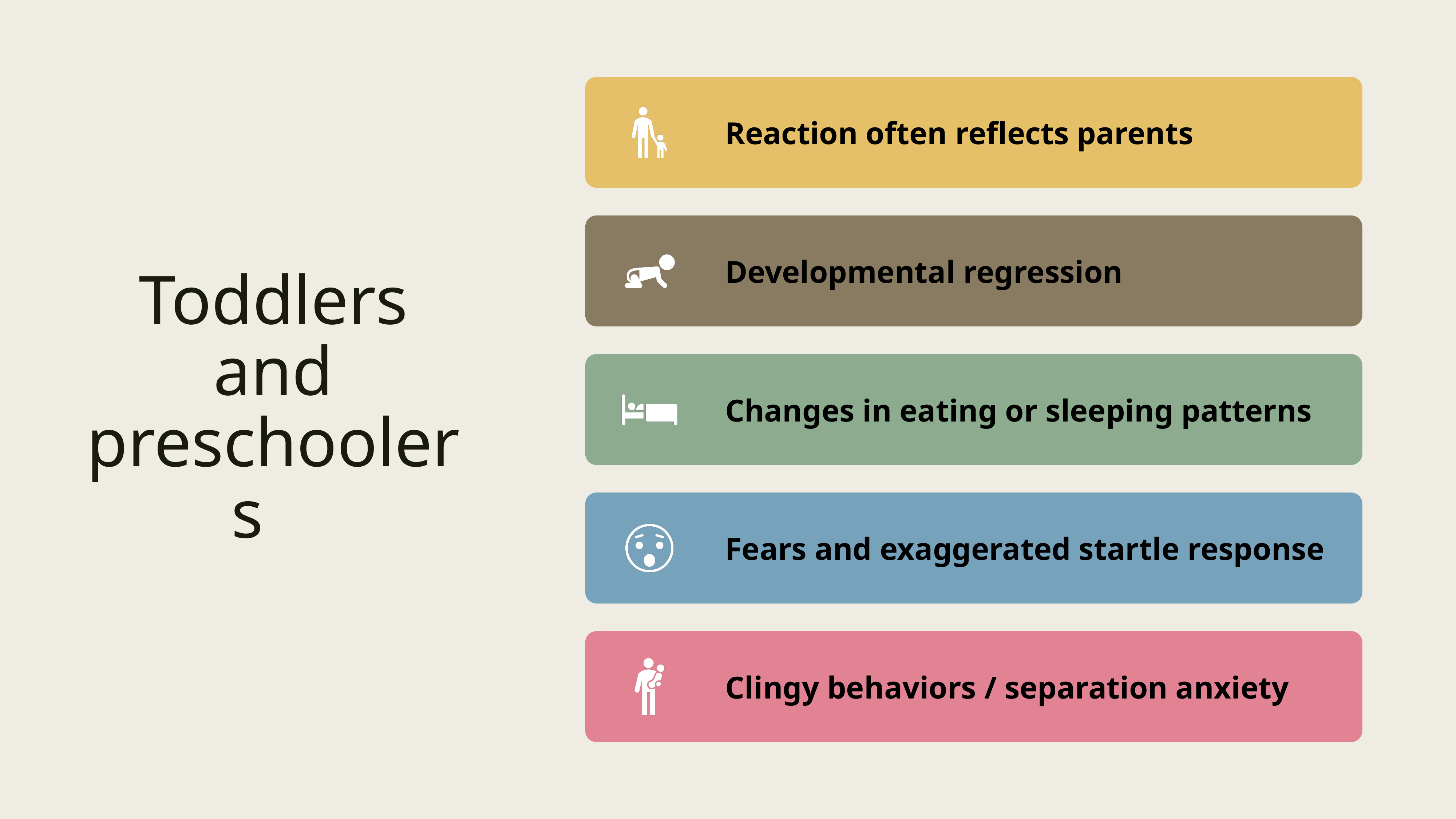

# Toddlers and preschoolers
6

## Slide 7
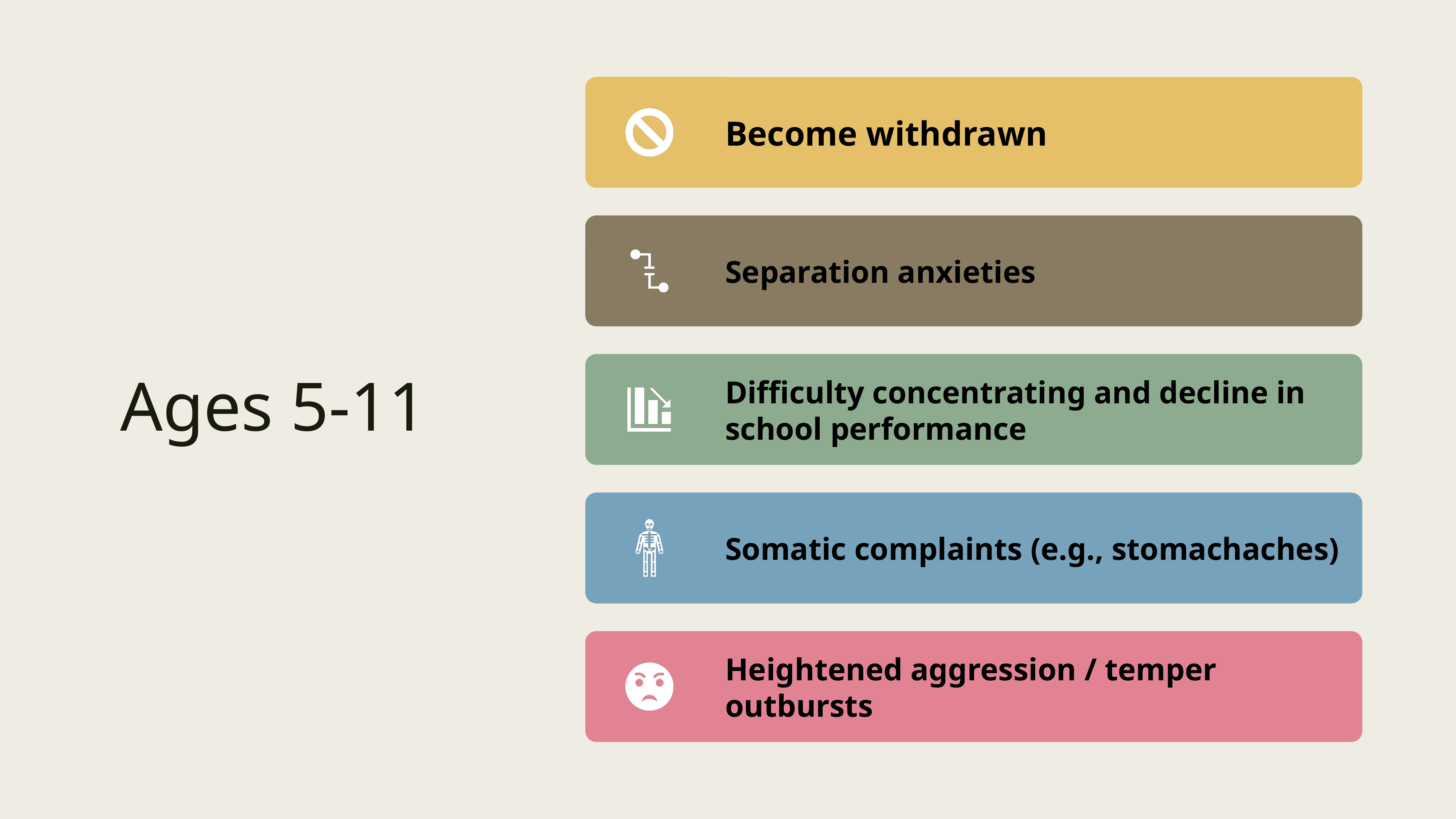

# Ages 5-11
7

## Slide 8
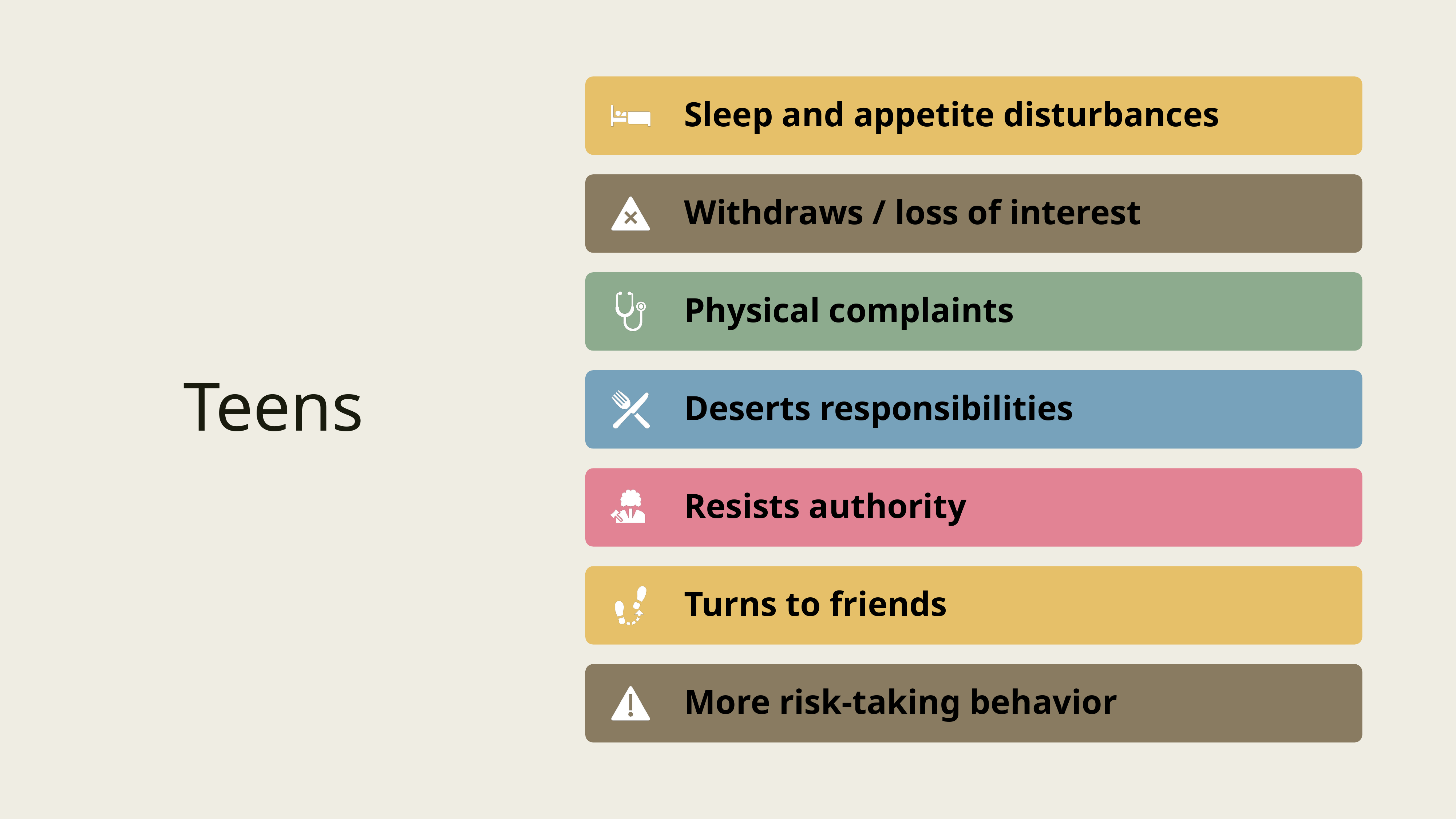

# Teens
8

## Slide 9
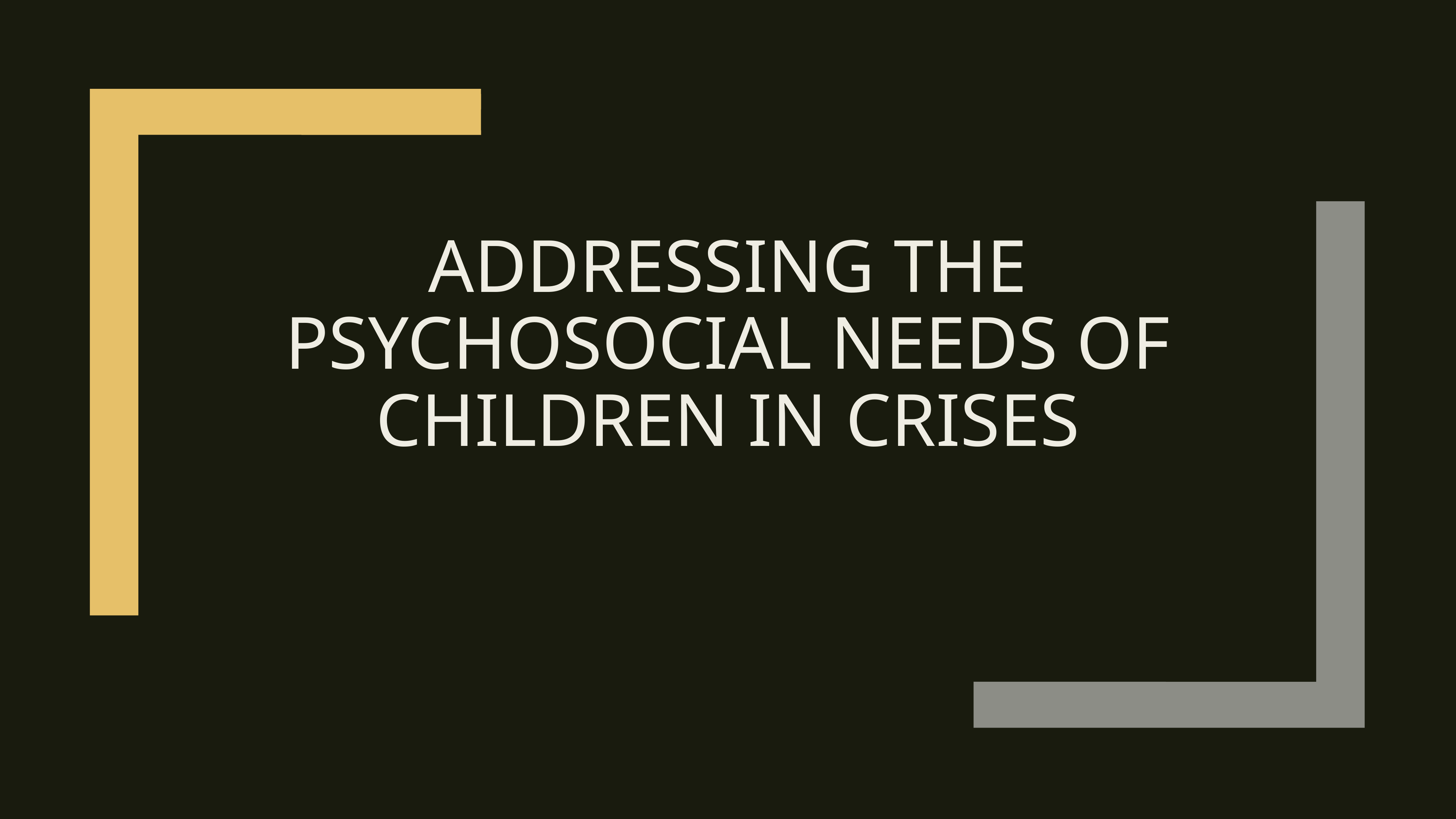

# Addressing the psychosocial needs of children in crises

## Slide 10
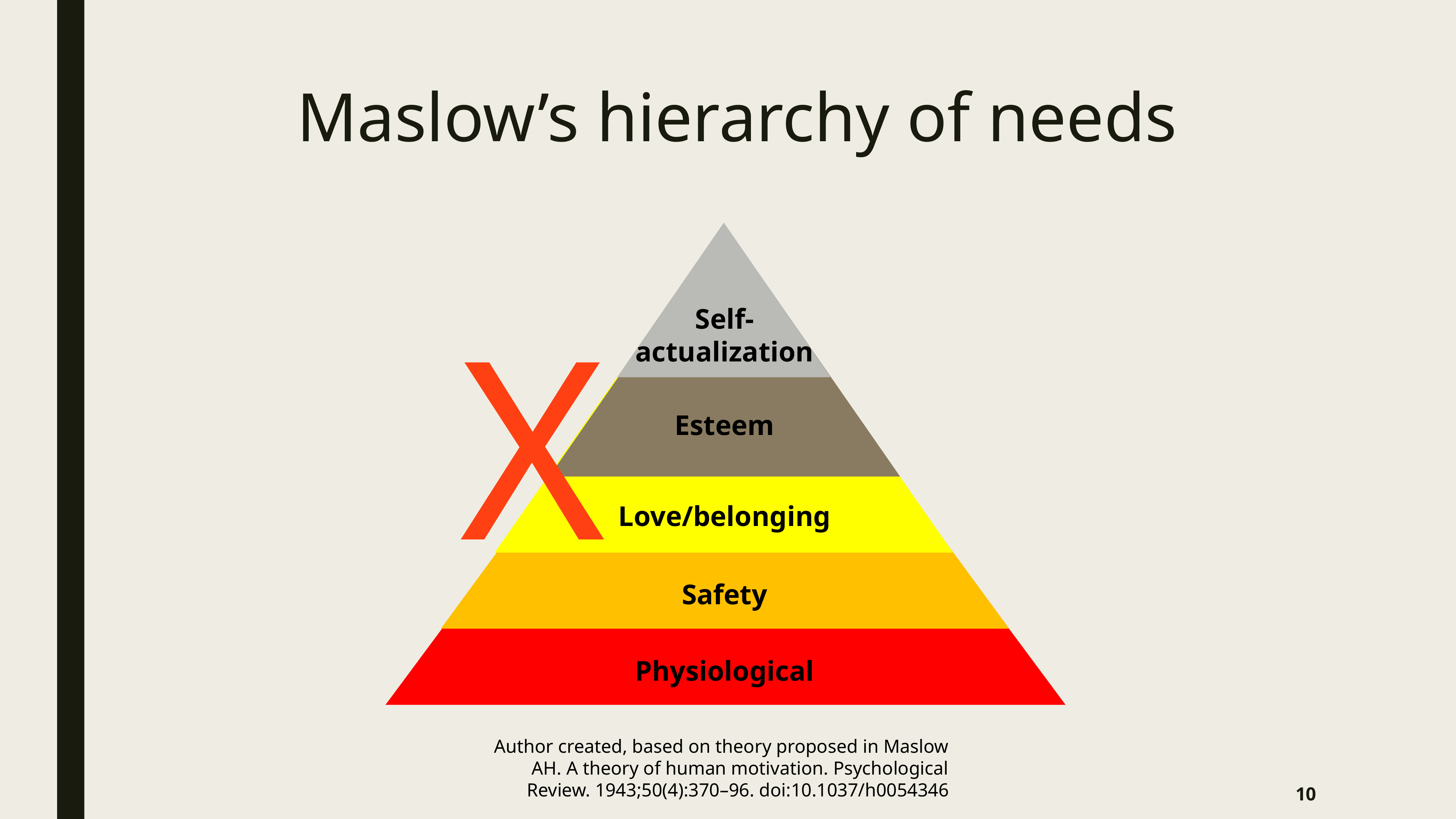

# Maslow’s hierarchy of needs
X
Self-
actualization
Esteem
Love/belonging
Safety
Physiological
Author created, based on theory proposed in Maslow AH. A theory of human motivation. Psychological Review. 1943;50(4):370–96. doi:10.1037/h0054346
10

## Slide 11
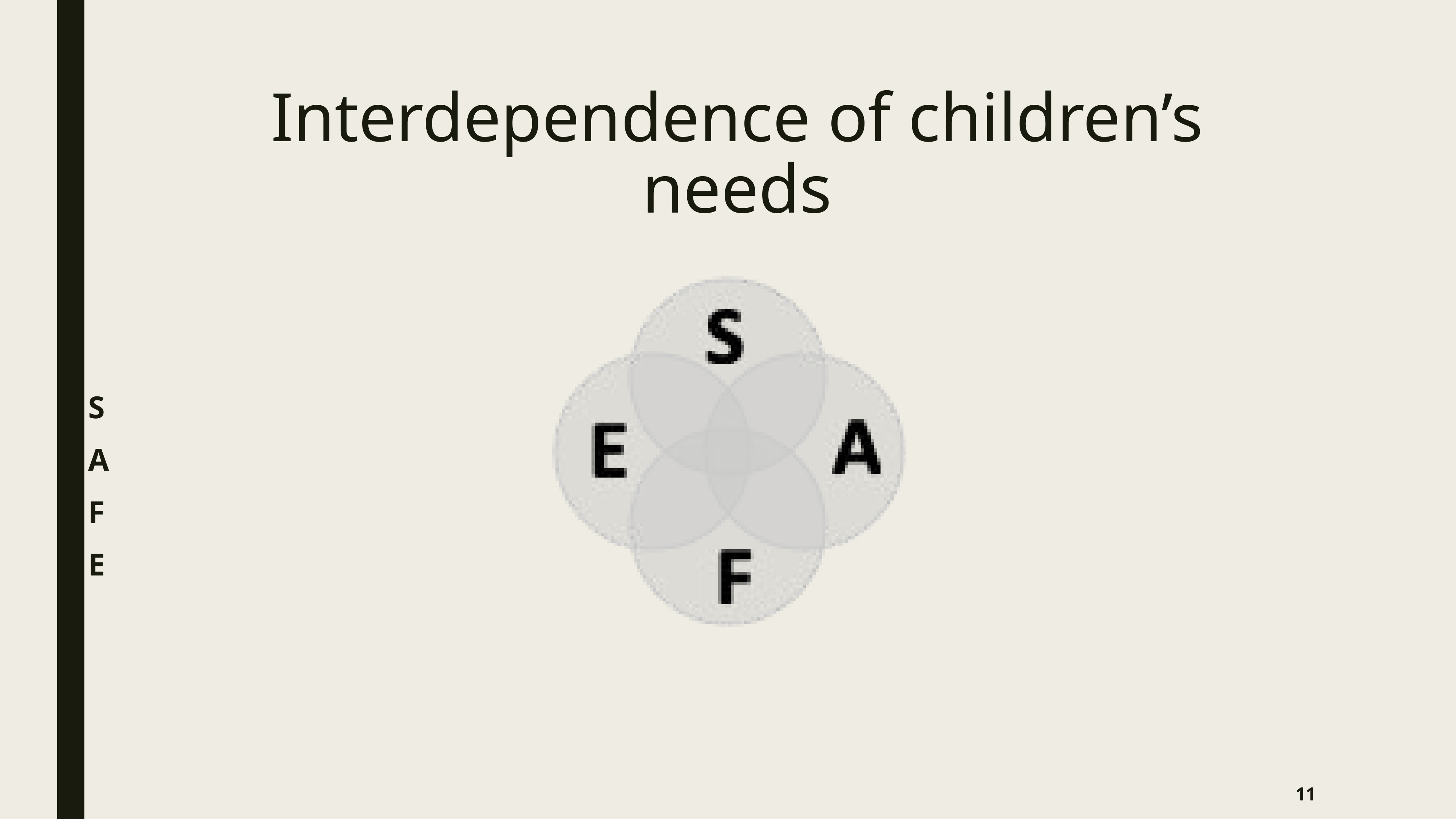

# Interdependence of children’s needs
S
A
F
E
11

## Slide 12
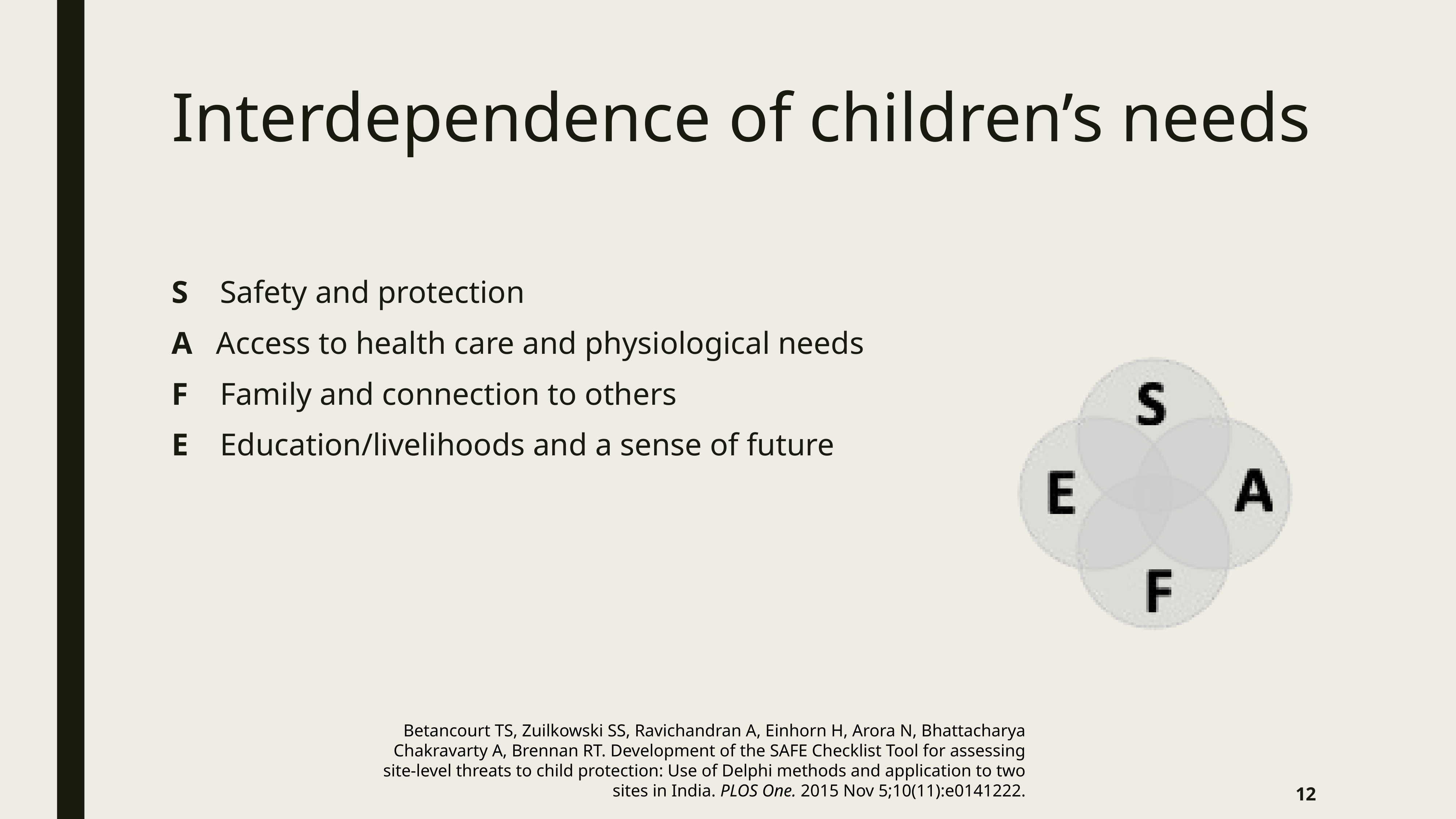

# Interdependence of children’s needs
S Safety and protection
A Access to health care and physiological needs
F Family and connection to others
E Education/livelihoods and a sense of future
Betancourt TS, Zuilkowski SS, Ravichandran A, Einhorn H, Arora N, Bhattacharya Chakravarty A, Brennan RT. Development of the SAFE Checklist Tool for assessing site-level threats to child protection: Use of Delphi methods and application to two sites in India. PLOS One. 2015 Nov 5;10(11):e0141222.
12

## Slide 13
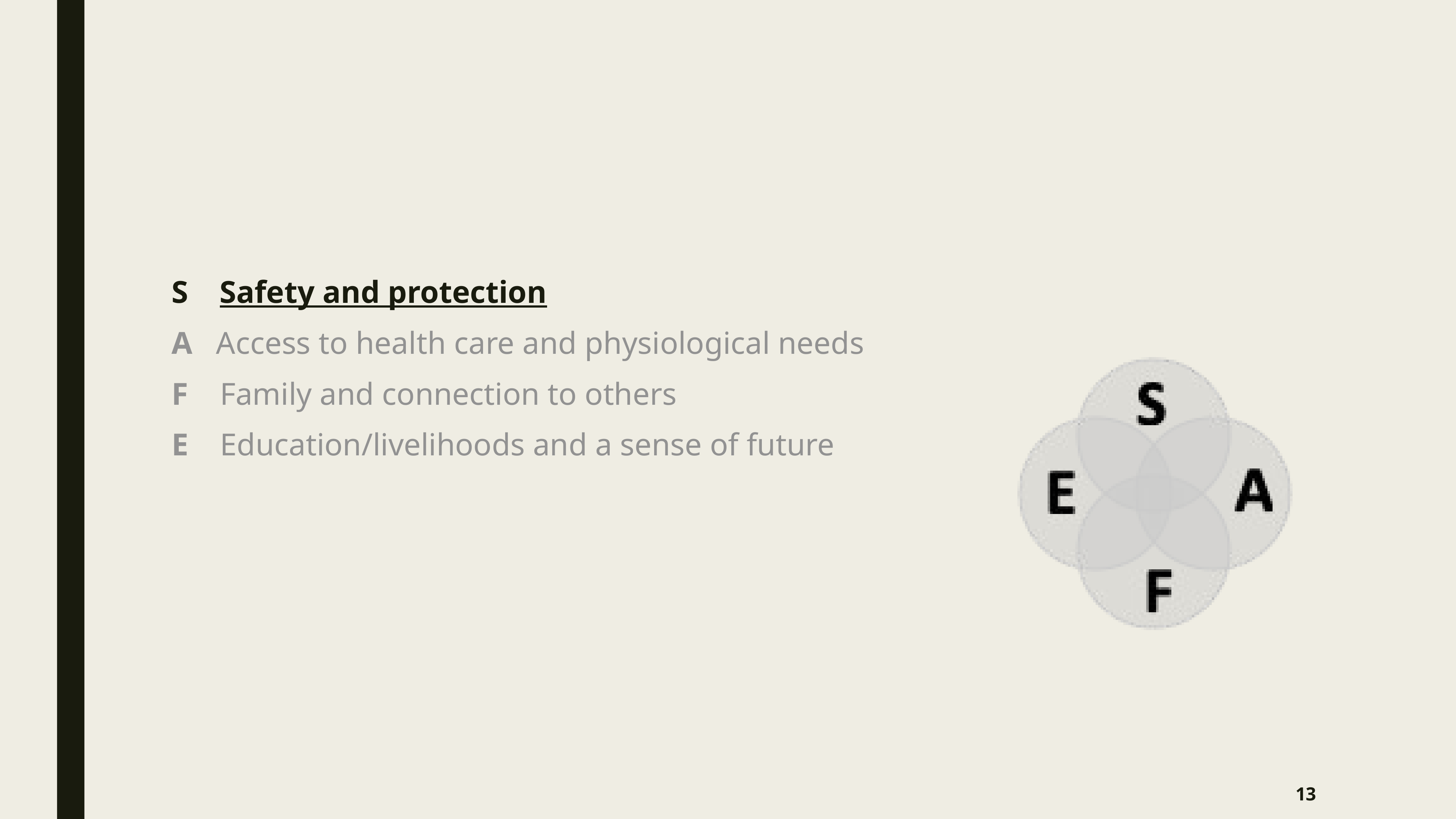

S Safety and protection
A Access to health care and physiological needs
F Family and connection to others
E Education/livelihoods and a sense of future
13

## Slide 14
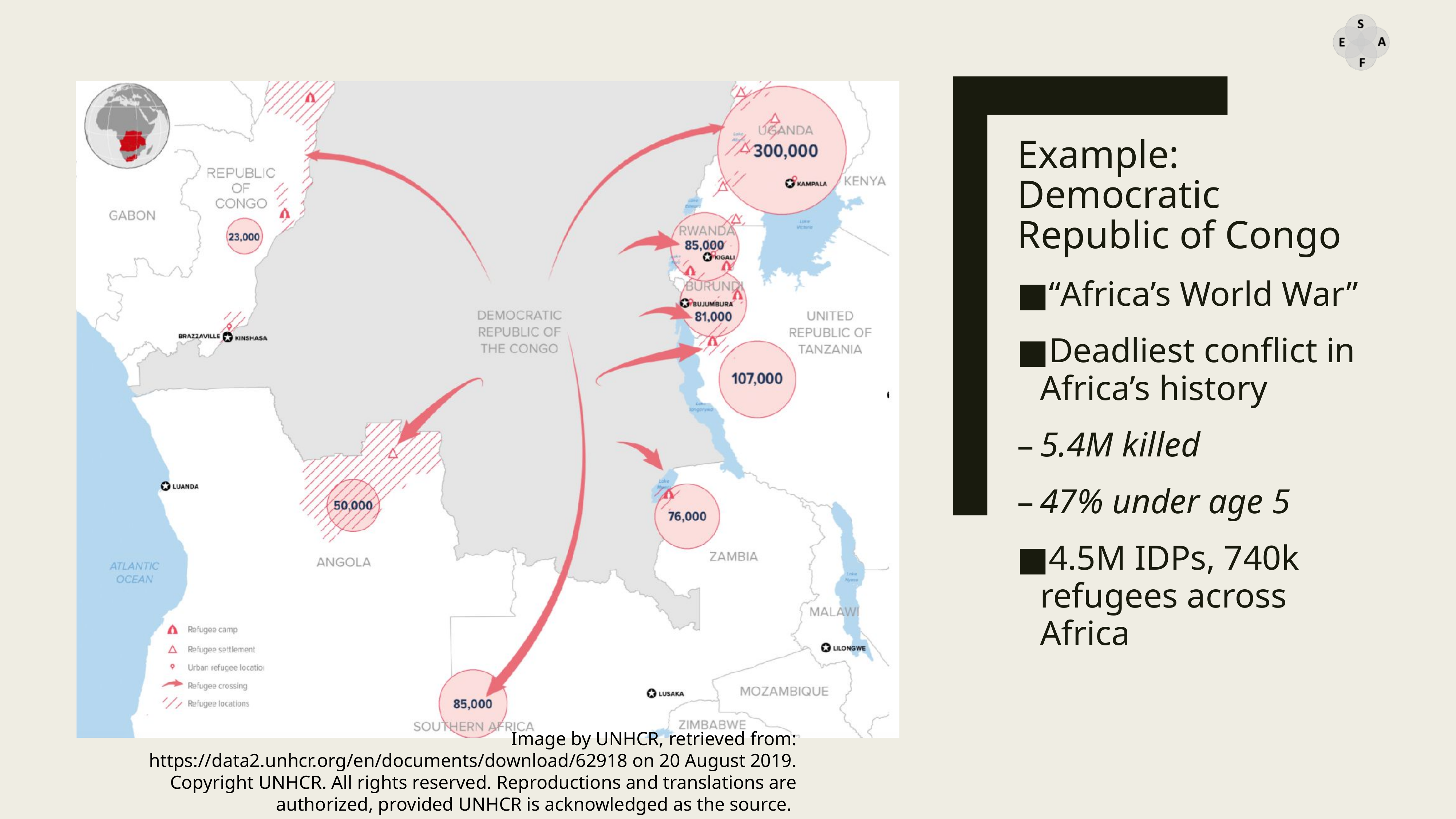

# Example: Democratic Republic of Congo
“Africa’s World War”
Deadliest conflict in Africa’s history
5.4M killed
47% under age 5
4.5M IDPs, 740k refugees across Africa
Image by UNHCR, retrieved from: https://data2.unhcr.org/en/documents/download/62918 on 20 August 2019. Copyright UNHCR. All rights reserved. Reproductions and translations are authorized, provided UNHCR is acknowledged as the source.
14

## Slide 15
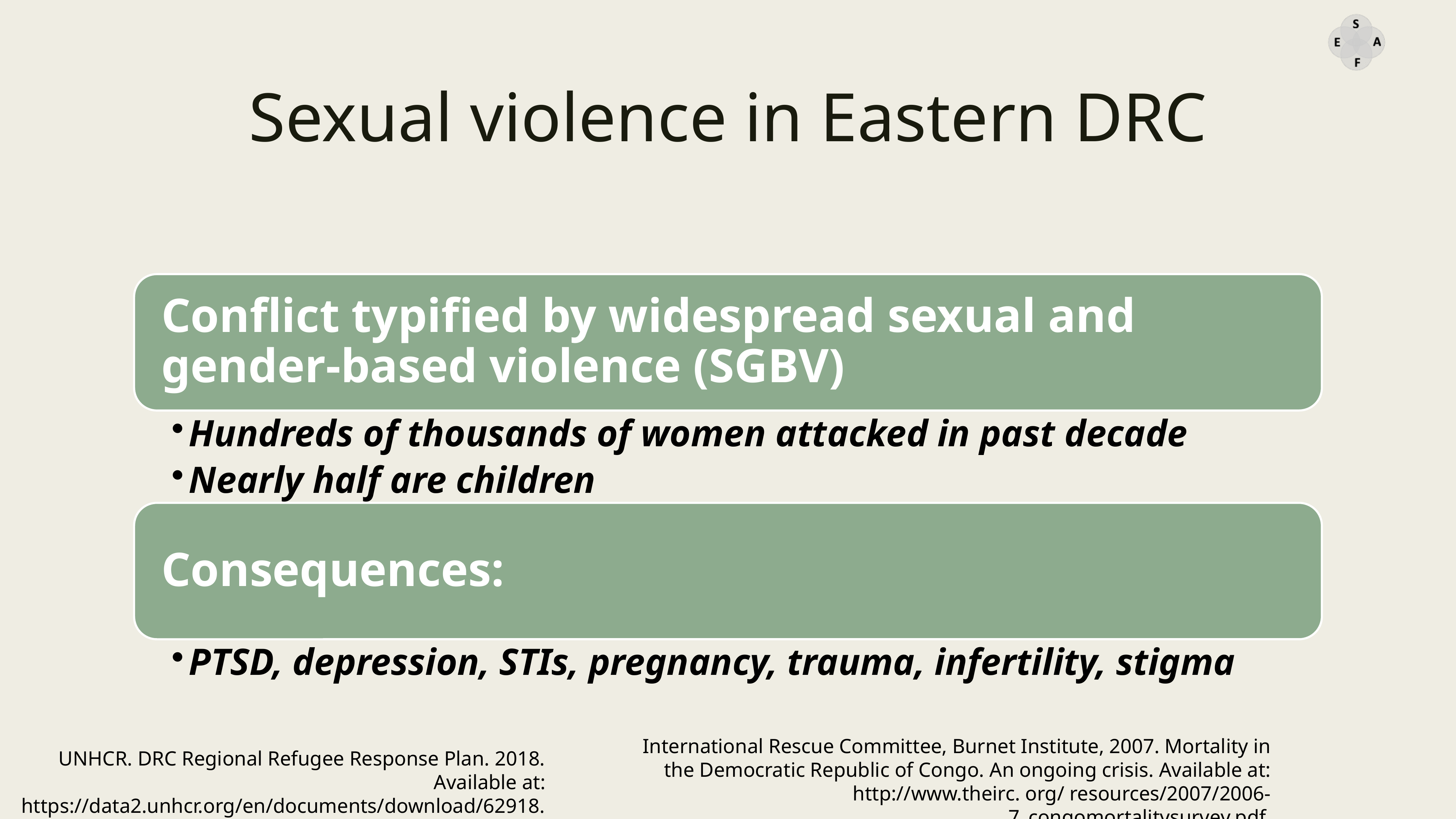

# Sexual violence in Eastern DRC
International Rescue Committee, Burnet Institute, 2007. Mortality in the Democratic Republic of Congo. An ongoing crisis. Available at: http://www.theirc. org/ resources/2007/2006-7_congomortalitysurvey.pdf
UNHCR. DRC Regional Refugee Response Plan. 2018. Available at: https://data2.unhcr.org/en/documents/download/62918.
15

## Slide 16
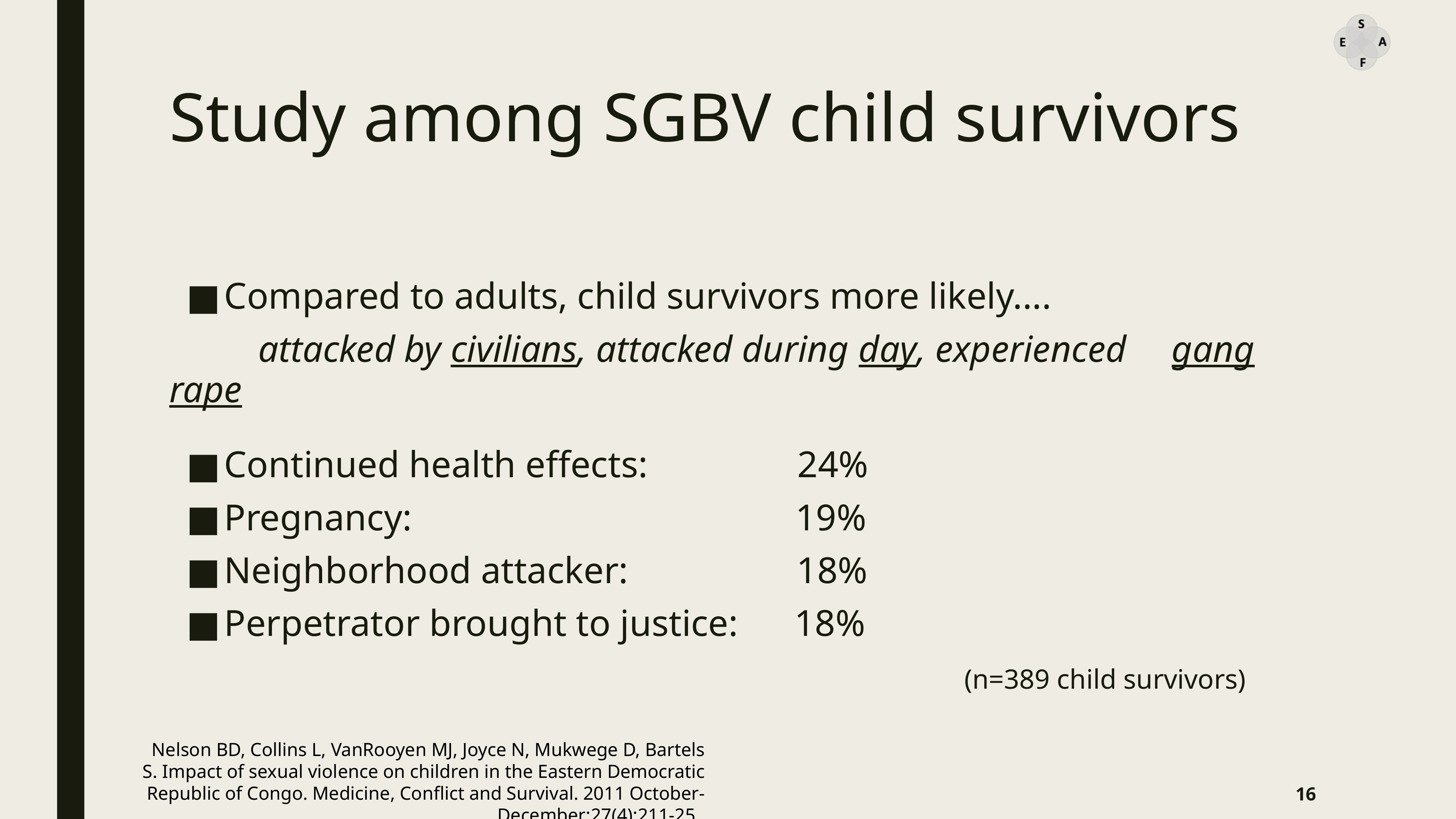

# Study among SGBV child survivors
Compared to adults, child survivors more likely....
attacked by civilians, attacked during day, experienced 					gang rape
Continued health effects: 24%
Pregnancy: 19%
Neighborhood attacker: 18%
Perpetrator brought to justice: 18%
 (n=389 child survivors)
Nelson BD, Collins L, VanRooyen MJ, Joyce N, Mukwege D, Bartels S. Impact of sexual violence on children in the Eastern Democratic Republic of Congo. Medicine, Conflict and Survival. 2011 October-December;27(4):211-25.
16

## Slide 17
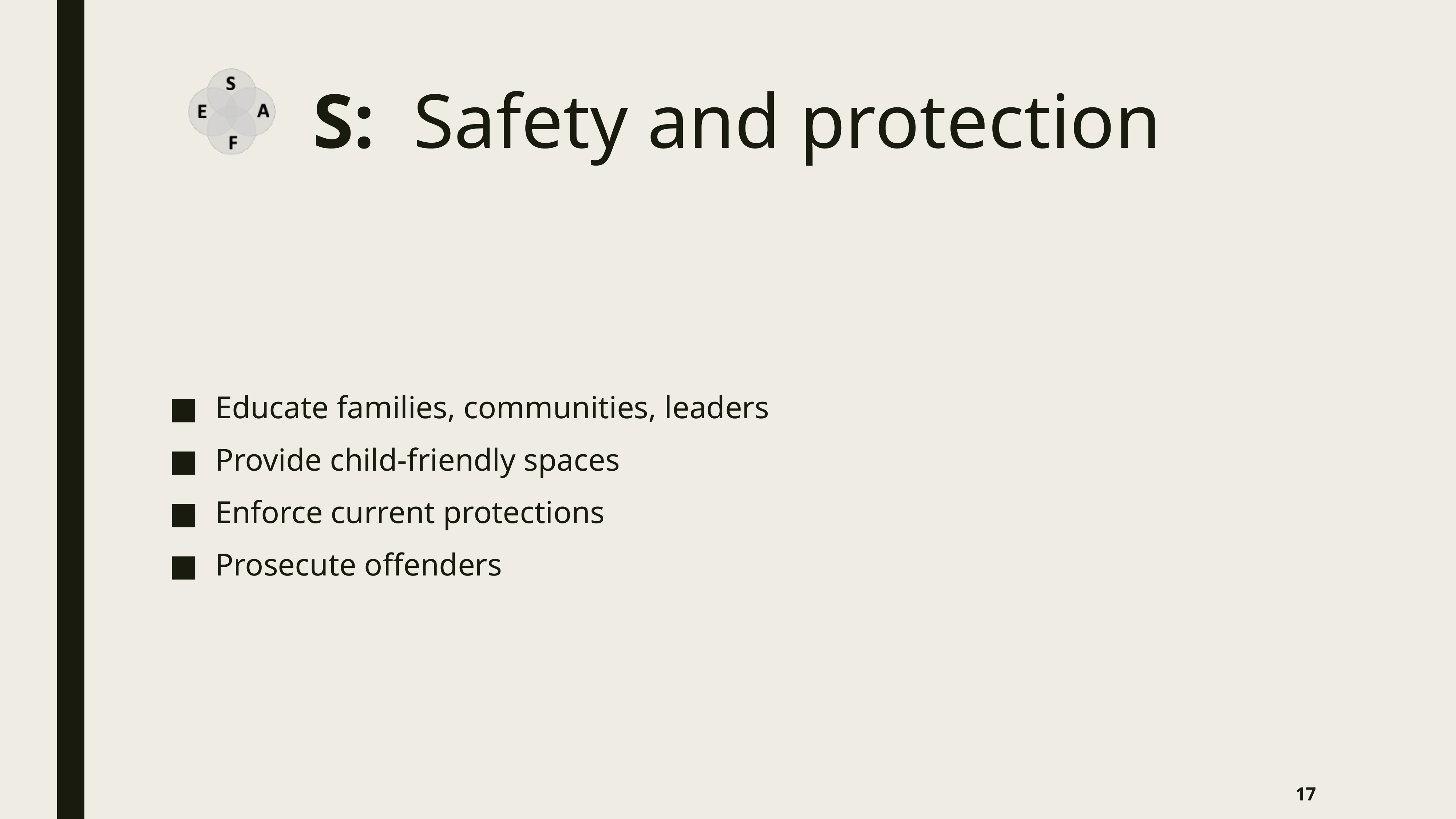

# S: Safety and protection
Educate families, communities, leaders
Provide child-friendly spaces
Enforce current protections
Prosecute offenders
17

## Slide 18
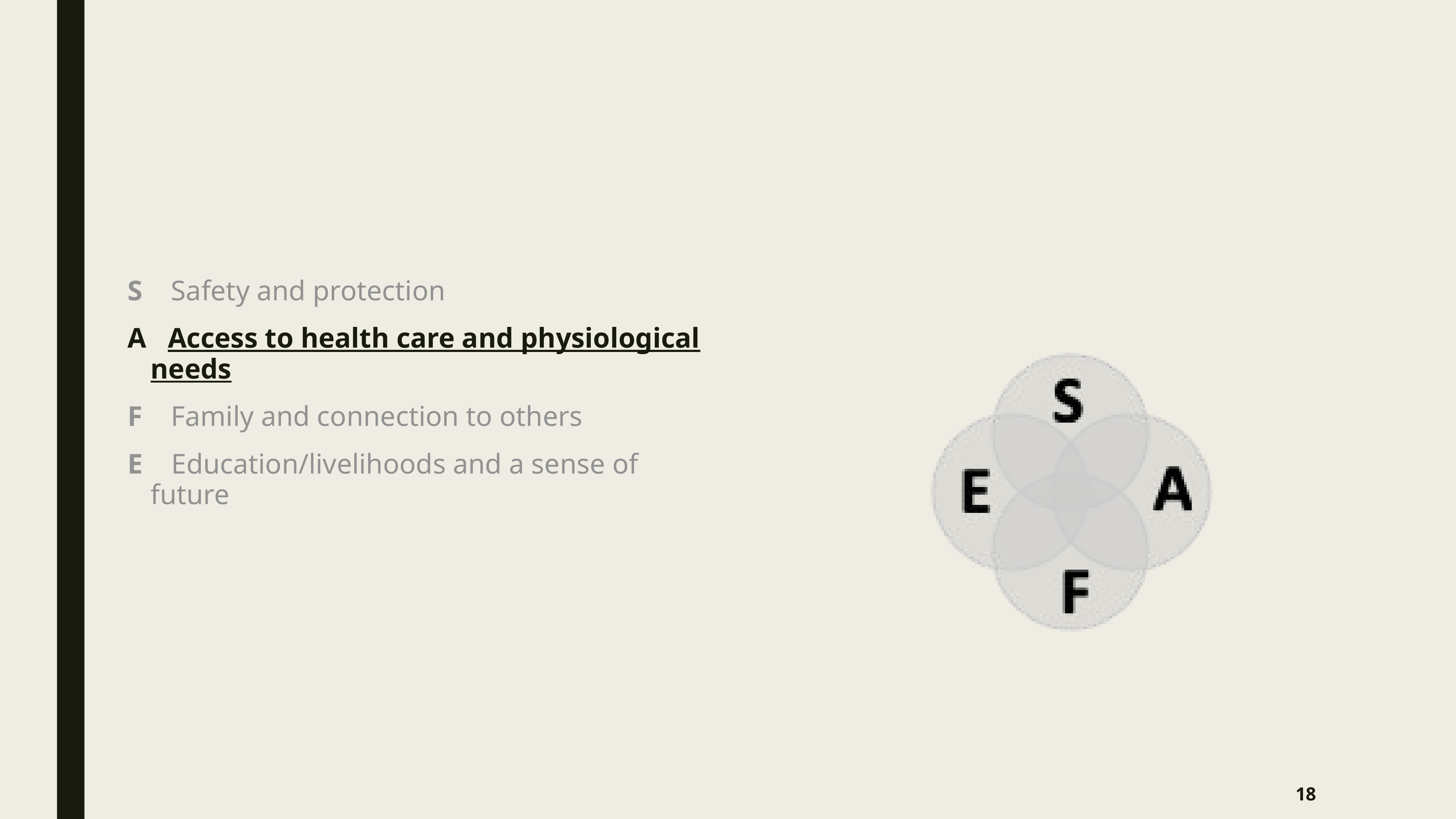

S Safety and protection
A Access to health care and physiological needs
F Family and connection to others
E Education/livelihoods and a sense of future
18

## Slide 19
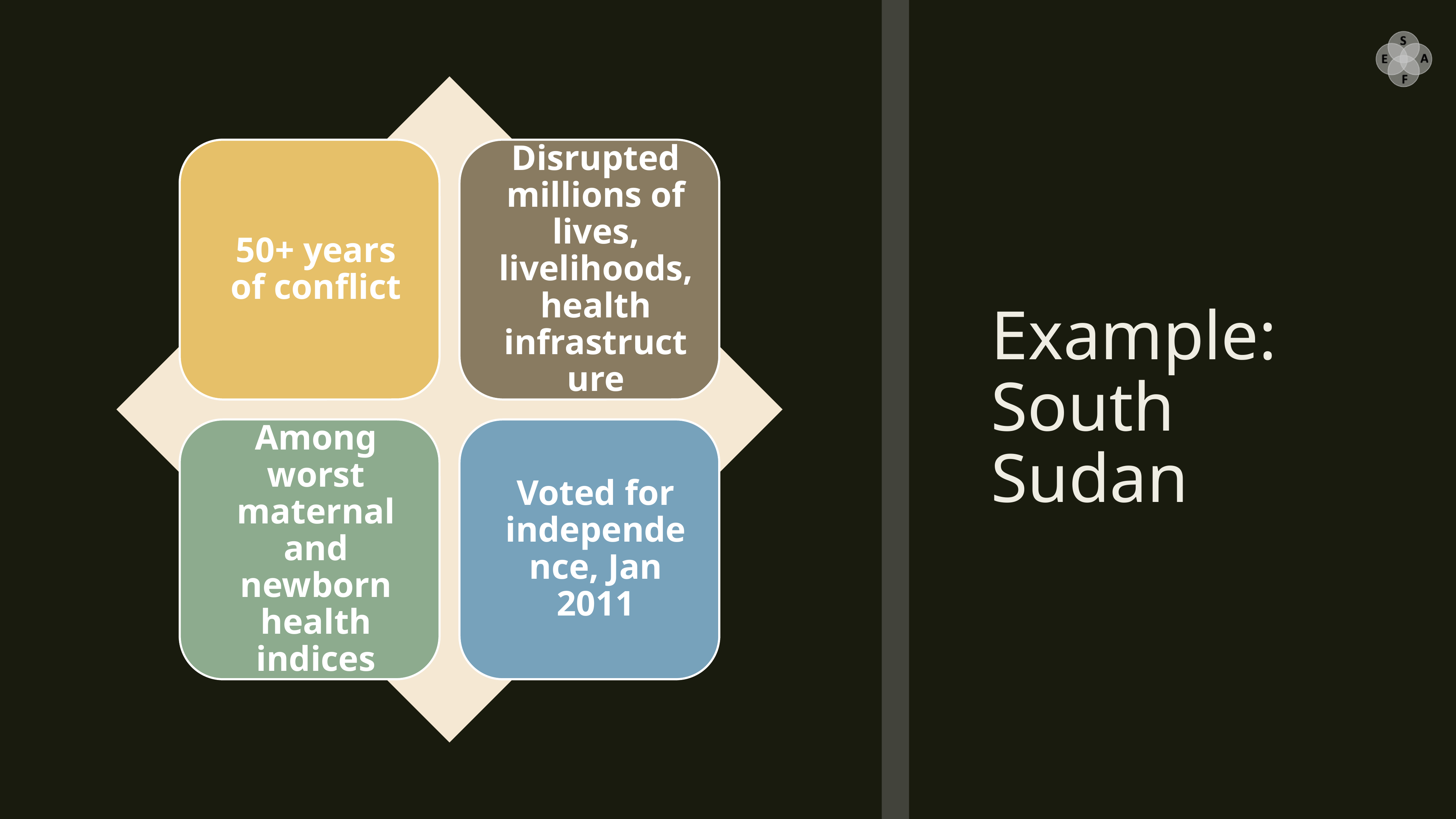

# Example: South Sudan
19

## Slide 20
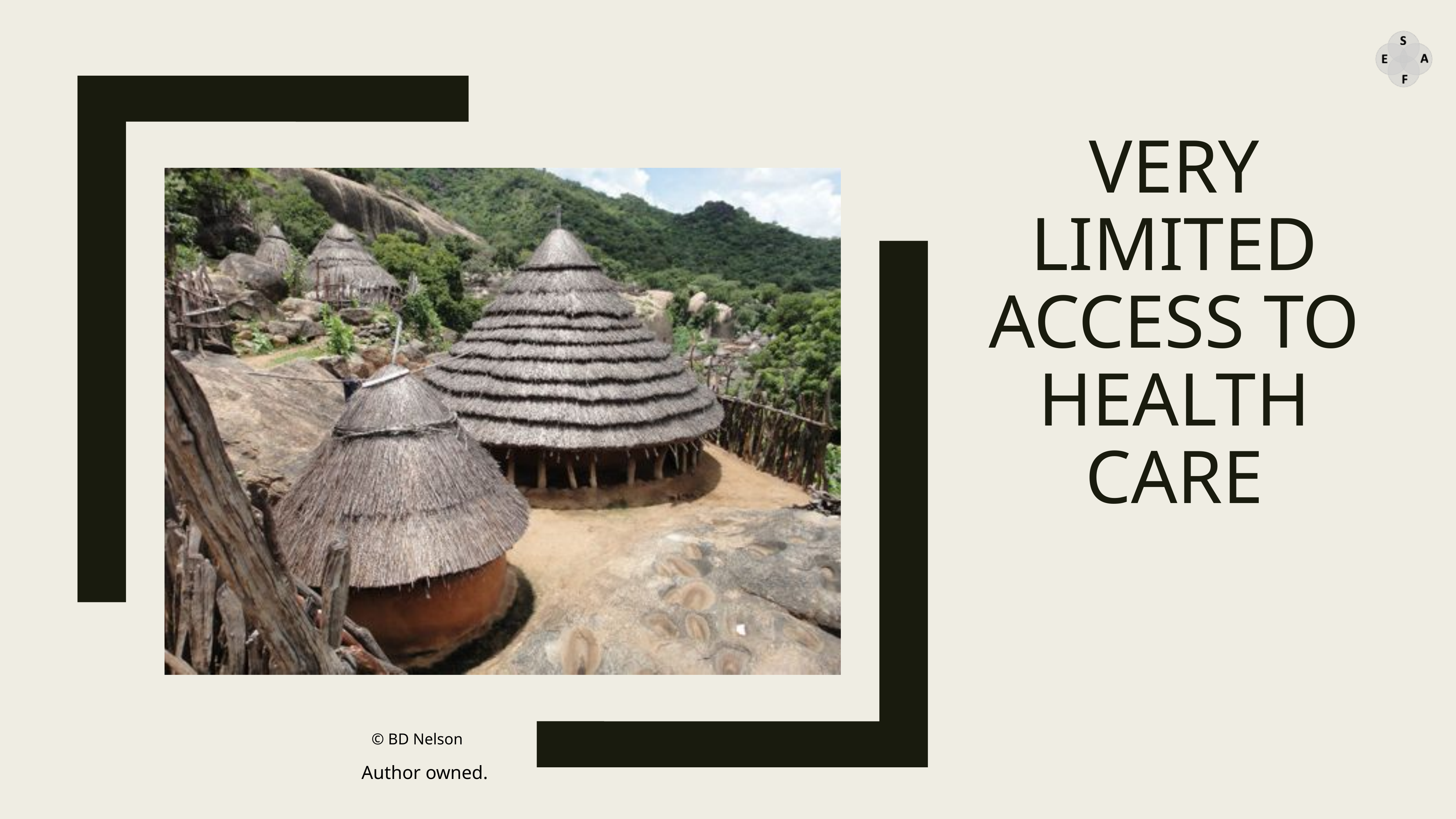

# Very limited access to health care
© BD Nelson
Author owned.
20

## Slide 21
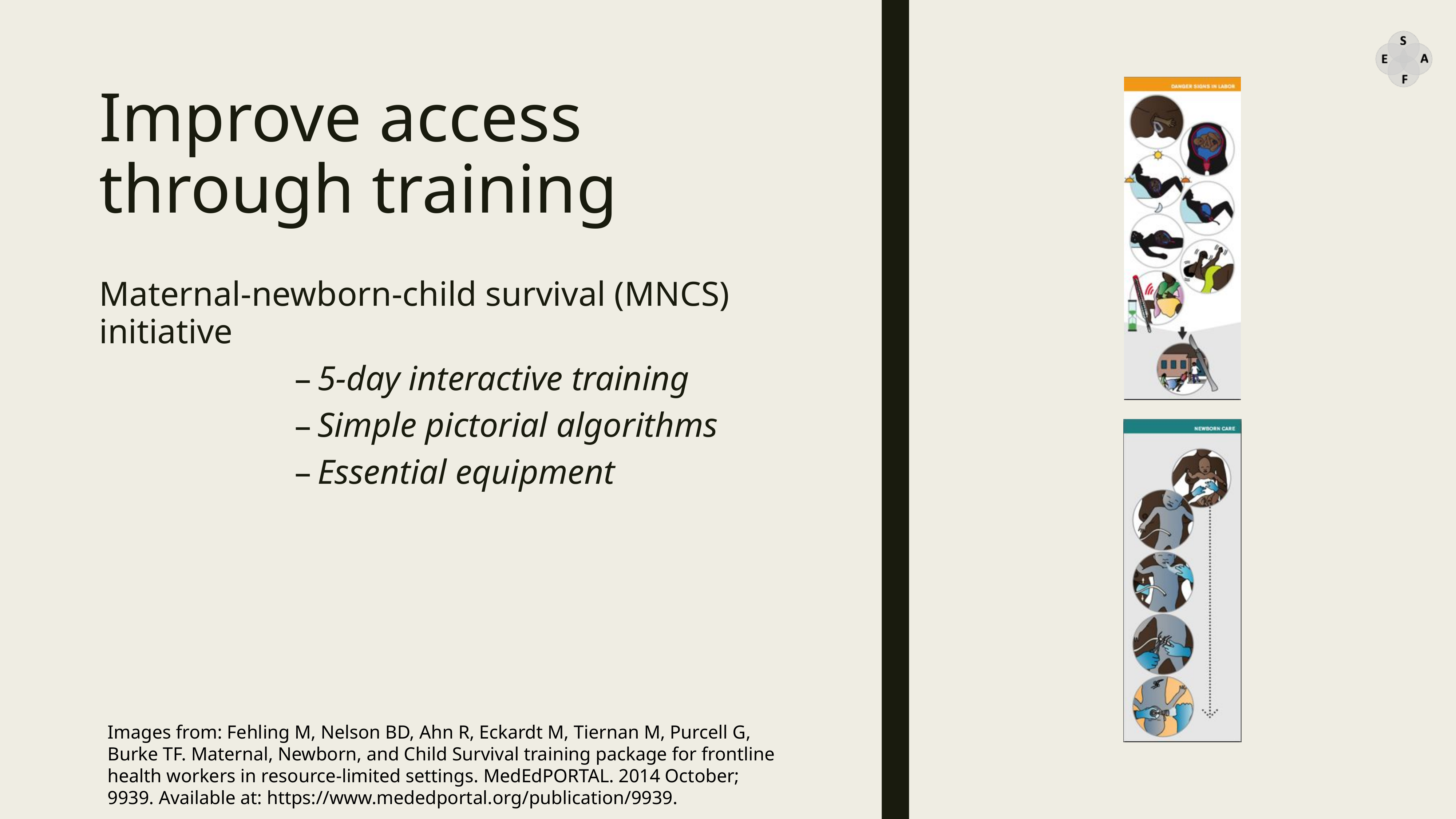

# Improve access through training
Maternal-newborn-child survival (MNCS) initiative
5-day interactive training
Simple pictorial algorithms
Essential equipment
Images from: Fehling M, Nelson BD, Ahn R, Eckardt M, Tiernan M, Purcell G, Burke TF. Maternal, Newborn, and Child Survival training package for frontline health workers in resource-limited settings. MedEdPORTAL. 2014 October; 9939. Available at: https://www.mededportal.org/publication/9939.
21

## Slide 22
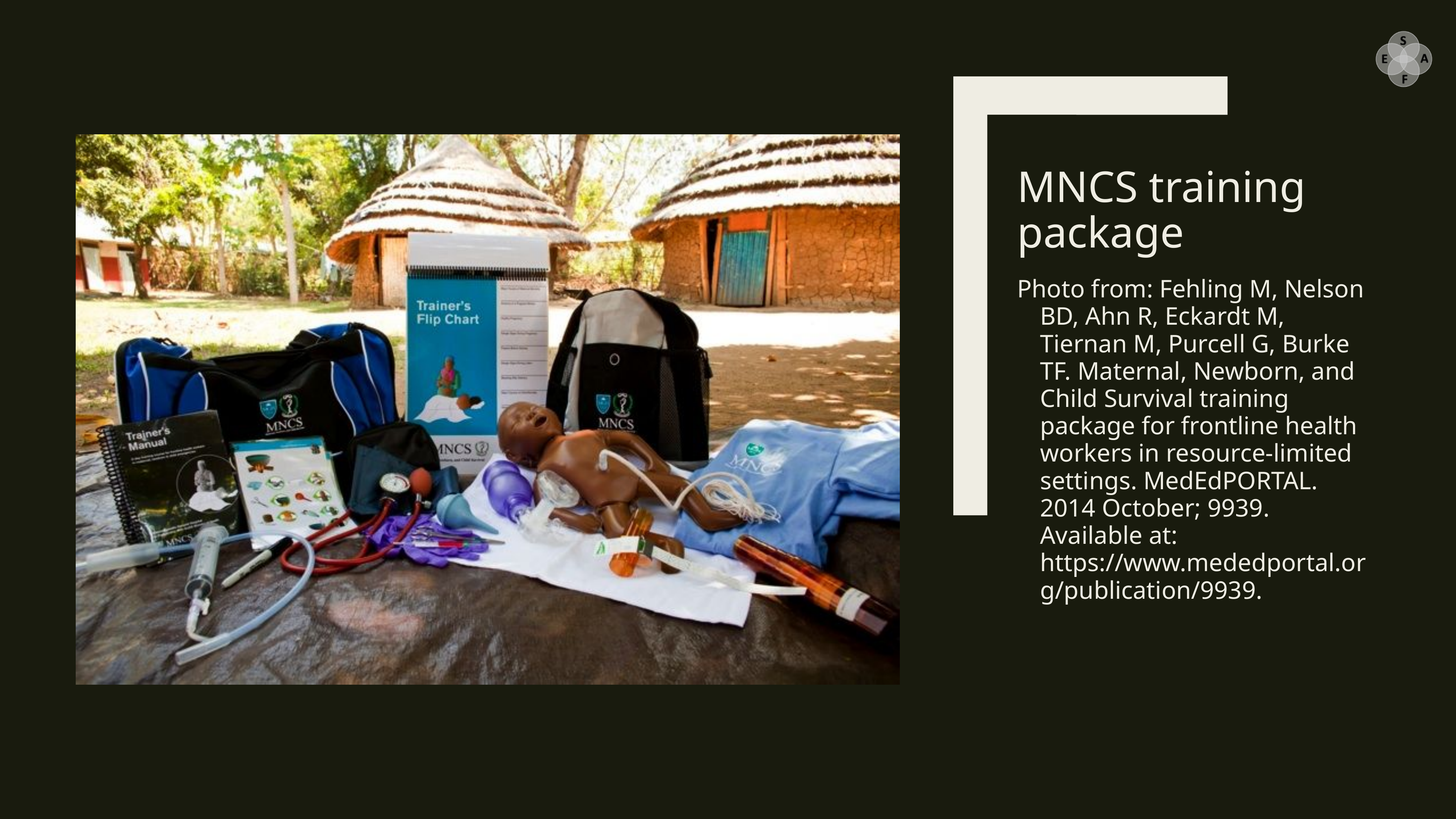

# MNCS training package
Photo from: Fehling M, Nelson BD, Ahn R, Eckardt M, Tiernan M, Purcell G, Burke TF. Maternal, Newborn, and Child Survival training package for frontline health workers in resource-limited settings. MedEdPORTAL. 2014 October; 9939. Available at: https://www.mededportal.org/publication/9939.
22

## Slide 23
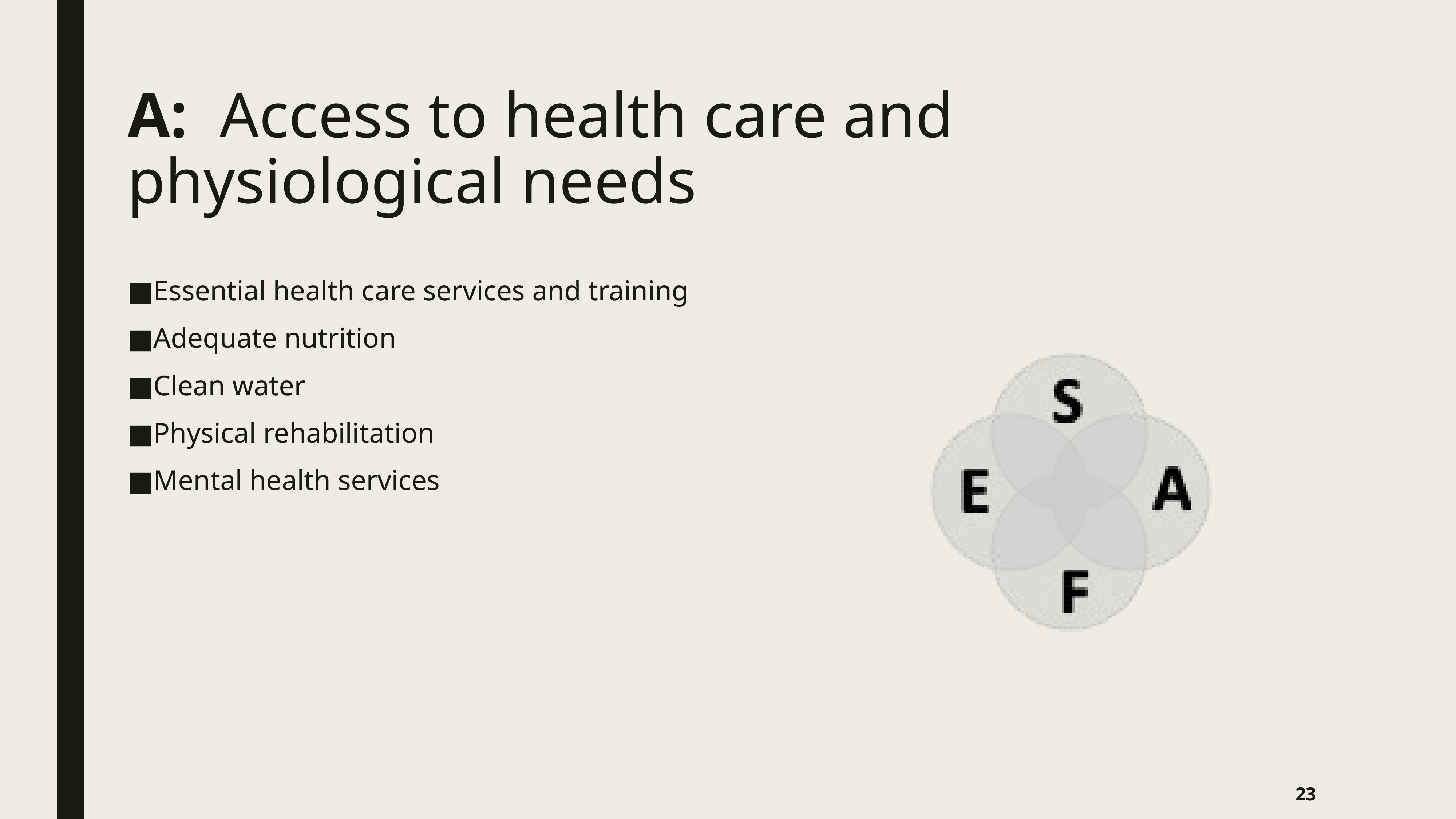

# A: Access to health care and
physiological needs
Essential health care services and training
Adequate nutrition
Clean water
Physical rehabilitation
Mental health services
23

## Slide 24
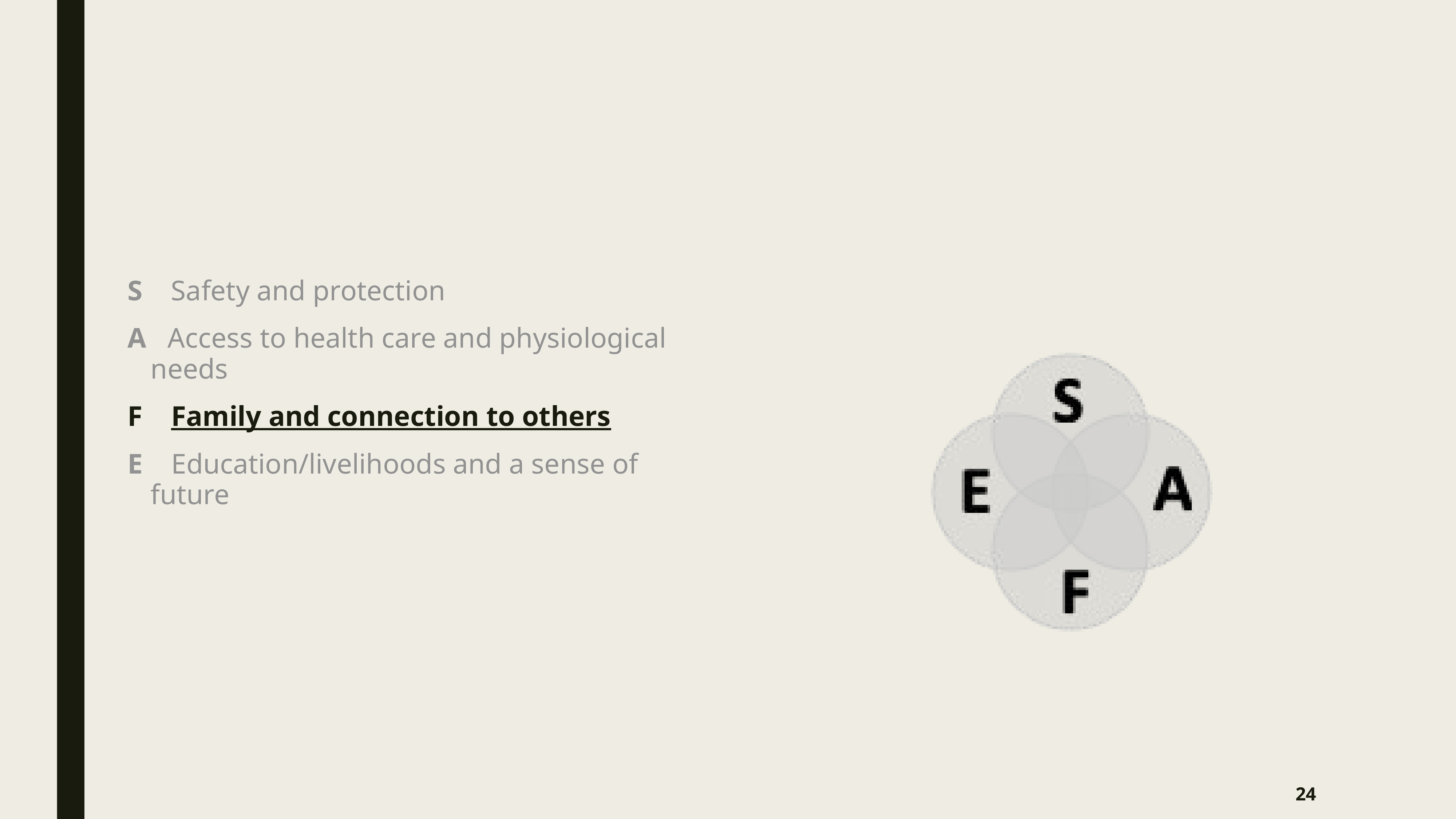

S Safety and protection
A Access to health care and physiological needs
F Family and connection to others
E Education/livelihoods and a sense of future
24

## Slide 25
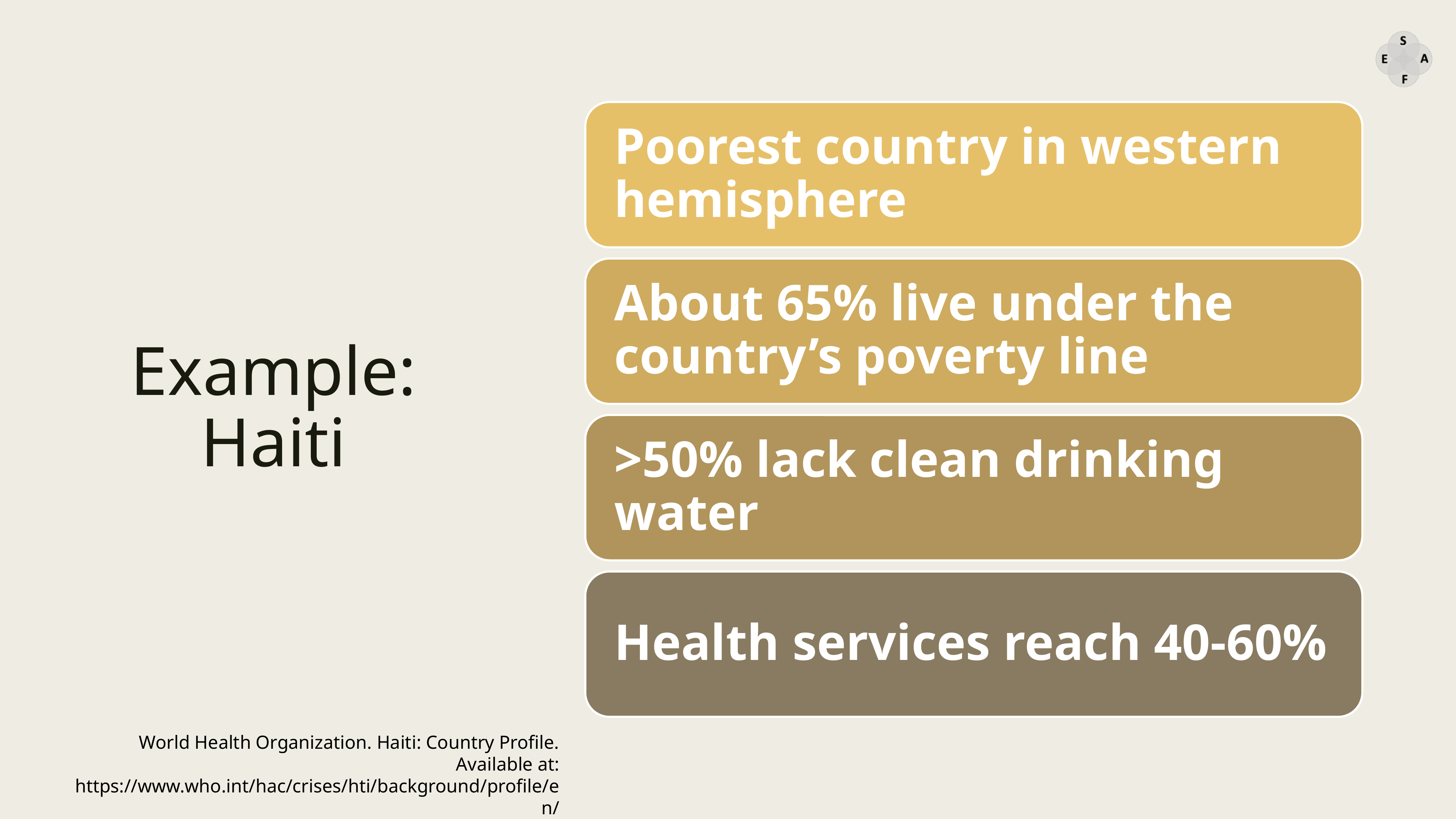

# Example: Haiti
World Health Organization. Haiti: Country Profile. Available at: https://www.who.int/hac/crises/hti/background/profile/en/
25

## Slide 26
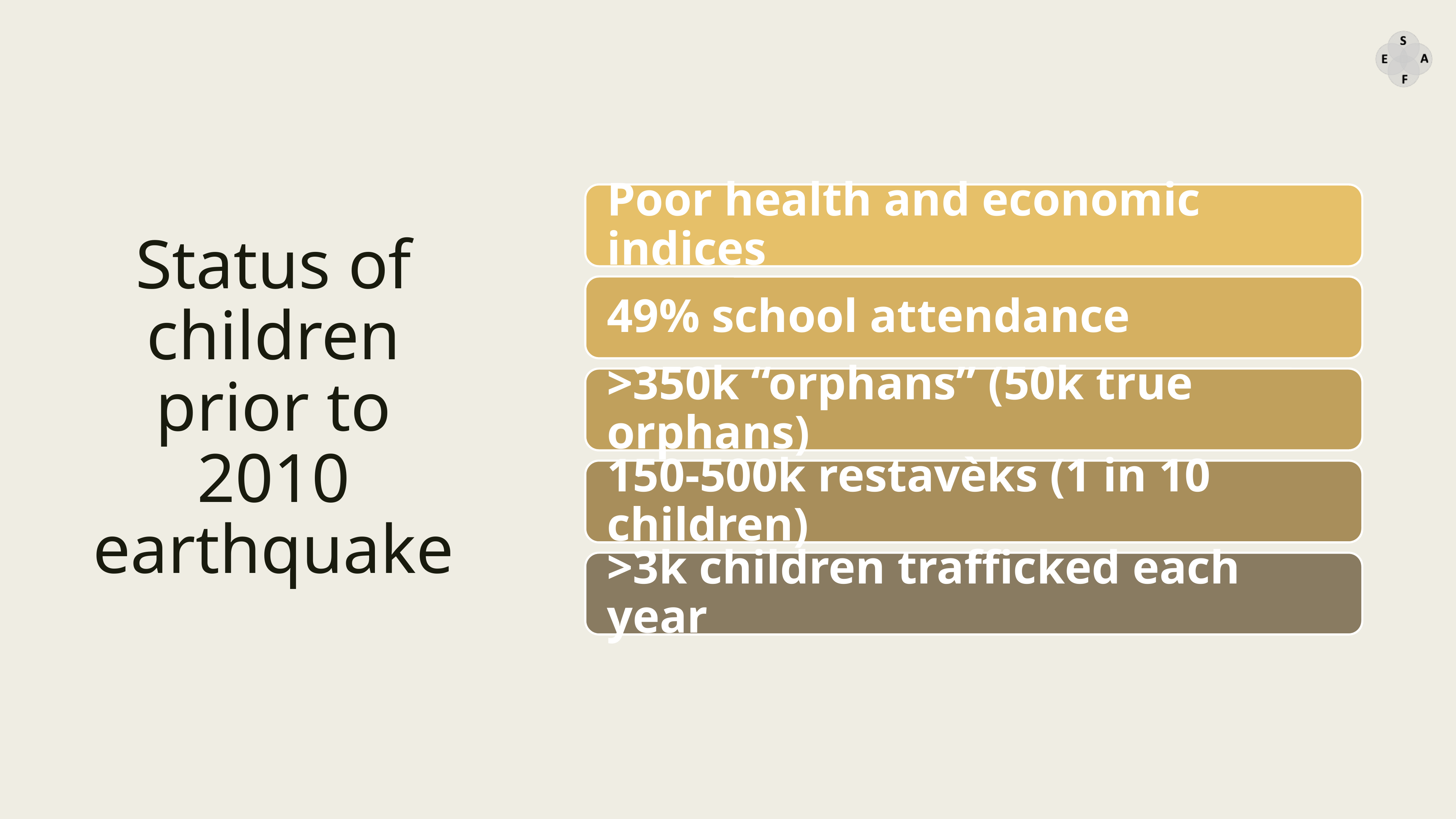

# Status of children prior to 2010 earthquake
26

## Slide 27
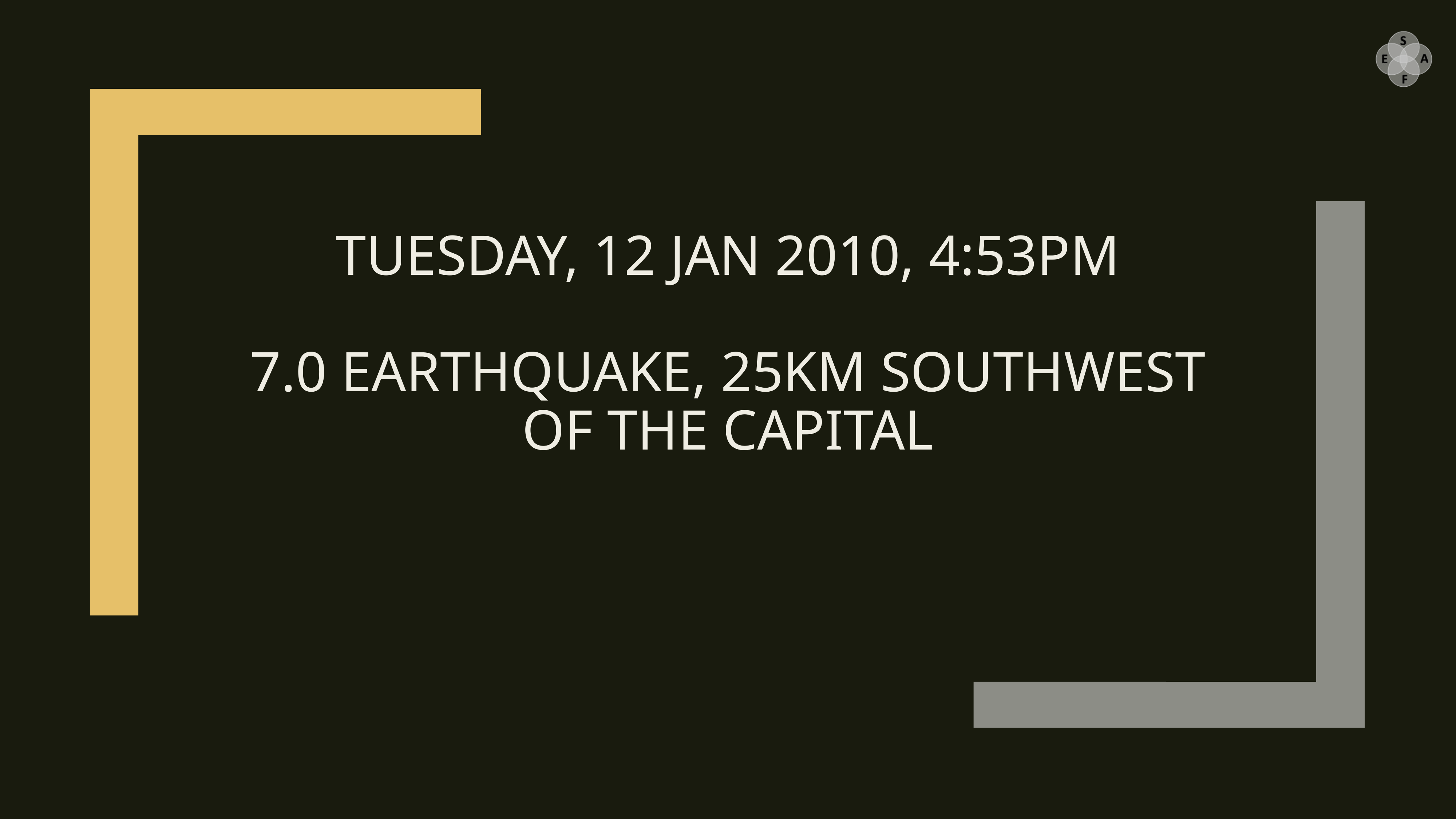

# Tuesday, 12 Jan 2010, 4:53pm 7.0 earthquake, 25km southwest of the capital
27

## Slide 28
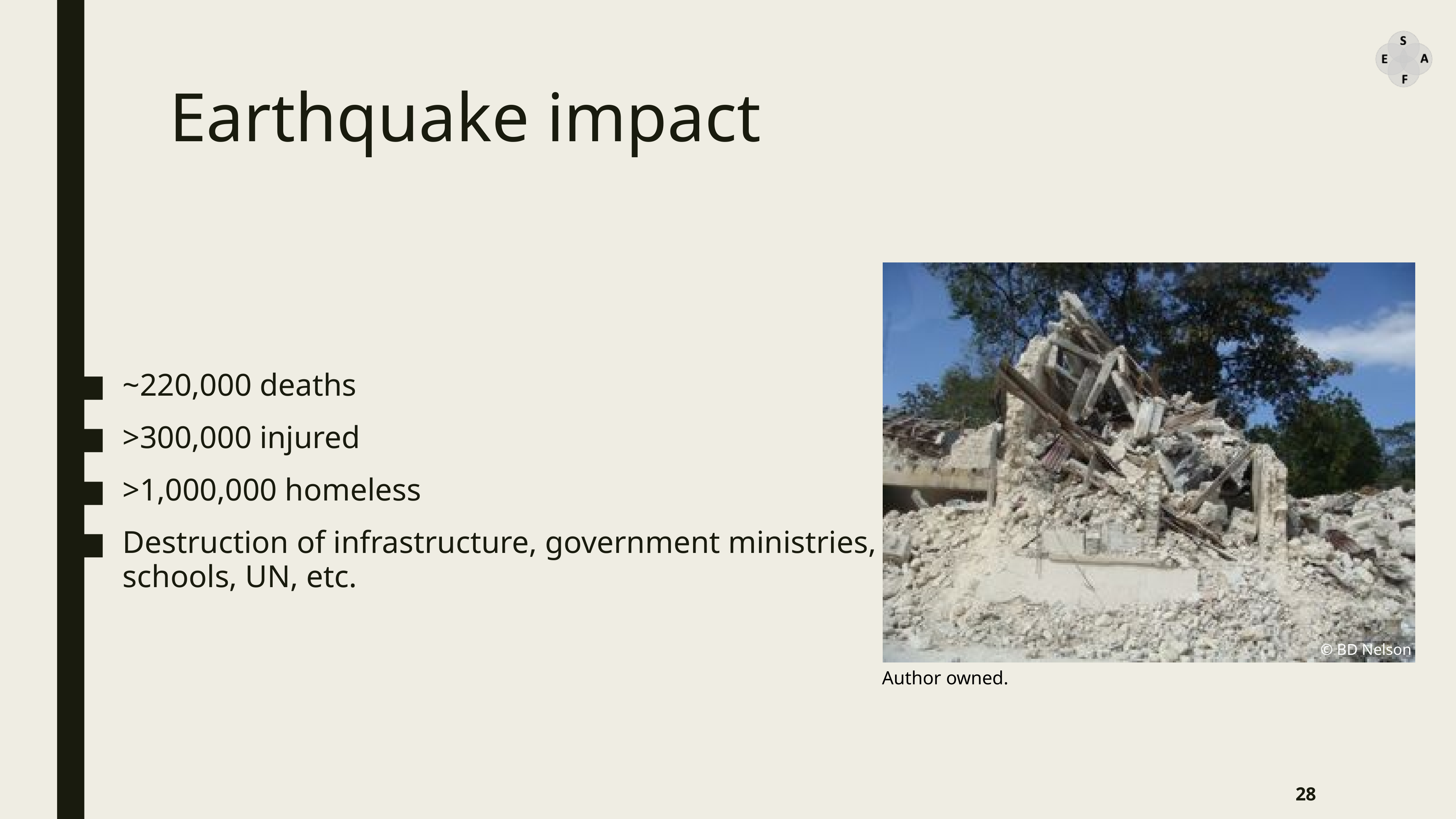

# Earthquake impact
~220,000 deaths
>300,000 injured
>1,000,000 homeless
Destruction of infrastructure, government ministries, schools, UN, etc.
© BD Nelson
Author owned.
28

## Slide 29
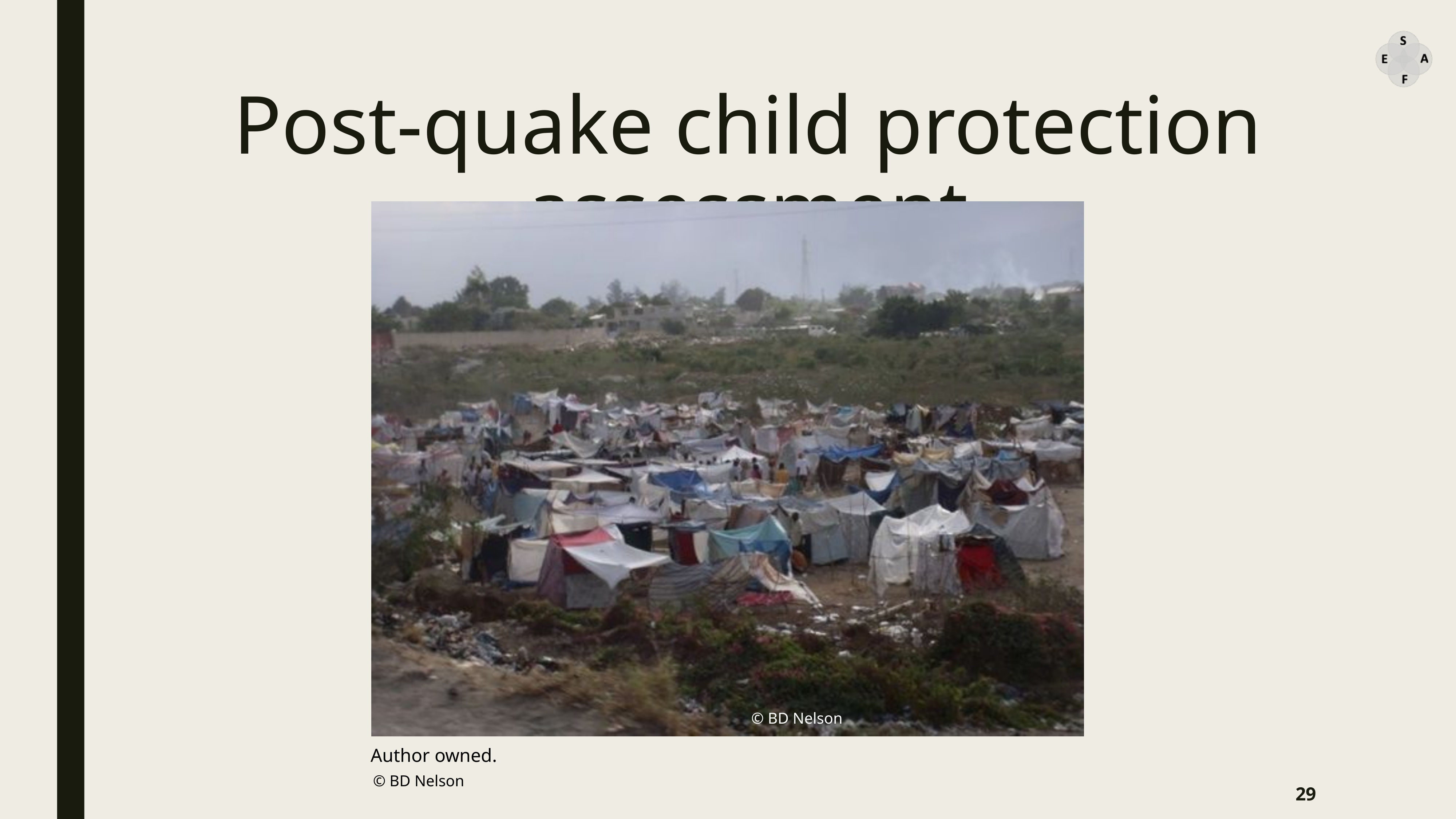

# Post-quake child protection assessment
© BD Nelson
Author owned.
© BD Nelson
29

## Slide 30
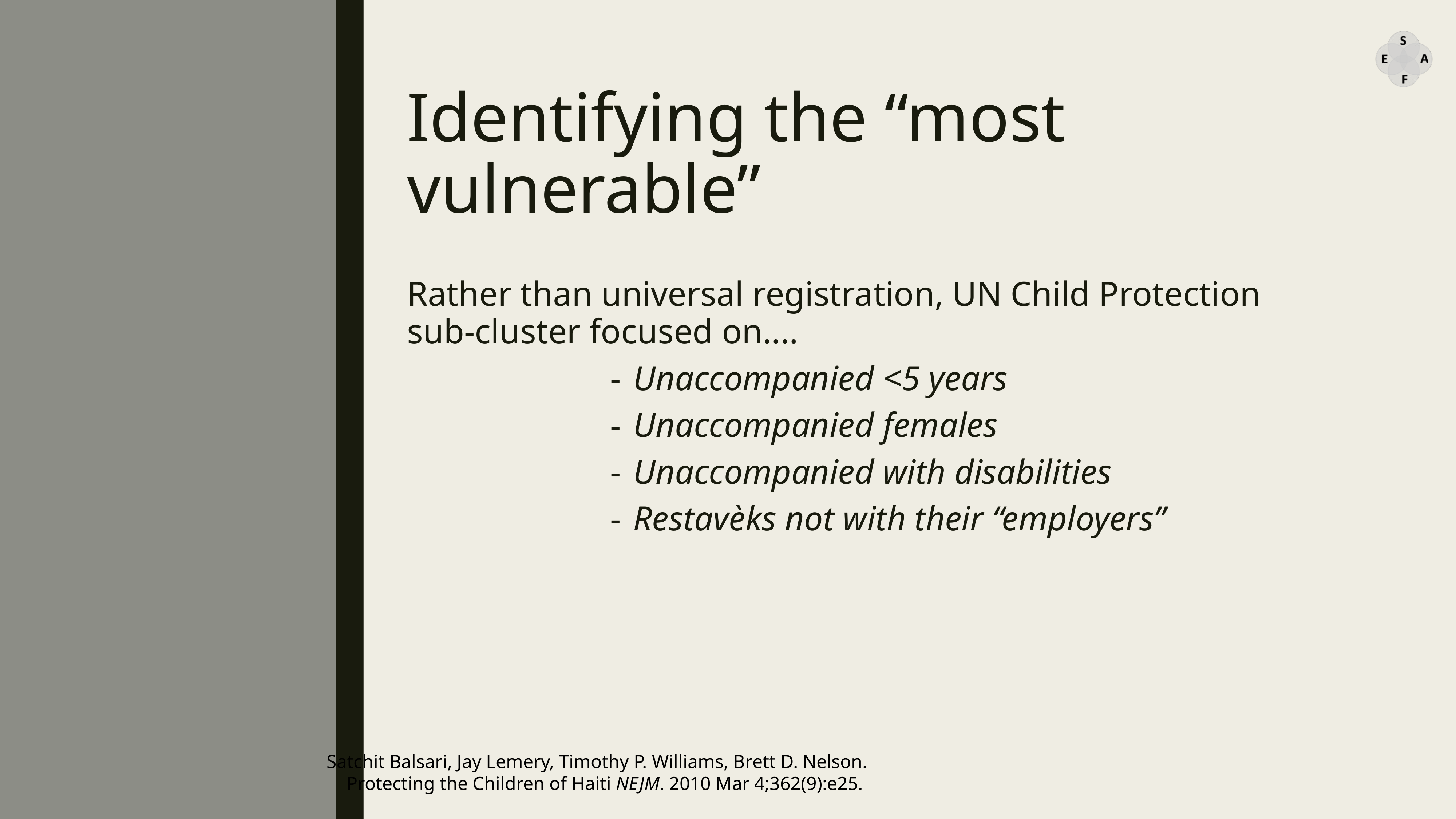

# Identifying the “most vulnerable”
Rather than universal registration, UN Child Protection sub-cluster focused on....
Unaccompanied <5 years
Unaccompanied females
Unaccompanied with disabilities
Restavèks not with their “employers”
Satchit Balsari, Jay Lemery, Timothy P. Williams, Brett D. Nelson. Protecting the Children of Haiti NEJM. 2010 Mar 4;362(9):e25.
30

## Slide 31
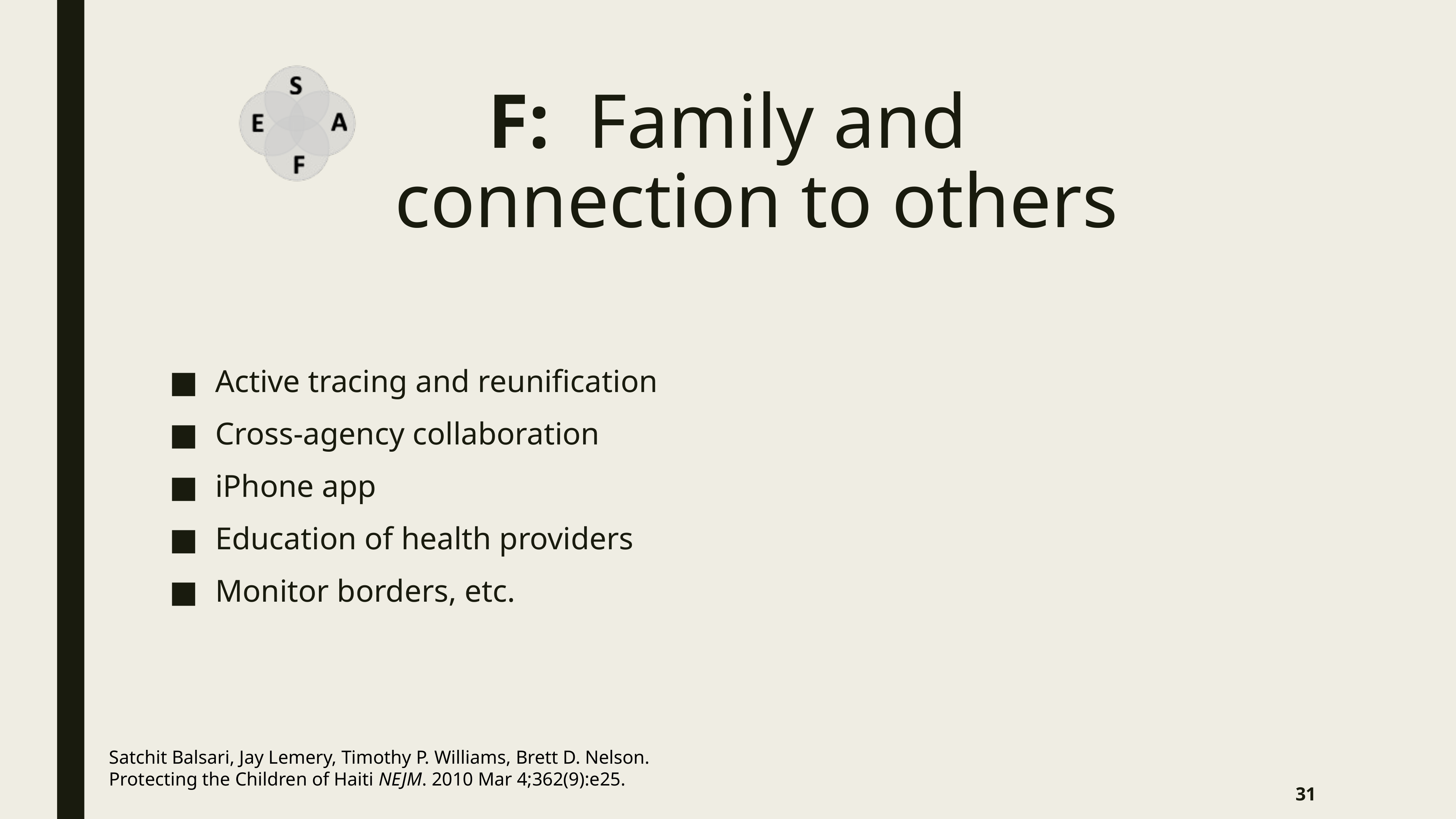

# F: Family and
 connection to others
Active tracing and reunification
Cross-agency collaboration
iPhone app
Education of health providers
Monitor borders, etc.
Satchit Balsari, Jay Lemery, Timothy P. Williams, Brett D. Nelson. Protecting the Children of Haiti NEJM. 2010 Mar 4;362(9):e25.
31

## Slide 32
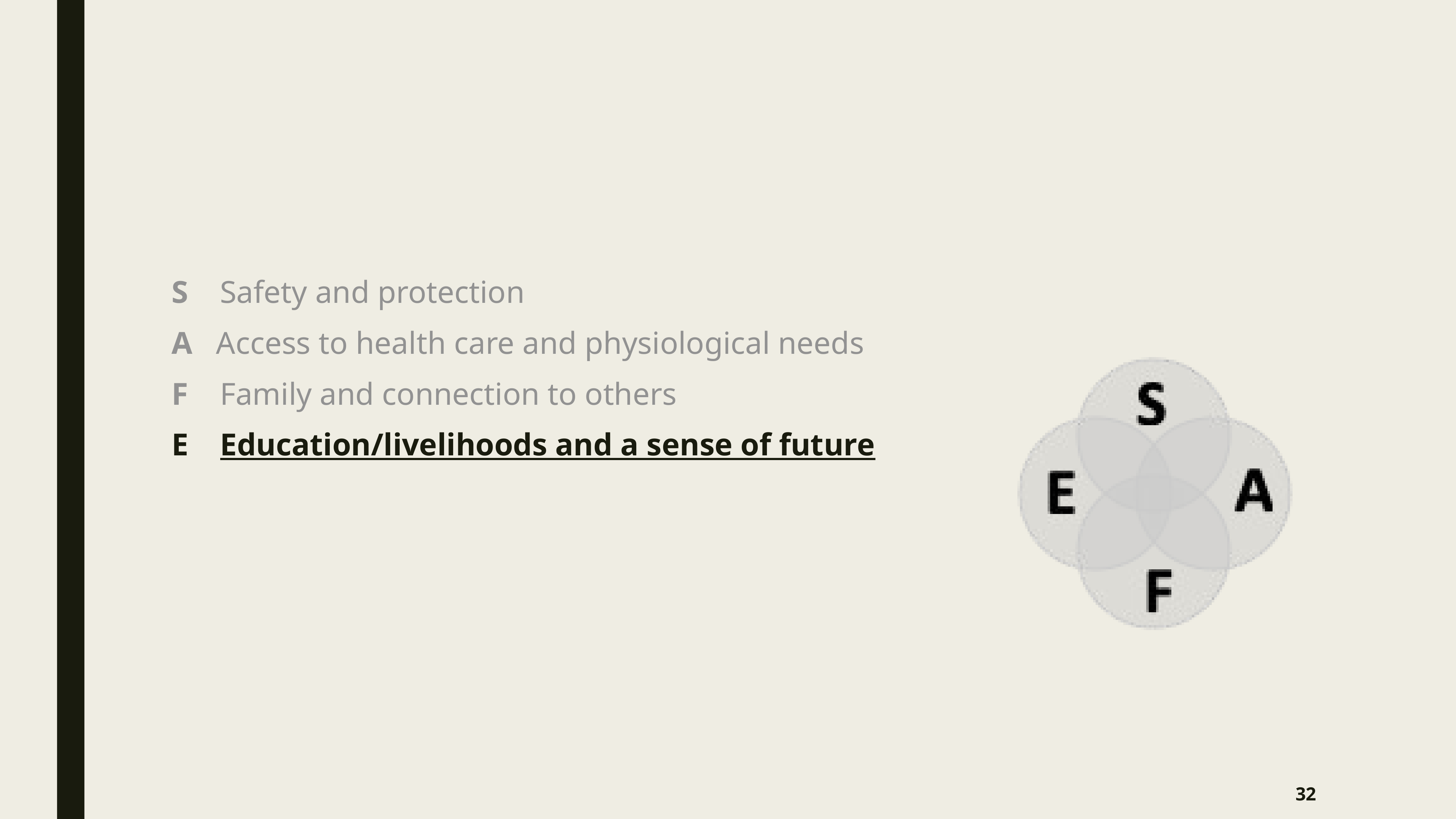

S Safety and protection
A Access to health care and physiological needs
F Family and connection to others
E Education/livelihoods and a sense of future
32

## Slide 33
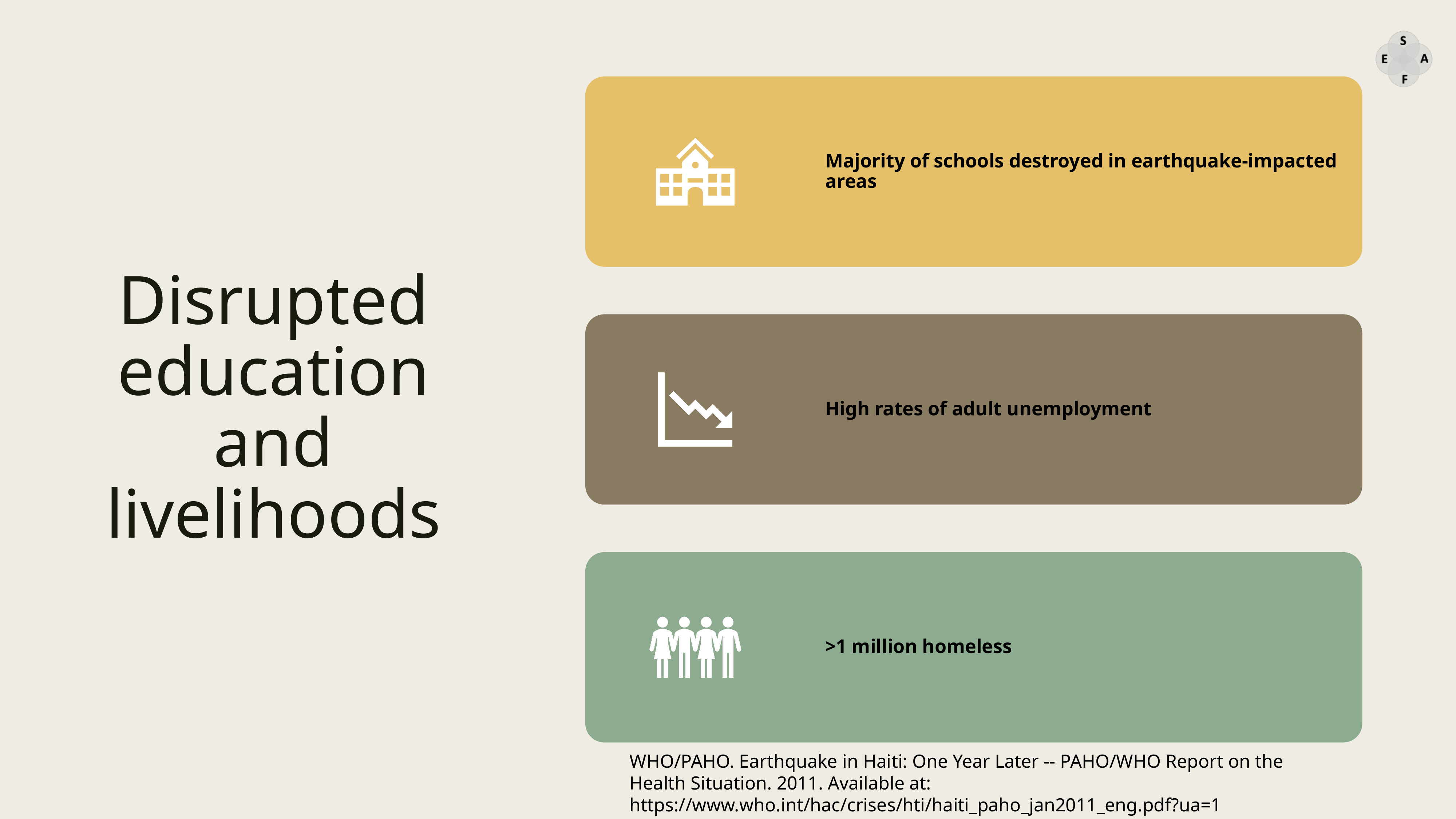

# Disrupted education and livelihoods
WHO/PAHO. Earthquake in Haiti: One Year Later -- PAHO/WHO Report on the Health Situation. 2011. Available at: https://www.who.int/hac/crises/hti/haiti_paho_jan2011_eng.pdf?ua=1
33

## Slide 34
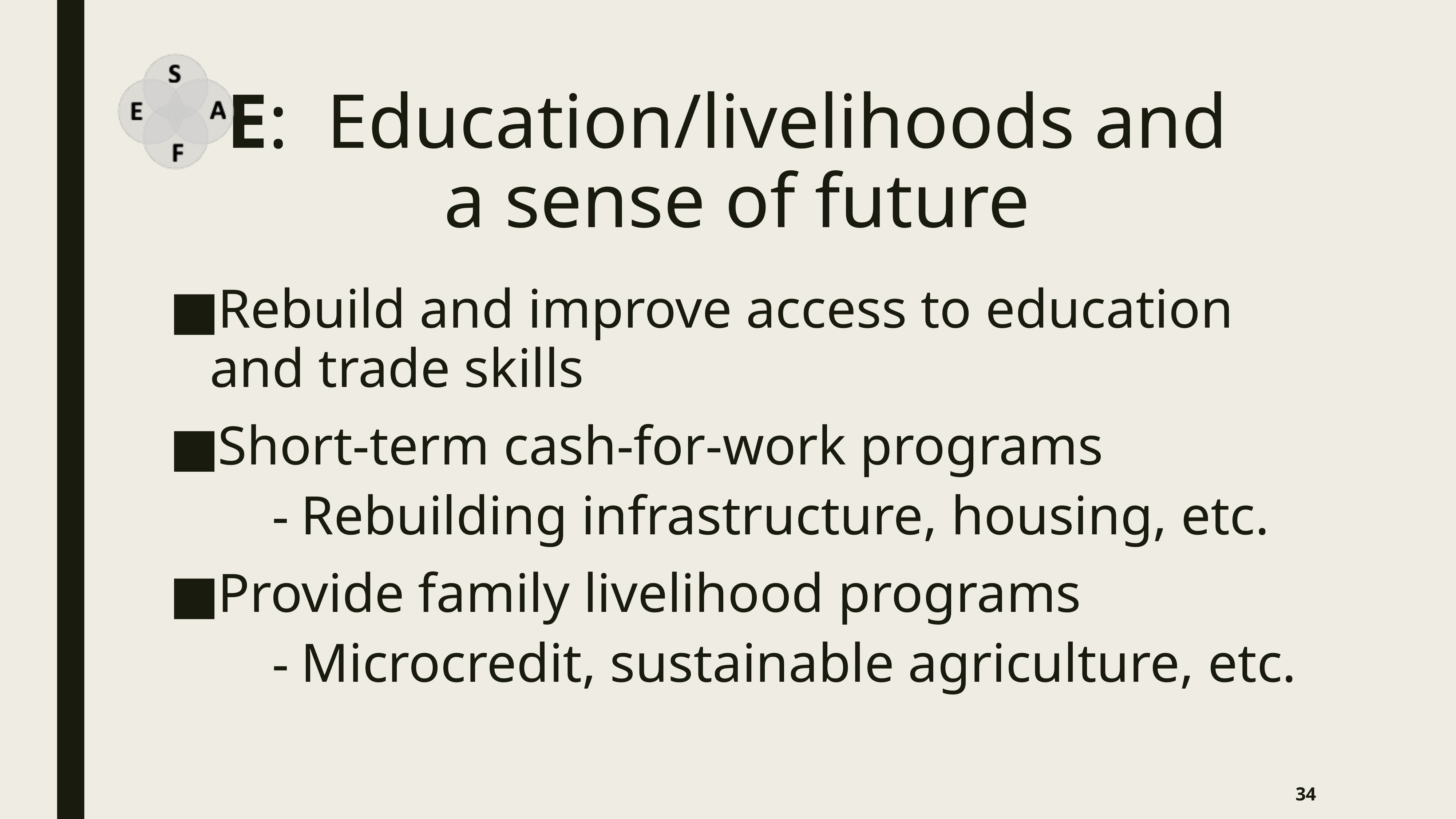

# E: Education/livelihoods and
a sense of future
Rebuild and improve access to education and trade skills
Short-term cash-for-work programs
Rebuilding infrastructure, housing, etc.
Provide family livelihood programs
Microcredit, sustainable agriculture, etc.
34

## Slide 35
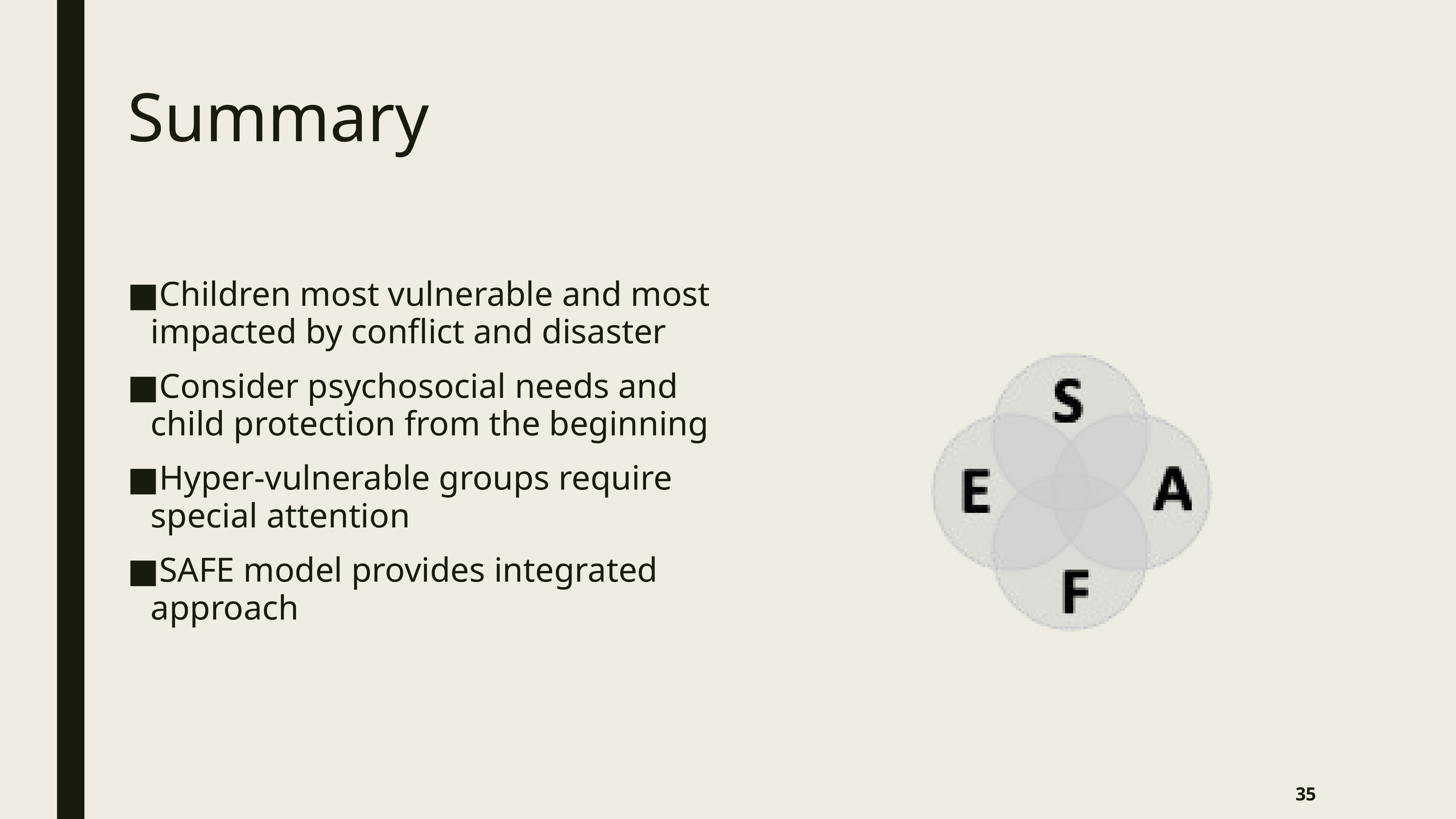

# Summary
Children most vulnerable and most impacted by conflict and disaster
Consider psychosocial needs and child protection from the beginning
Hyper-vulnerable groups require special attention
SAFE model provides integrated approach
35

## Slide 36
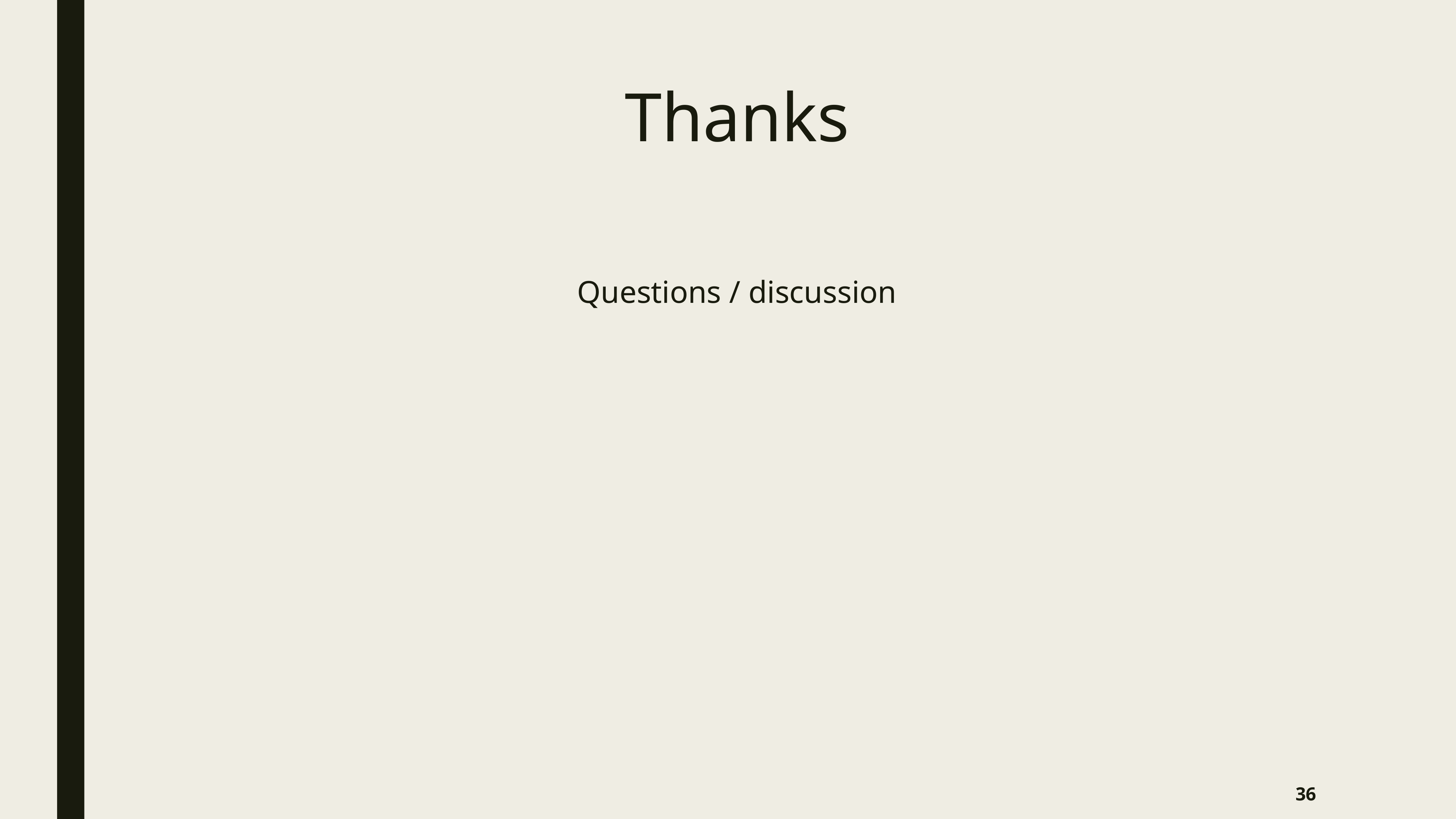

# Thanks
Questions / discussion
36
