# Supplementary material for: Pediatric Refugee Health Care Delivery in the Community Setting: An Educational Workshop for Multidisciplinary Family-Centered Care During Resettlement
Source: MedEdPORTAL. 2020 Nov 3;16:10988. doi: 10.15766/mep_2374-8265.10988 (PMC7666829; doi:10.15766/mep_2374-8265.10988)
Supplement: Supplementary file 1 — Agenda.docxPresentation 1 Intro to Refugees.pptxPresentation 2 Health Screening.pptxCases.docxPresentation 3 Trauma-Informed Care.pptxPresentation 4 Refugee Health Advocacy.pptxRefugee Workshop Evaluation.docx [file mep_2374-8265.10988-s001.zip › F. Presentation 4 Refugee Health Advocacy.pptx]

## Slide 1
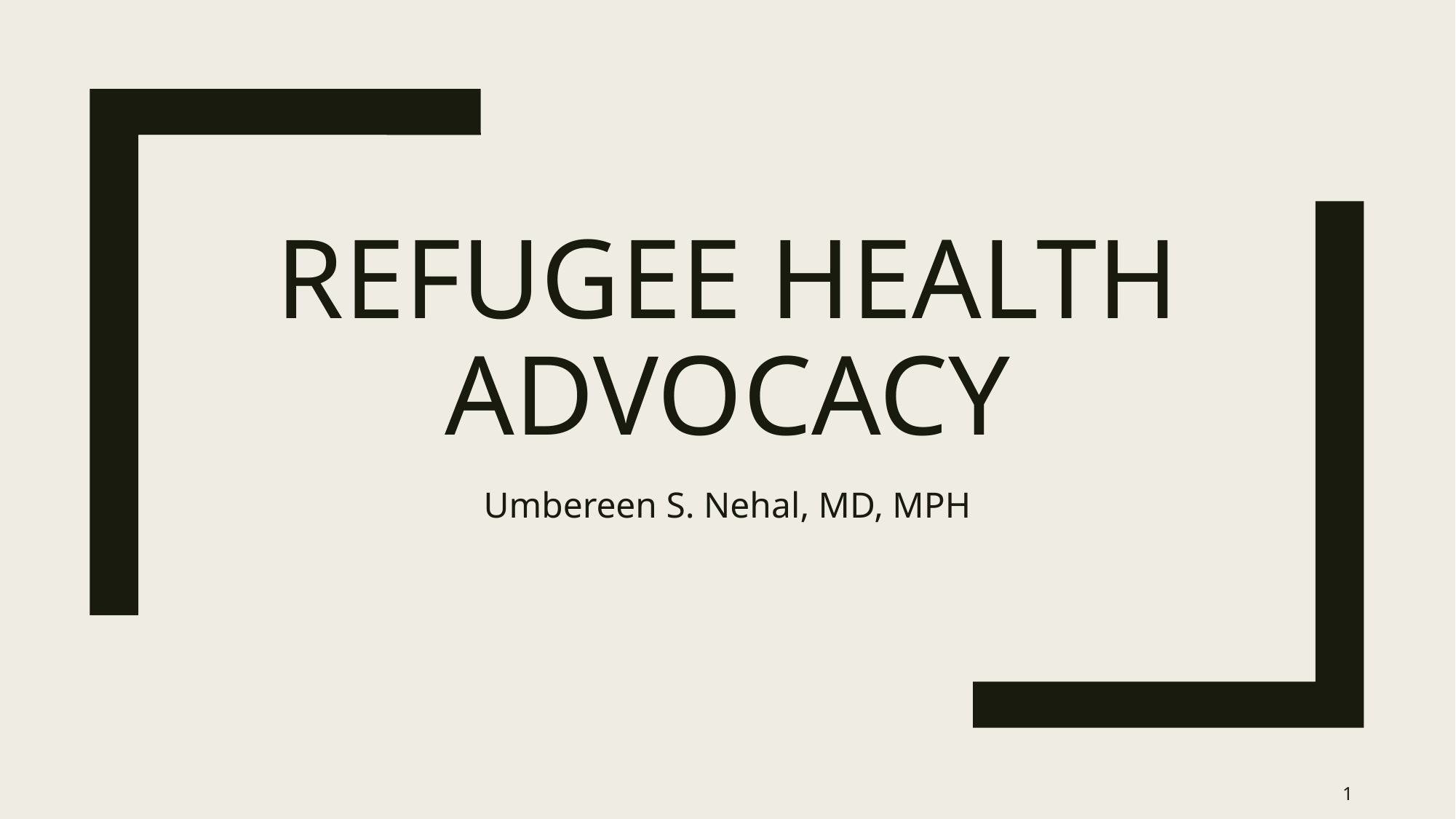

# Refugee Health Advocacy
Umbereen S. Nehal, MD, MPH
1

## Slide 2
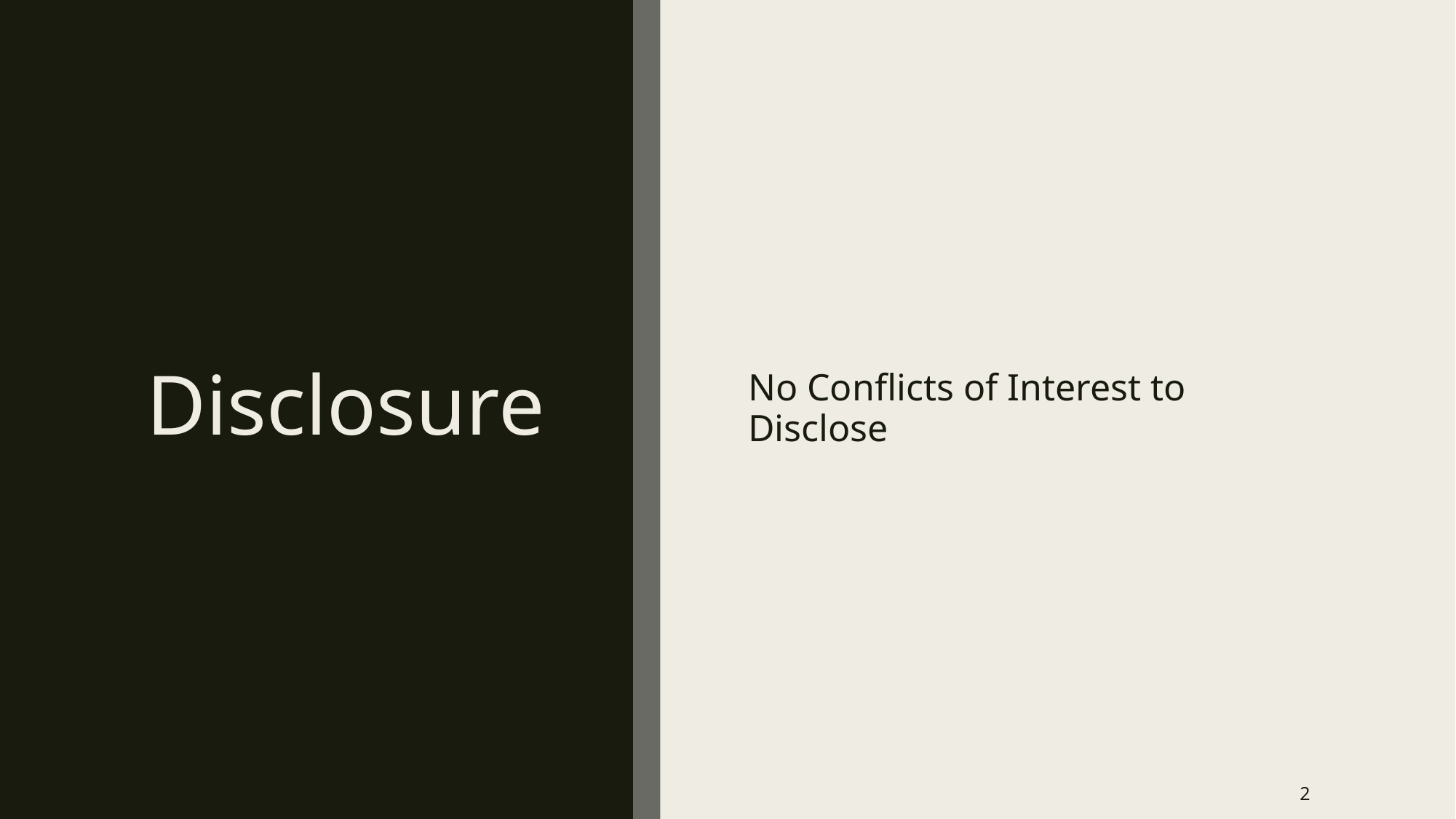

# Disclosure
No Conflicts of Interest to Disclose
2

## Slide 3
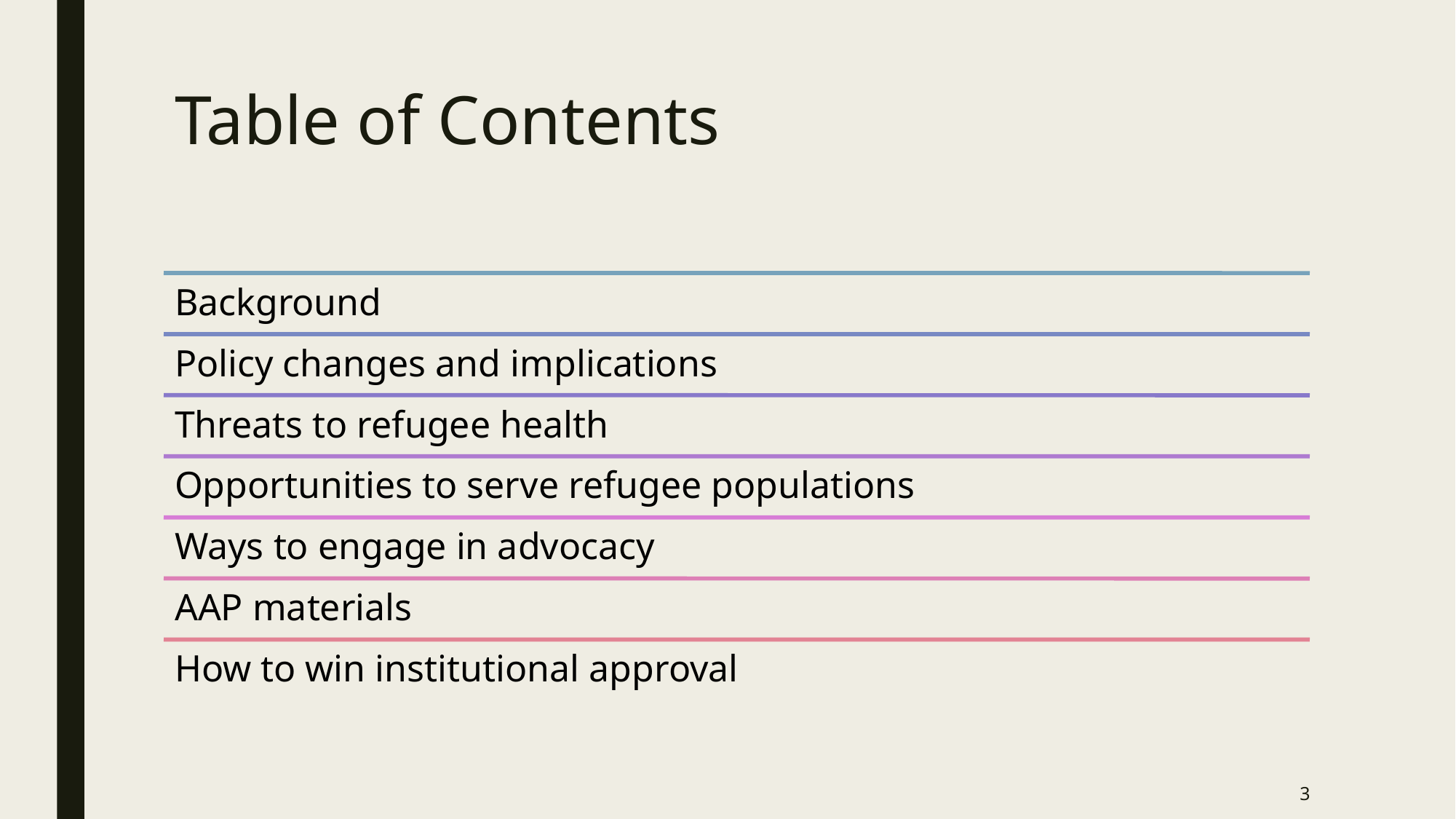

# Table of Contents
3

## Slide 4
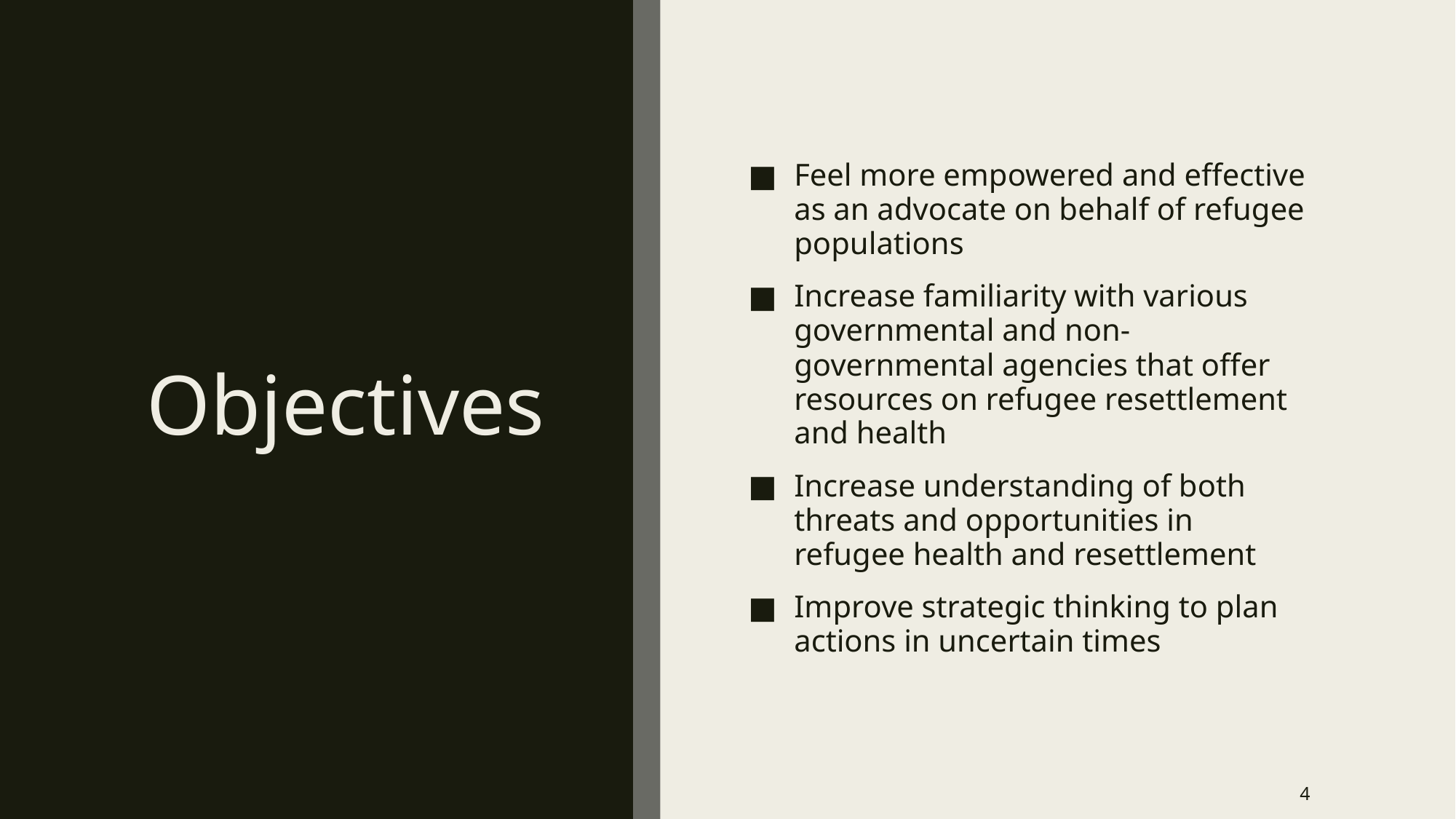

# Objectives
Feel more empowered and effective as an advocate on behalf of refugee populations
Increase familiarity with various governmental and non-governmental agencies that offer resources on refugee resettlement and health
Increase understanding of both threats and opportunities in refugee health and resettlement
Improve strategic thinking to plan actions in uncertain times
4

## Slide 5
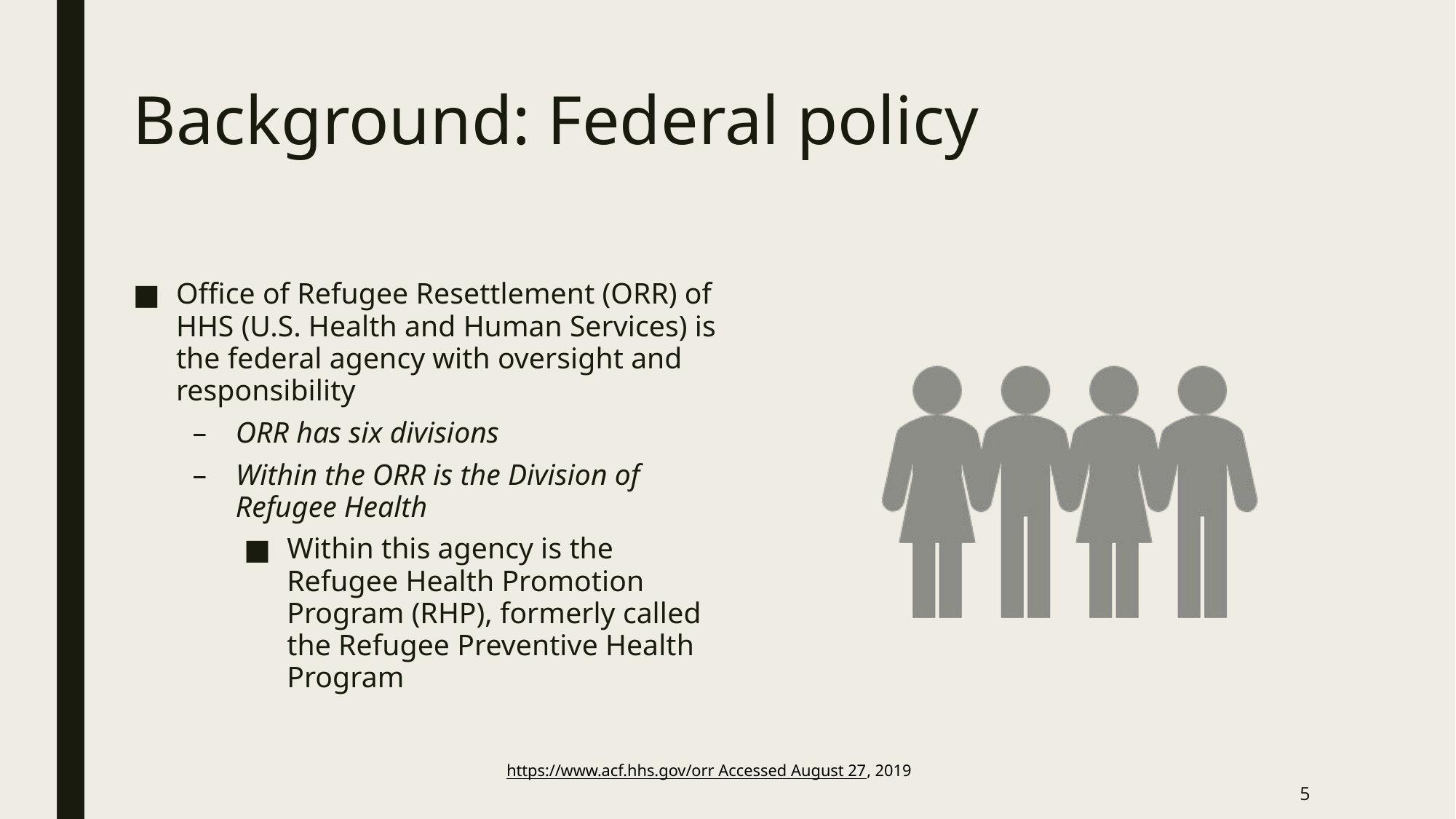

# Background: Federal policy
Office of Refugee Resettlement (ORR) of HHS (U.S. Health and Human Services) is the federal agency with oversight and responsibility
ORR has six divisions
Within the ORR is the Division of Refugee Health
Within this agency is the Refugee Health Promotion Program (RHP), formerly called the Refugee Preventive Health Program
https://www.acf.hhs.gov/orr Accessed August 27, 2019
5

## Slide 6
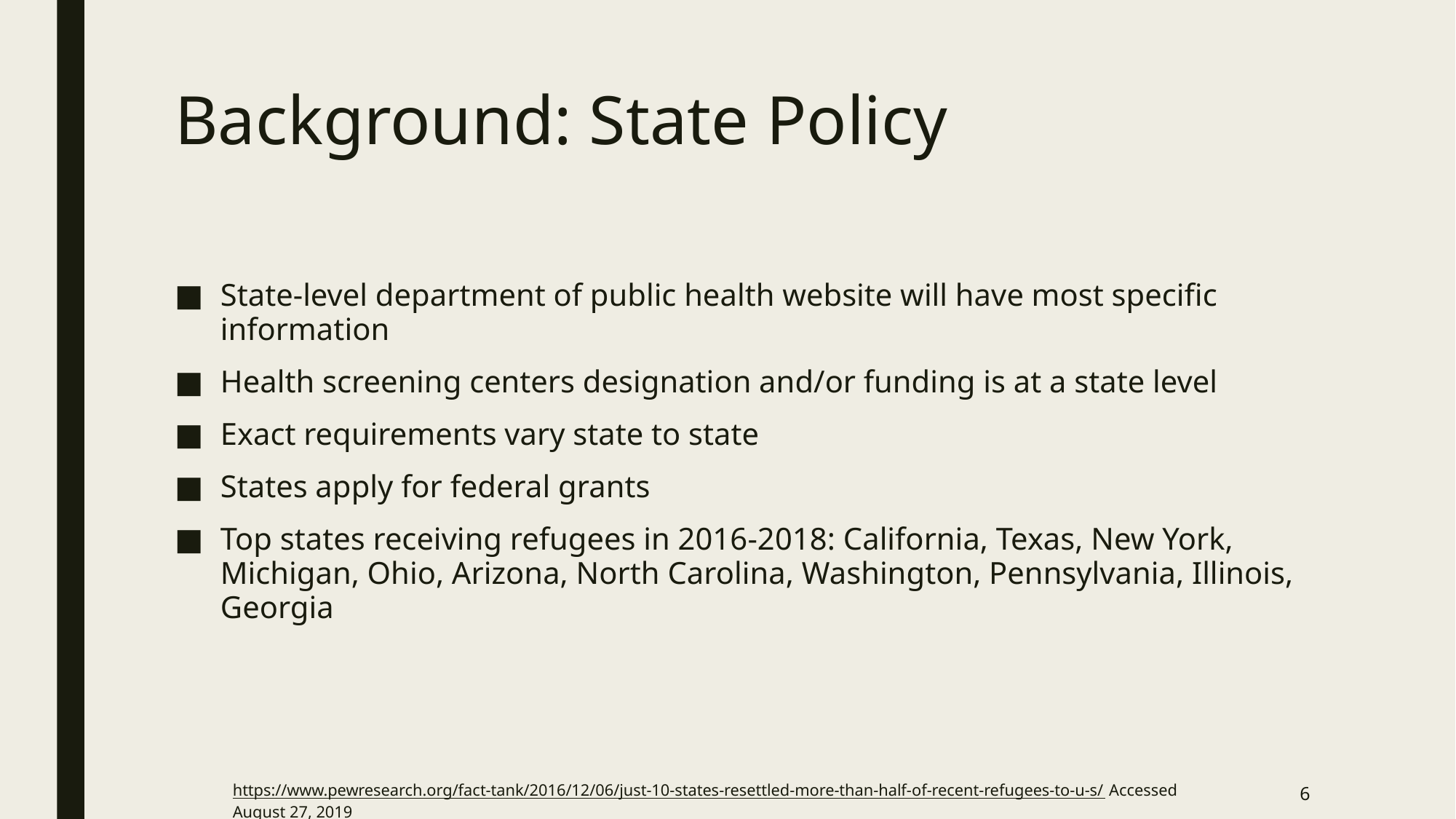

# Background: State Policy
State-level department of public health website will have most specific information
Health screening centers designation and/or funding is at a state level
Exact requirements vary state to state
States apply for federal grants
Top states receiving refugees in 2016-2018: California, Texas, New York, Michigan, Ohio, Arizona, North Carolina, Washington, Pennsylvania, Illinois, Georgia
6
https://www.pewresearch.org/fact-tank/2016/12/06/just-10-states-resettled-more-than-half-of-recent-refugees-to-u-s/ Accessed August 27, 2019

## Slide 7
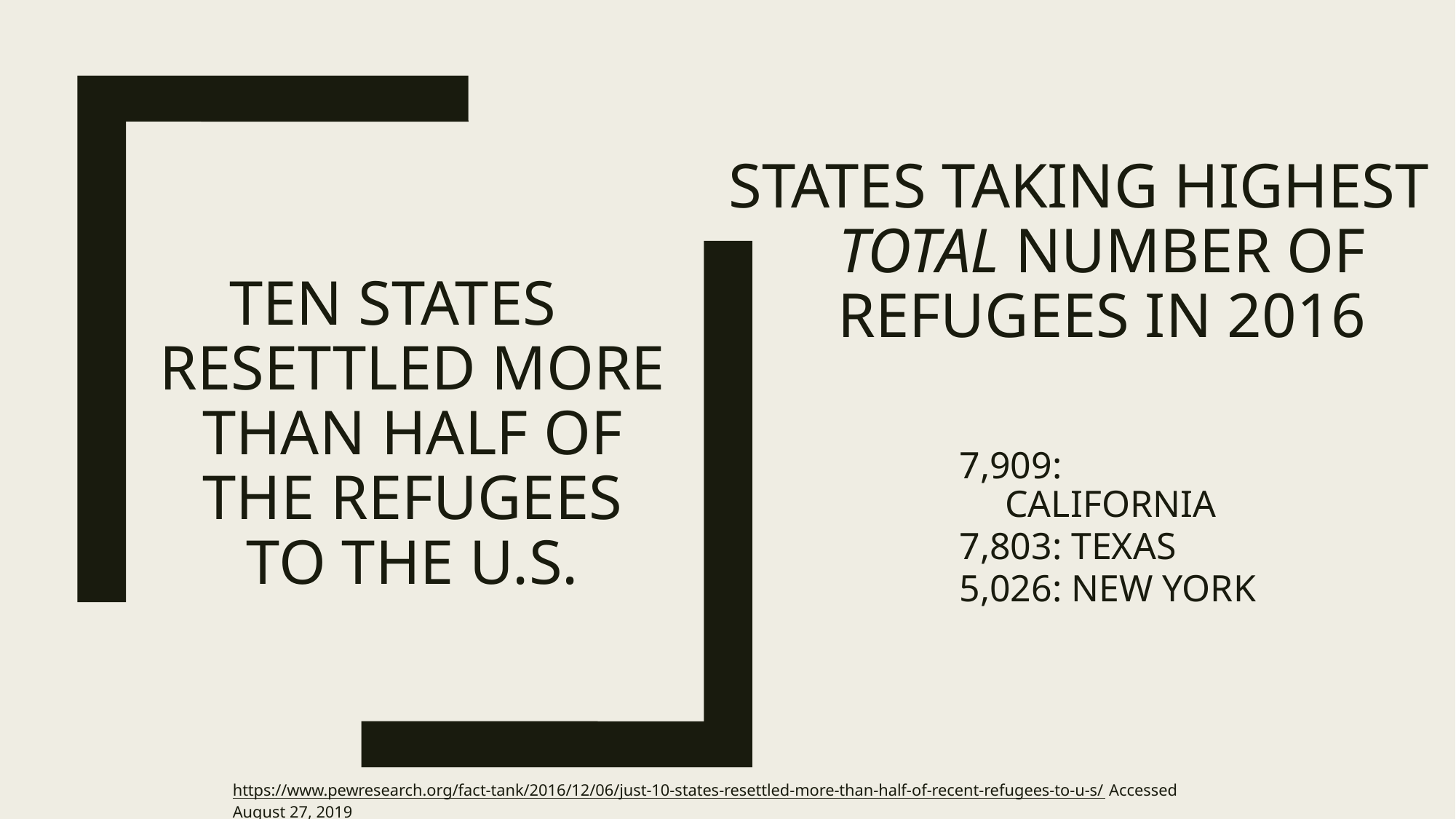

States taking highest Total number of refugees in 2016
Ten States resettled More than half of the refugees to the U.s.
7,909: California
7,803: Texas
5,026: New York
7
https://www.pewresearch.org/fact-tank/2016/12/06/just-10-states-resettled-more-than-half-of-recent-refugees-to-u-s/ Accessed August 27, 2019

## Slide 8
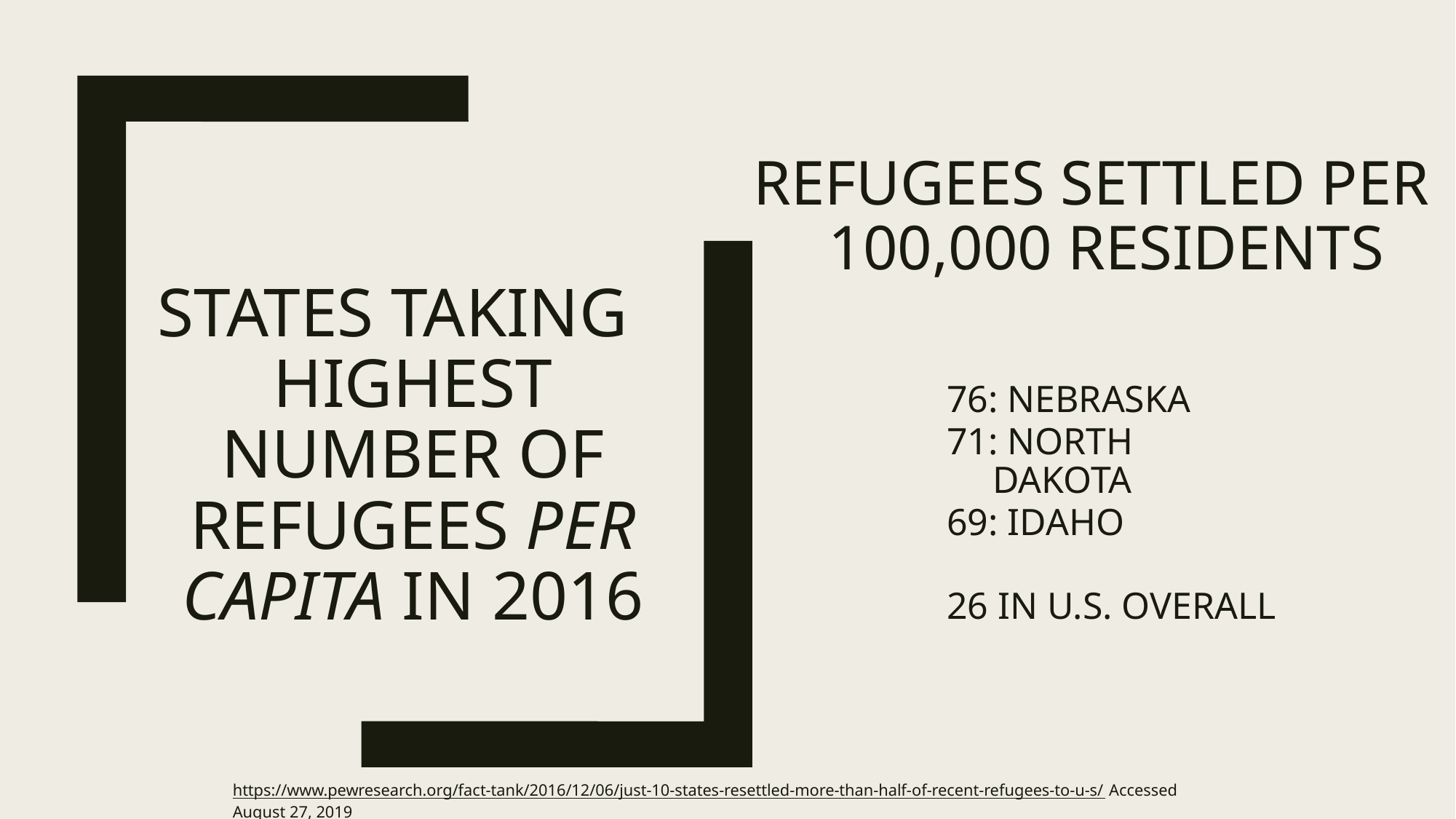

Refugees Settled per 100,000 Residents
States taking highest number of refugees per capita in 2016
76: Nebraska
71: North Dakota
69: Idaho
26 in U.S. overall
8
https://www.pewresearch.org/fact-tank/2016/12/06/just-10-states-resettled-more-than-half-of-recent-refugees-to-u-s/ Accessed August 27, 2019

## Slide 9
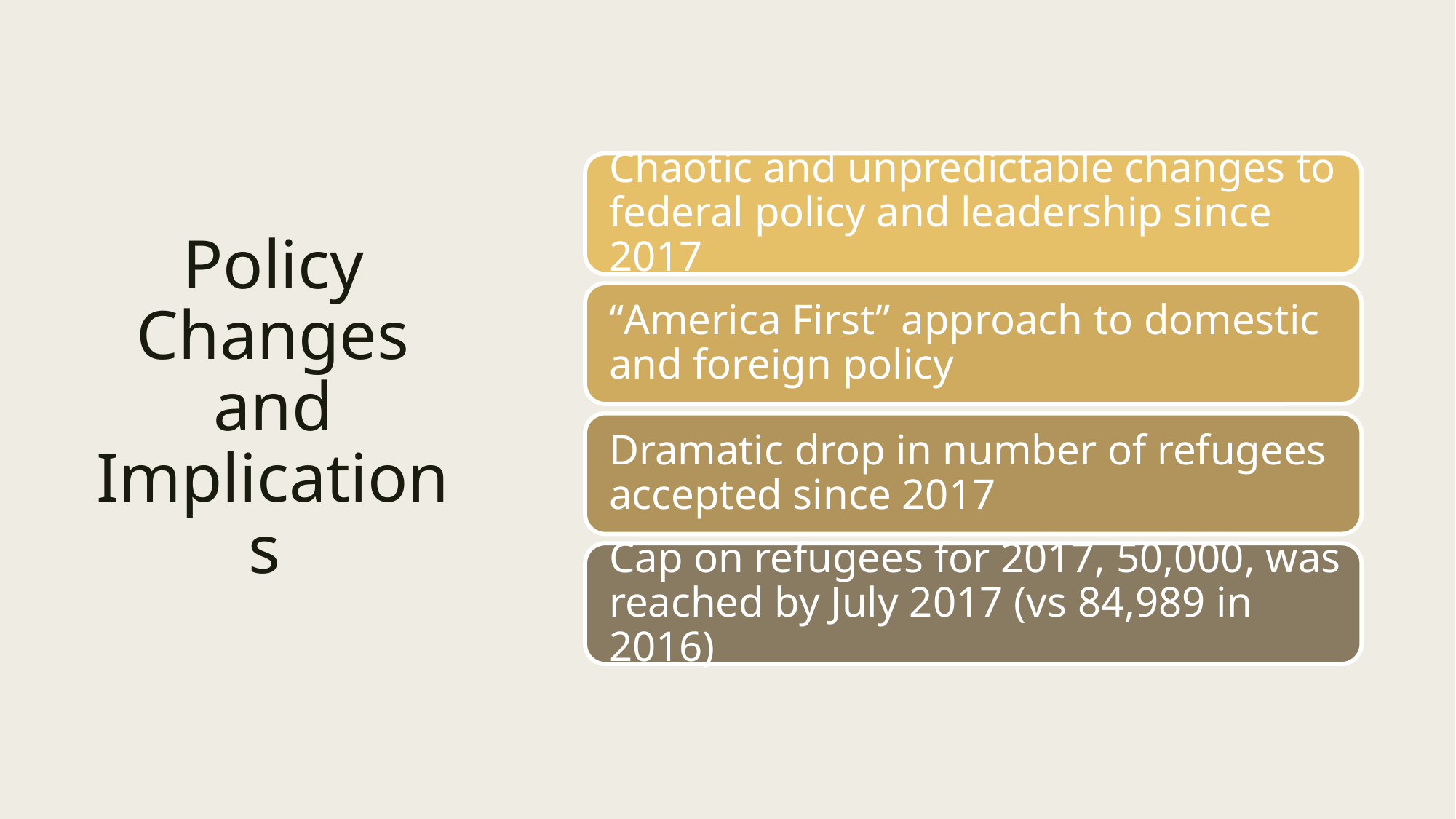

# Policy Changes and Implications
9

## Slide 10
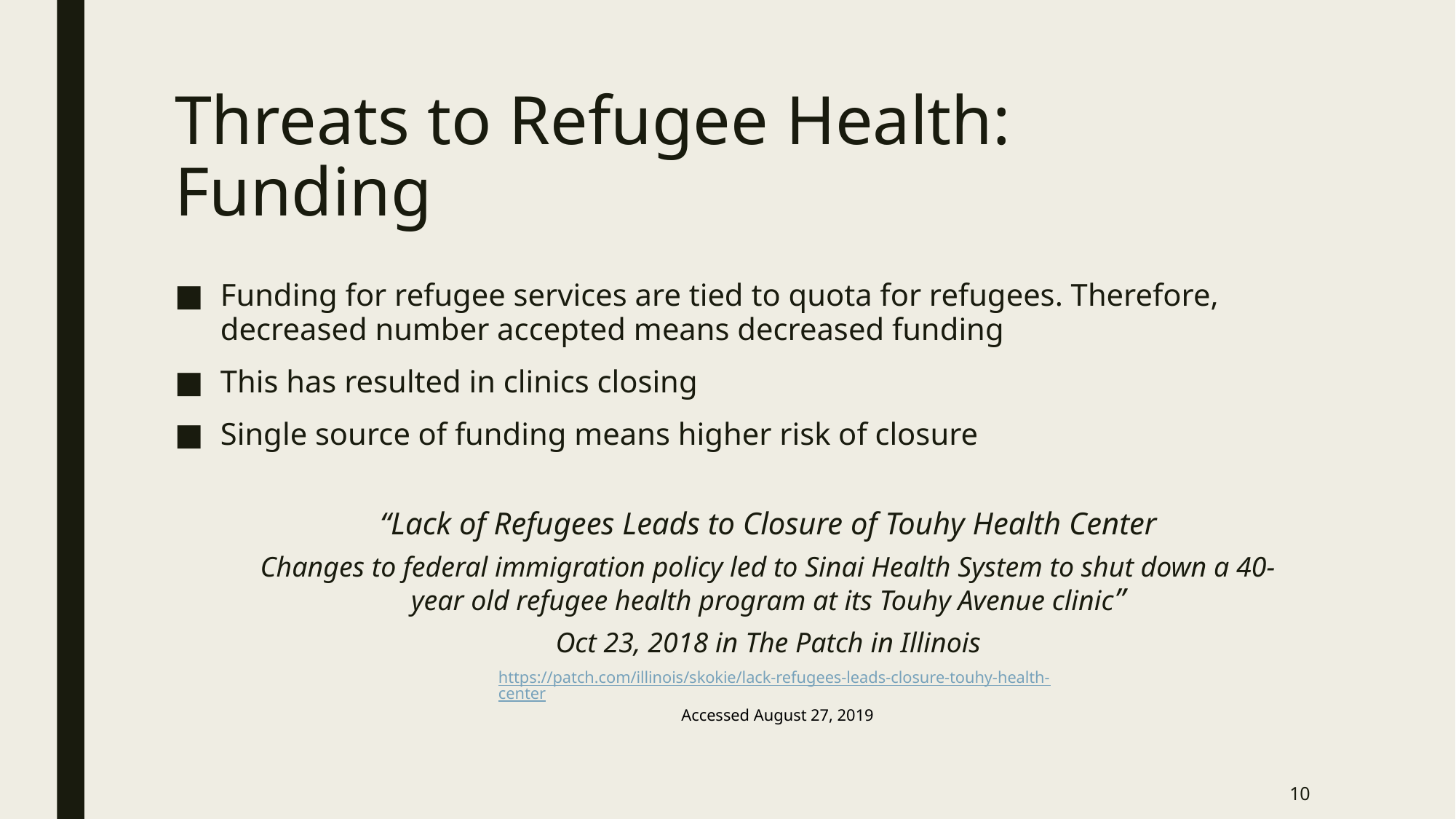

# Threats to Refugee Health: Funding
Funding for refugee services are tied to quota for refugees. Therefore, decreased number accepted means decreased funding
This has resulted in clinics closing
Single source of funding means higher risk of closure
“Lack of Refugees Leads to Closure of Touhy Health Center
Changes to federal immigration policy led to Sinai Health System to shut down a 40-year old refugee health program at its Touhy Avenue clinic”
Oct 23, 2018 in The Patch in Illinois
https://patch.com/illinois/skokie/lack-refugees-leads-closure-touhy-health-center
Accessed August 27, 2019
10

## Slide 11
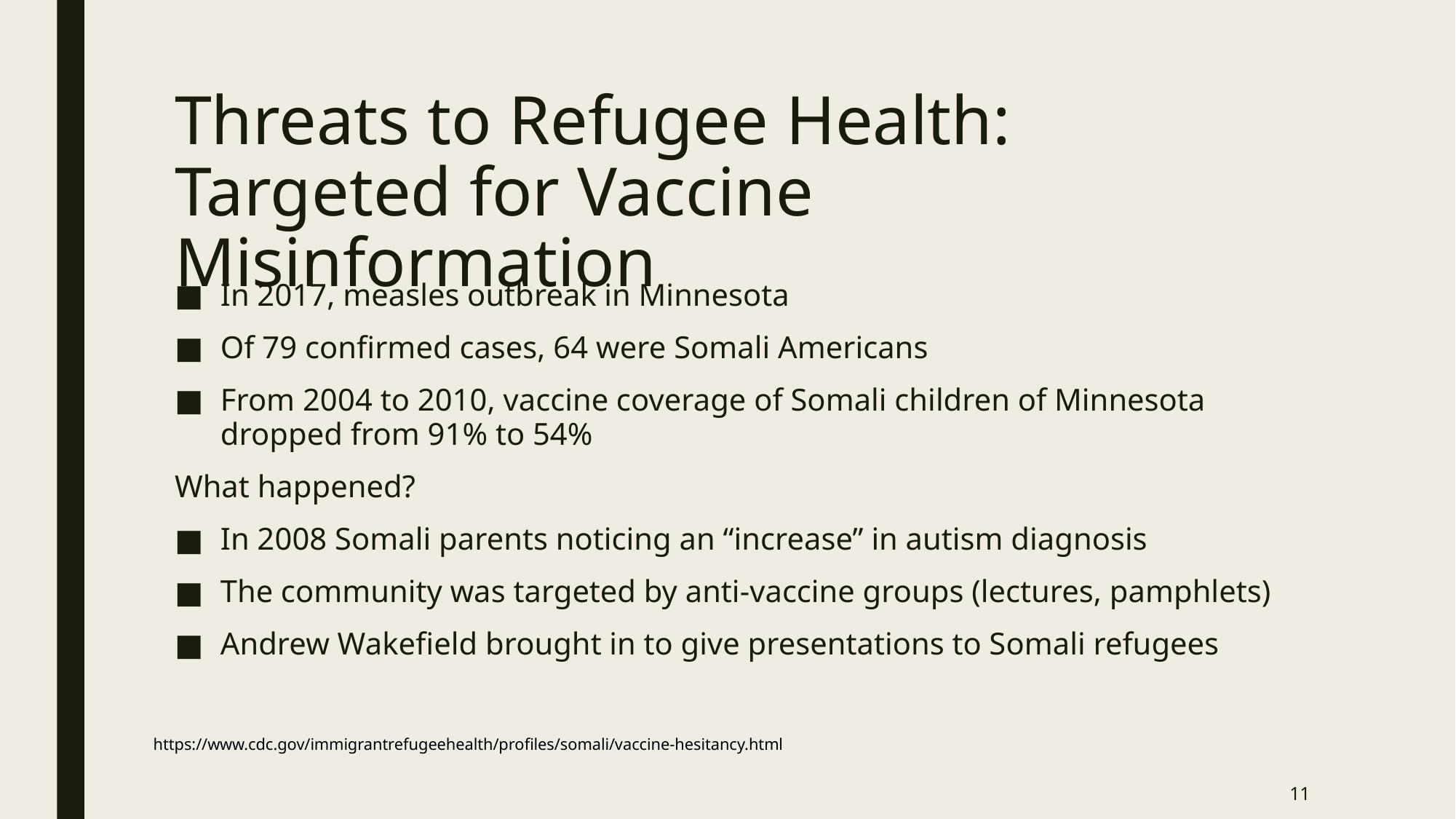

# Threats to Refugee Health: Targeted for Vaccine Misinformation
In 2017, measles outbreak in Minnesota
Of 79 confirmed cases, 64 were Somali Americans
From 2004 to 2010, vaccine coverage of Somali children of Minnesota dropped from 91% to 54%
What happened?
In 2008 Somali parents noticing an “increase” in autism diagnosis
The community was targeted by anti-vaccine groups (lectures, pamphlets)
Andrew Wakefield brought in to give presentations to Somali refugees
https://www.cdc.gov/immigrantrefugeehealth/profiles/somali/vaccine-hesitancy.html
11

## Slide 12
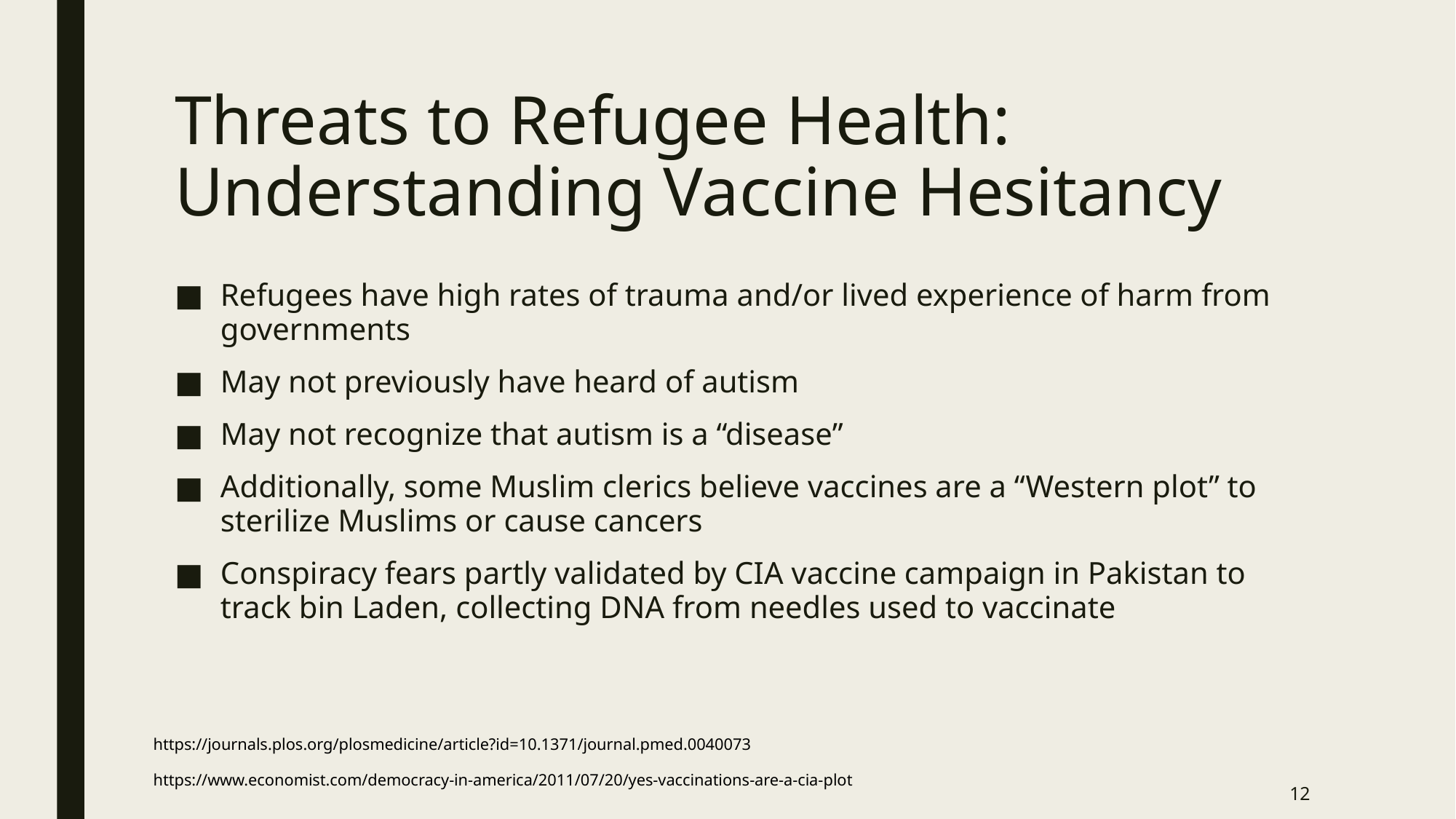

# Threats to Refugee Health: Understanding Vaccine Hesitancy
Refugees have high rates of trauma and/or lived experience of harm from governments
May not previously have heard of autism
May not recognize that autism is a “disease”
Additionally, some Muslim clerics believe vaccines are a “Western plot” to sterilize Muslims or cause cancers
Conspiracy fears partly validated by CIA vaccine campaign in Pakistan to track bin Laden, collecting DNA from needles used to vaccinate
https://journals.plos.org/plosmedicine/article?id=10.1371/journal.pmed.0040073
https://www.economist.com/democracy-in-america/2011/07/20/yes-vaccinations-are-a-cia-plot
12

## Slide 13
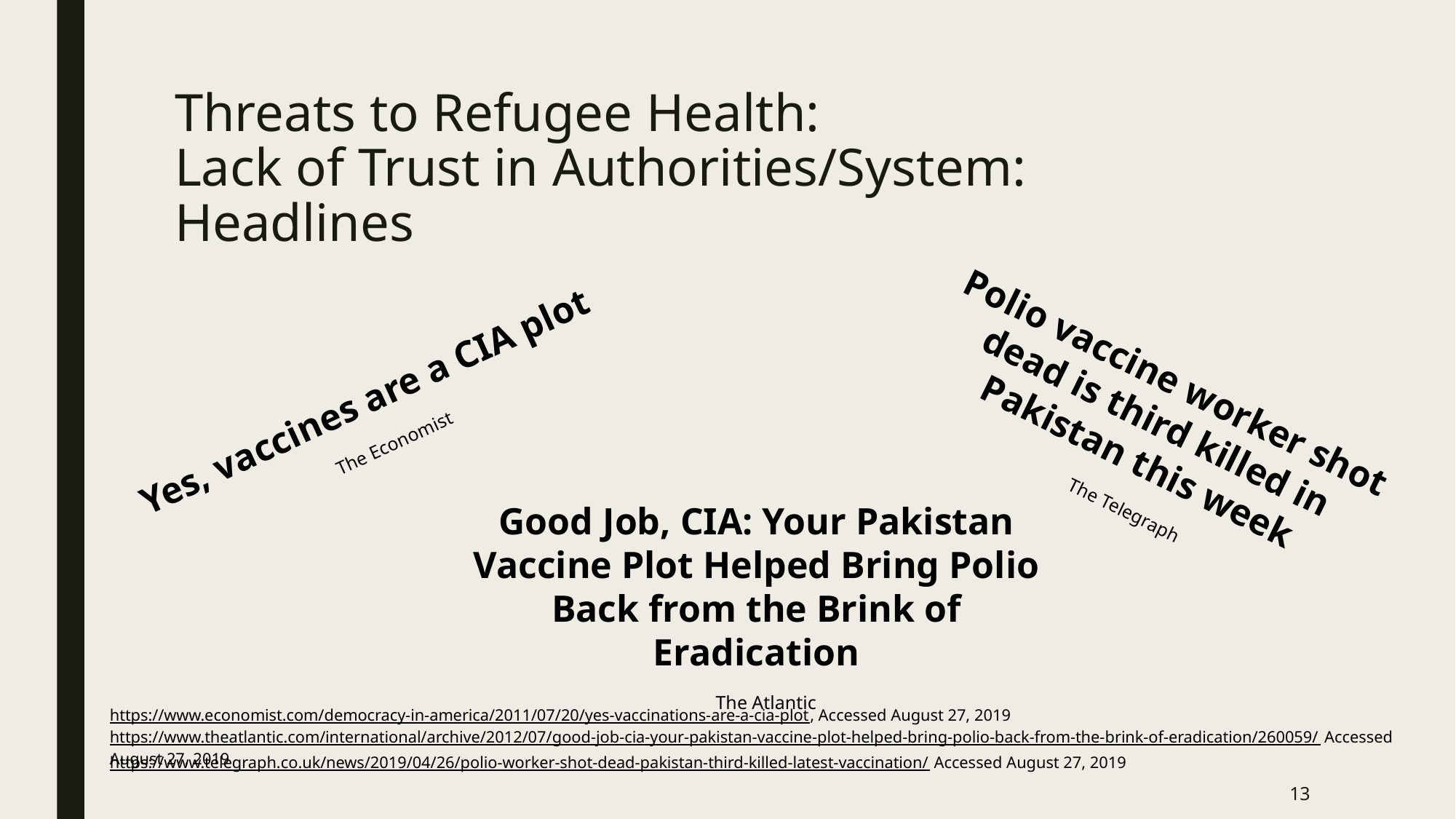

# Threats to Refugee Health: Lack of Trust in Authorities/System: Headlines
Polio vaccine worker shot dead is third killed in Pakistan this week
 The Telegraph
Yes, vaccines are a CIA plot
 The Economist
Good Job, CIA: Your Pakistan Vaccine Plot Helped Bring Polio Back from the Brink of Eradication
 The Atlantic
https://www.economist.com/democracy-in-america/2011/07/20/yes-vaccinations-are-a-cia-plot, Accessed August 27, 2019
https://www.theatlantic.com/international/archive/2012/07/good-job-cia-your-pakistan-vaccine-plot-helped-bring-polio-back-from-the-brink-of-eradication/260059/ Accessed August 27, 2019
https://www.telegraph.co.uk/news/2019/04/26/polio-worker-shot-dead-pakistan-third-killed-latest-vaccination/ Accessed August 27, 2019
13

## Slide 14
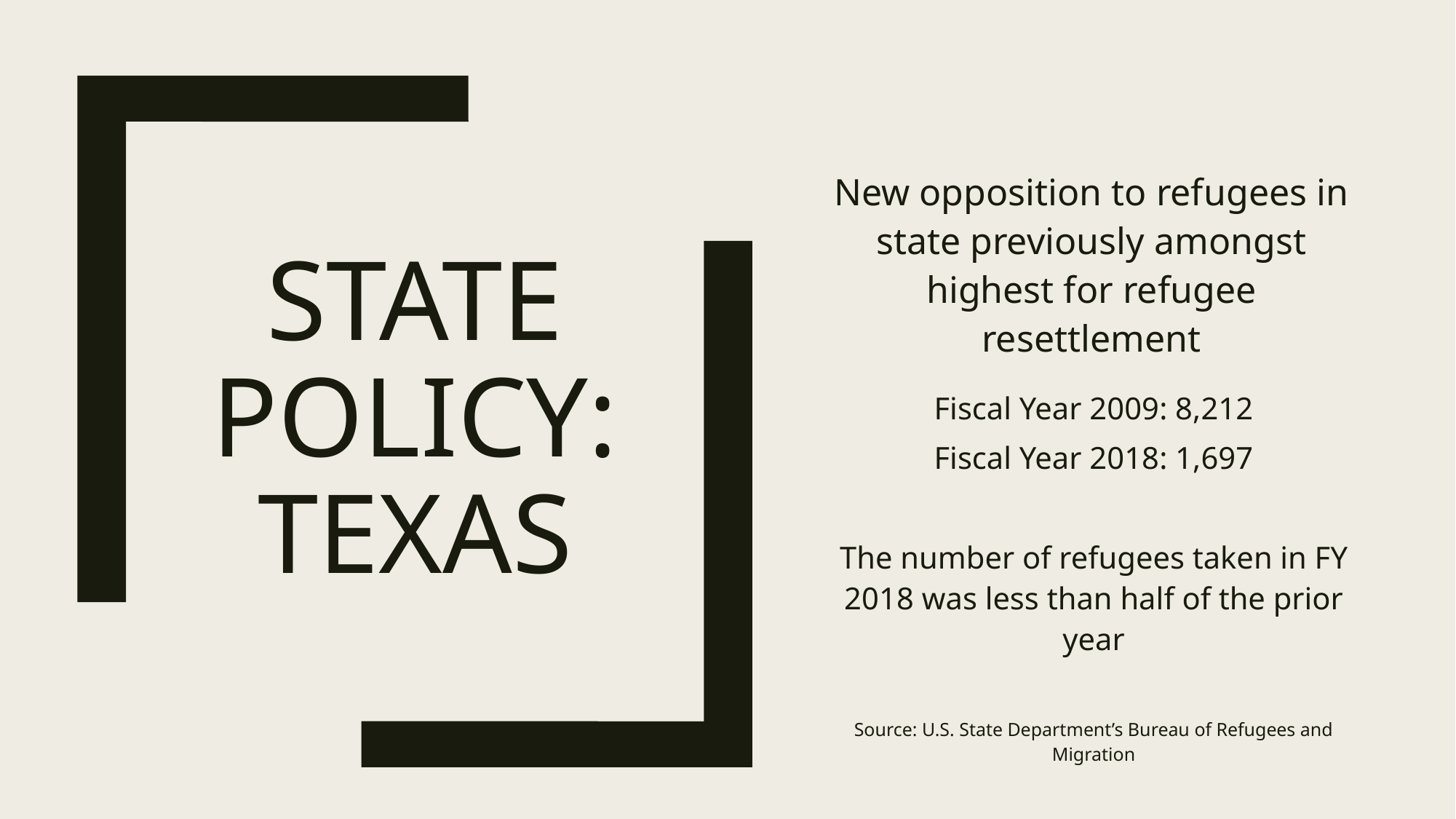

# State Policy: Texas
New opposition to refugees in state previously amongst highest for refugee resettlement
Fiscal Year 2009: 8,212
Fiscal Year 2018: 1,697
The number of refugees taken in FY 2018 was less than half of the prior year
Source: U.S. State Department’s Bureau of Refugees and Migration
14

## Slide 15
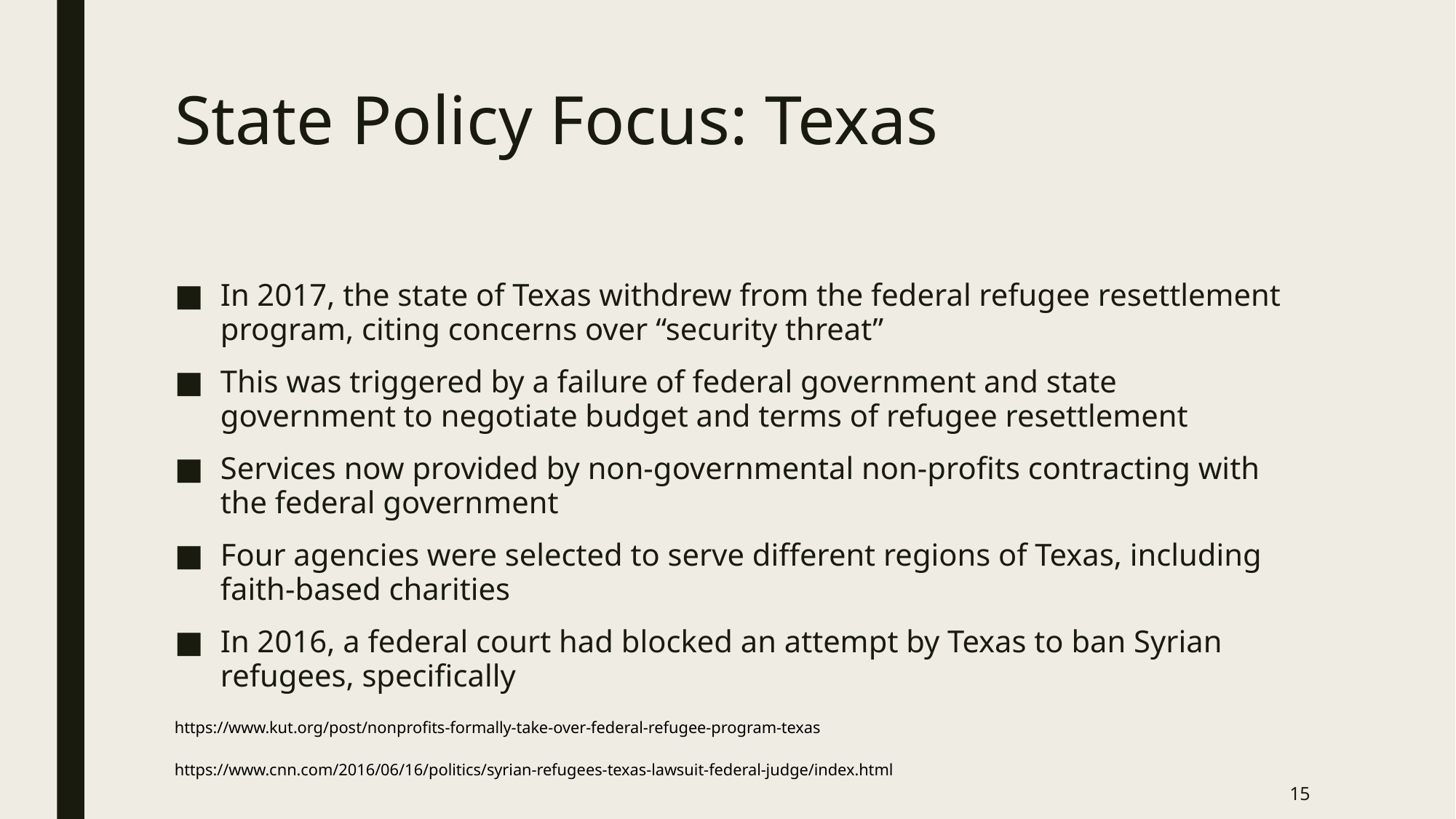

# State Policy Focus: Texas
In 2017, the state of Texas withdrew from the federal refugee resettlement program, citing concerns over “security threat”
This was triggered by a failure of federal government and state government to negotiate budget and terms of refugee resettlement
Services now provided by non-governmental non-profits contracting with the federal government
Four agencies were selected to serve different regions of Texas, including faith-based charities
In 2016, a federal court had blocked an attempt by Texas to ban Syrian refugees, specifically
https://www.kut.org/post/nonprofits-formally-take-over-federal-refugee-program-texas
https://www.cnn.com/2016/06/16/politics/syrian-refugees-texas-lawsuit-federal-judge/index.html
15

## Slide 16
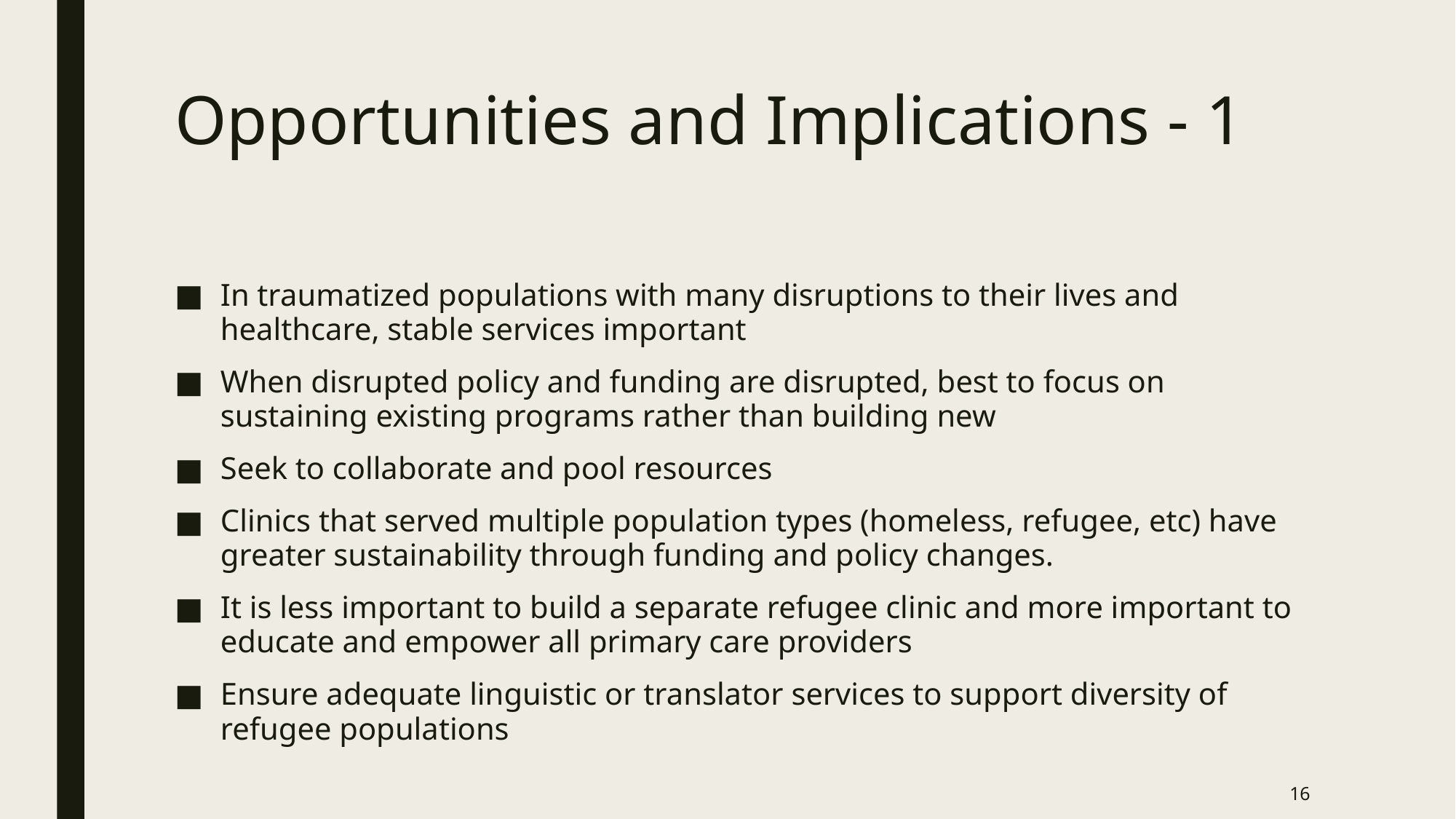

# Opportunities and Implications - 1
In traumatized populations with many disruptions to their lives and healthcare, stable services important
When disrupted policy and funding are disrupted, best to focus on sustaining existing programs rather than building new
Seek to collaborate and pool resources
Clinics that served multiple population types (homeless, refugee, etc) have greater sustainability through funding and policy changes.
It is less important to build a separate refugee clinic and more important to educate and empower all primary care providers
Ensure adequate linguistic or translator services to support diversity of refugee populations
16

## Slide 17
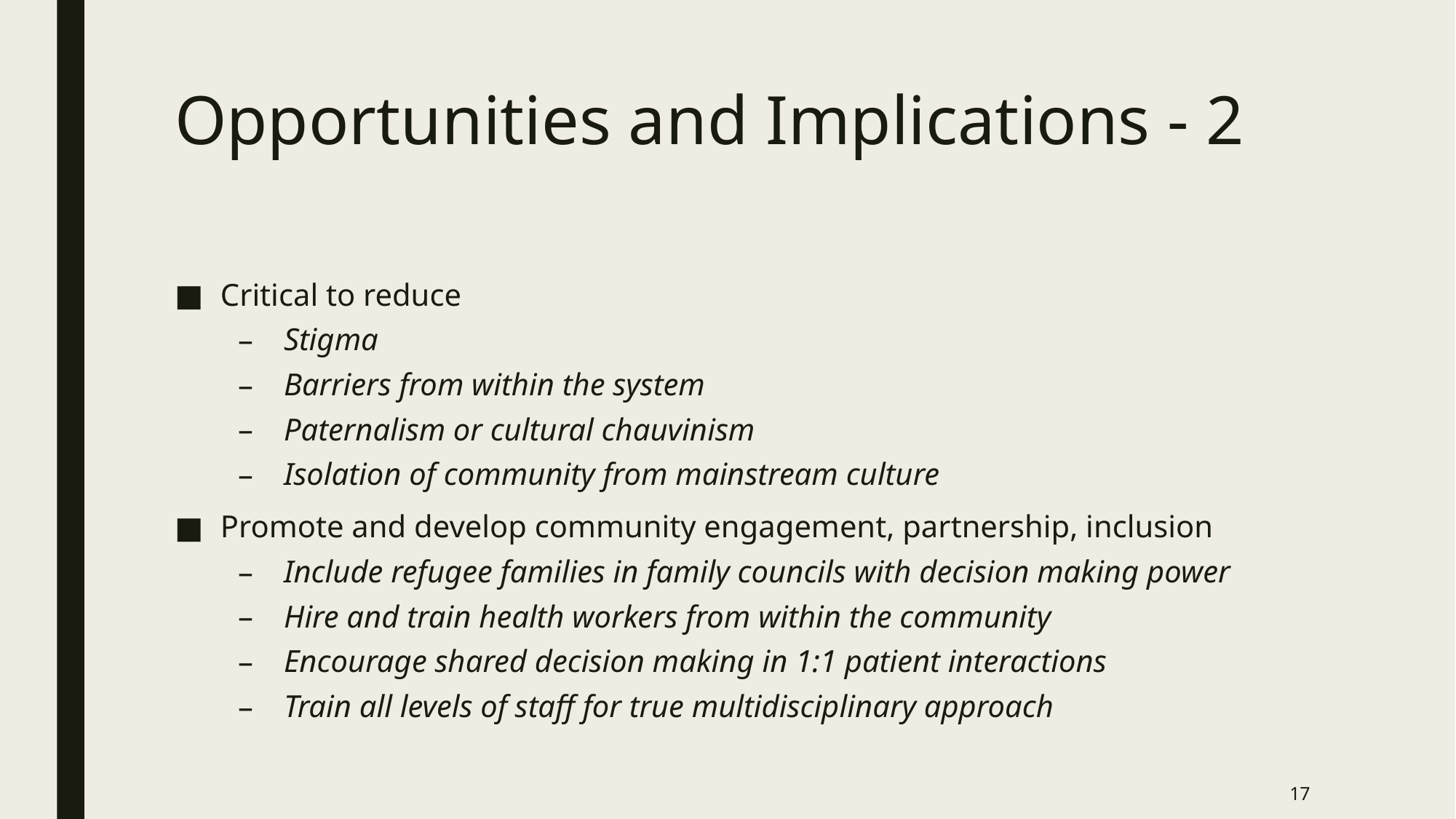

# Opportunities and Implications - 2
Critical to reduce
Stigma
Barriers from within the system
Paternalism or cultural chauvinism
Isolation of community from mainstream culture
Promote and develop community engagement, partnership, inclusion
Include refugee families in family councils with decision making power
Hire and train health workers from within the community
Encourage shared decision making in 1:1 patient interactions
Train all levels of staff for true multidisciplinary approach
17

## Slide 18
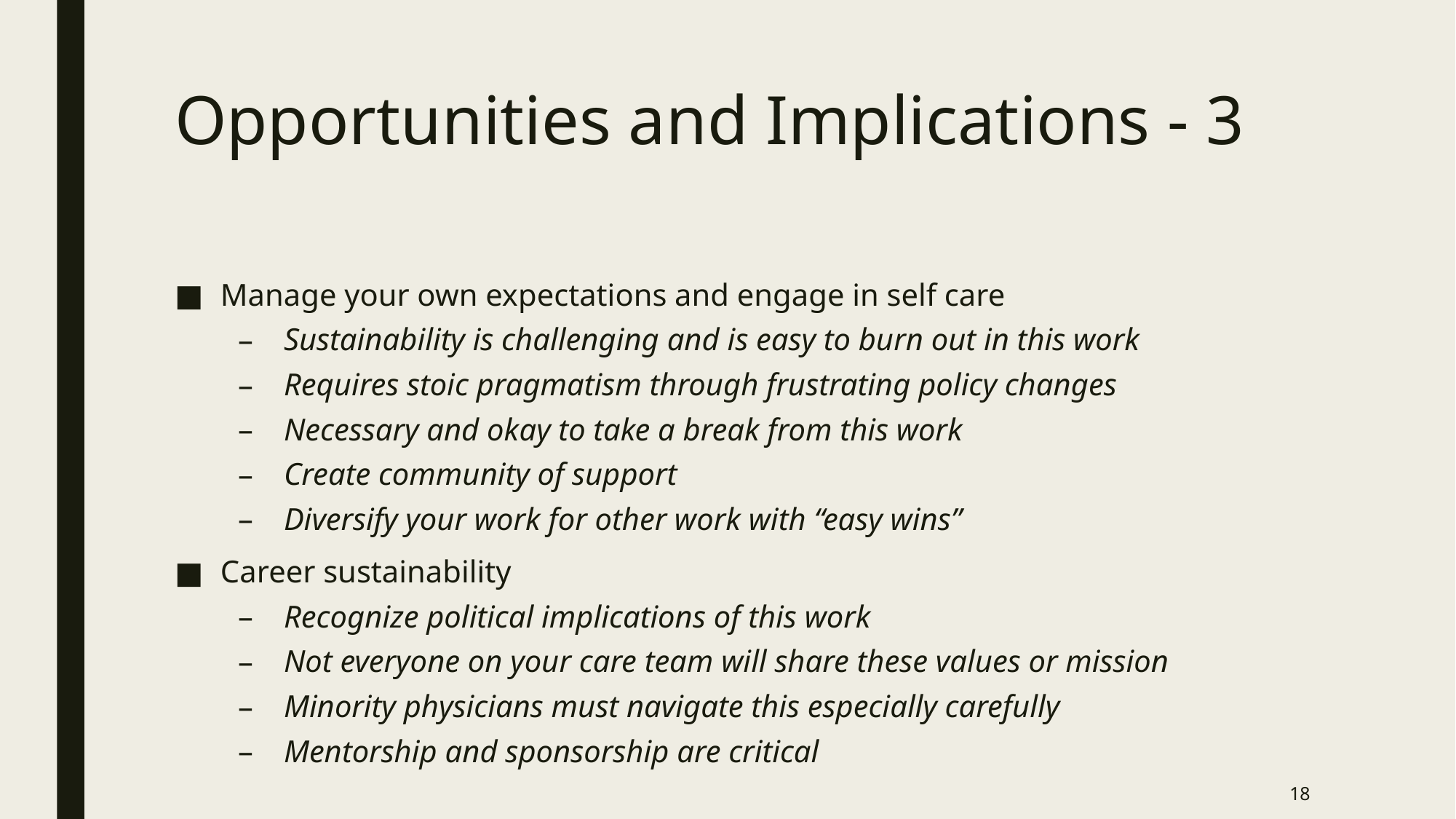

# Opportunities and Implications - 3
Manage your own expectations and engage in self care
Sustainability is challenging and is easy to burn out in this work
Requires stoic pragmatism through frustrating policy changes
Necessary and okay to take a break from this work
Create community of support
Diversify your work for other work with “easy wins”
Career sustainability
Recognize political implications of this work
Not everyone on your care team will share these values or mission
Minority physicians must navigate this especially carefully
Mentorship and sponsorship are critical
18

## Slide 19
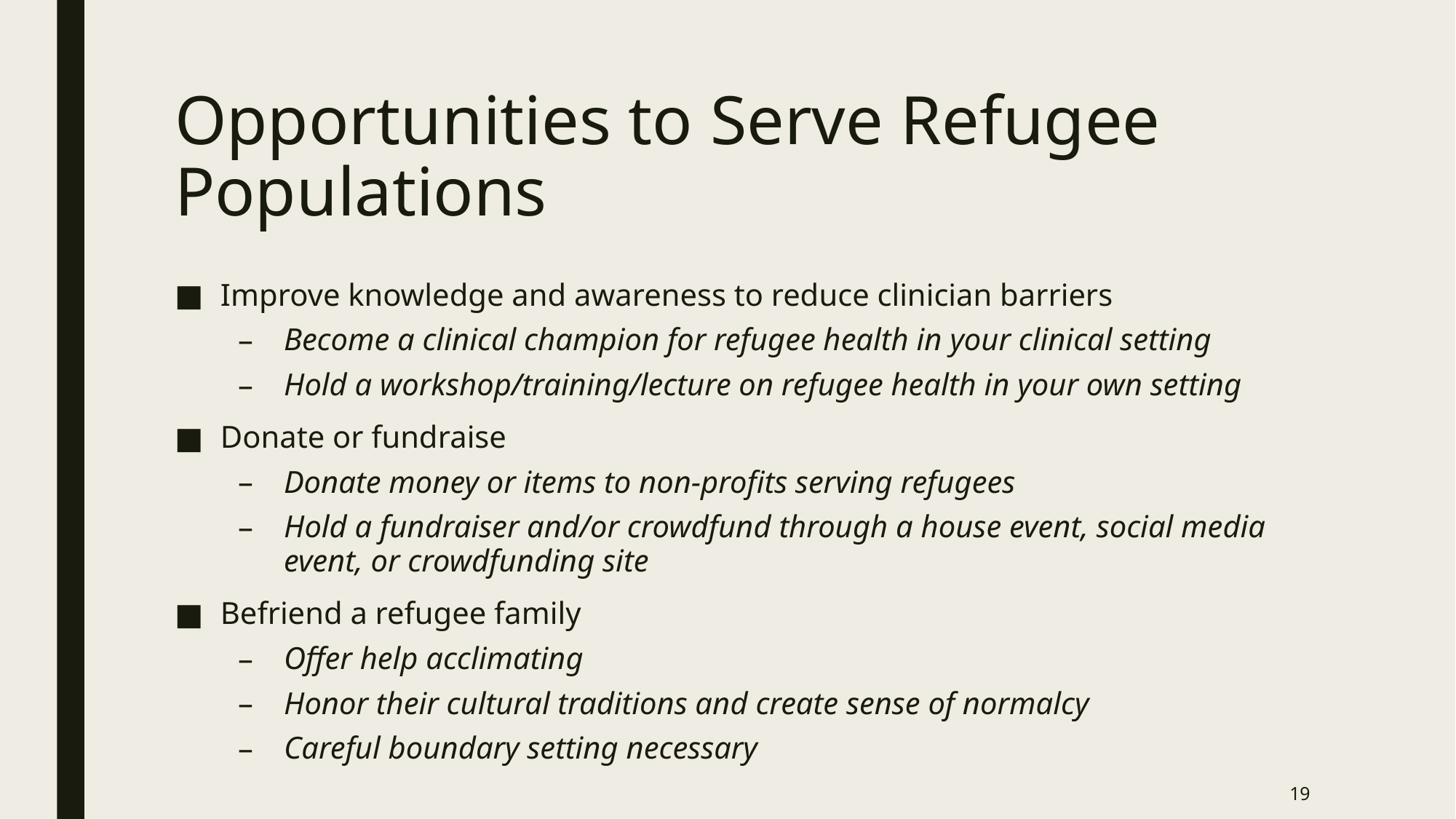

# Opportunities to Serve Refugee Populations
Improve knowledge and awareness to reduce clinician barriers
Become a clinical champion for refugee health in your clinical setting
Hold a workshop/training/lecture on refugee health in your own setting
Donate or fundraise
Donate money or items to non-profits serving refugees
Hold a fundraiser and/or crowdfund through a house event, social media event, or crowdfunding site
Befriend a refugee family
Offer help acclimating
Honor their cultural traditions and create sense of normalcy
Careful boundary setting necessary
19

## Slide 20
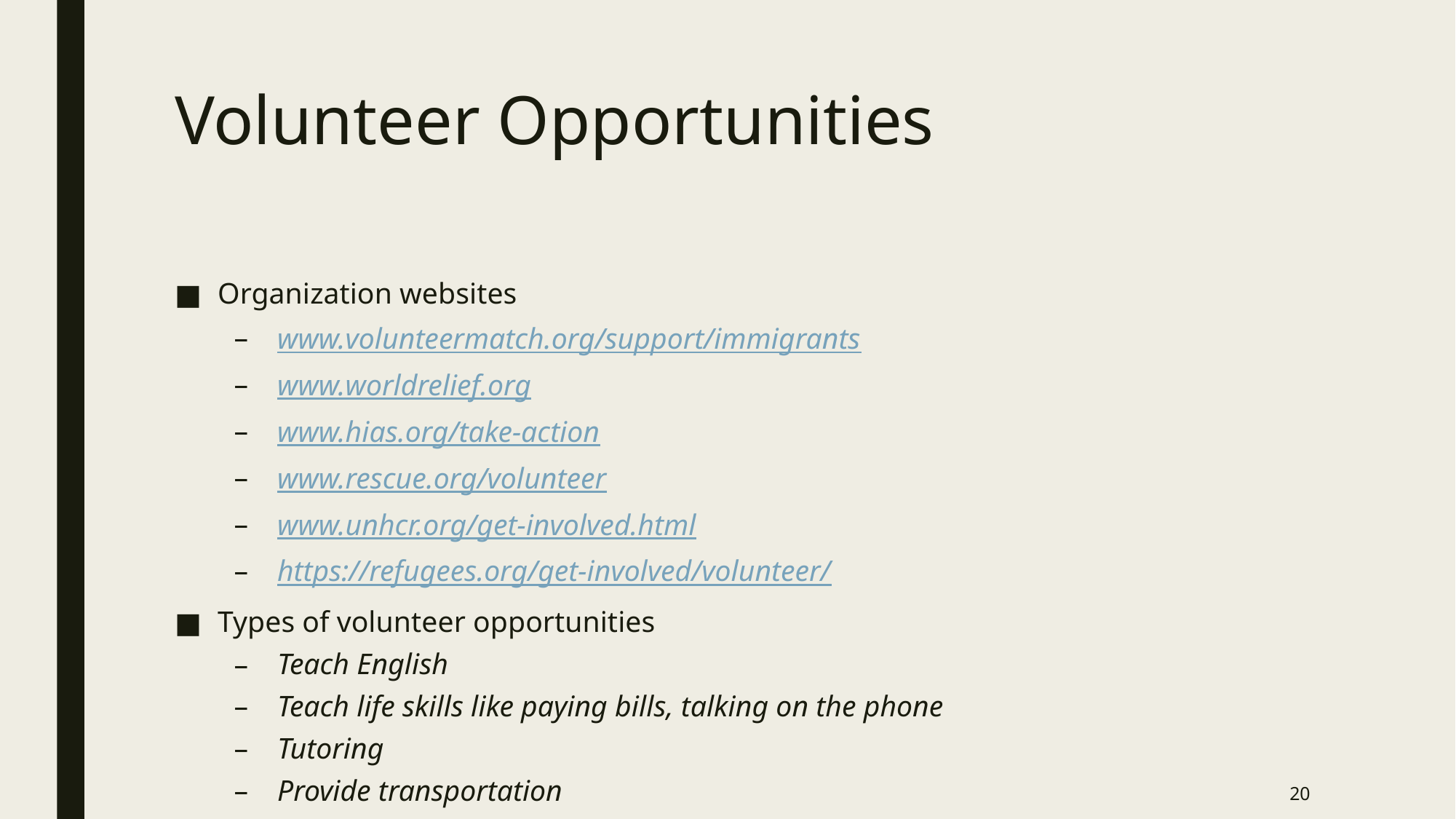

# Volunteer Opportunities
Organization websites
www.volunteermatch.org/support/immigrants
www.worldrelief.org
www.hias.org/take-action
www.rescue.org/volunteer
www.unhcr.org/get-involved.html
https://refugees.org/get-involved/volunteer/
Types of volunteer opportunities
Teach English
Teach life skills like paying bills, talking on the phone
Tutoring
Provide transportation
20

## Slide 21
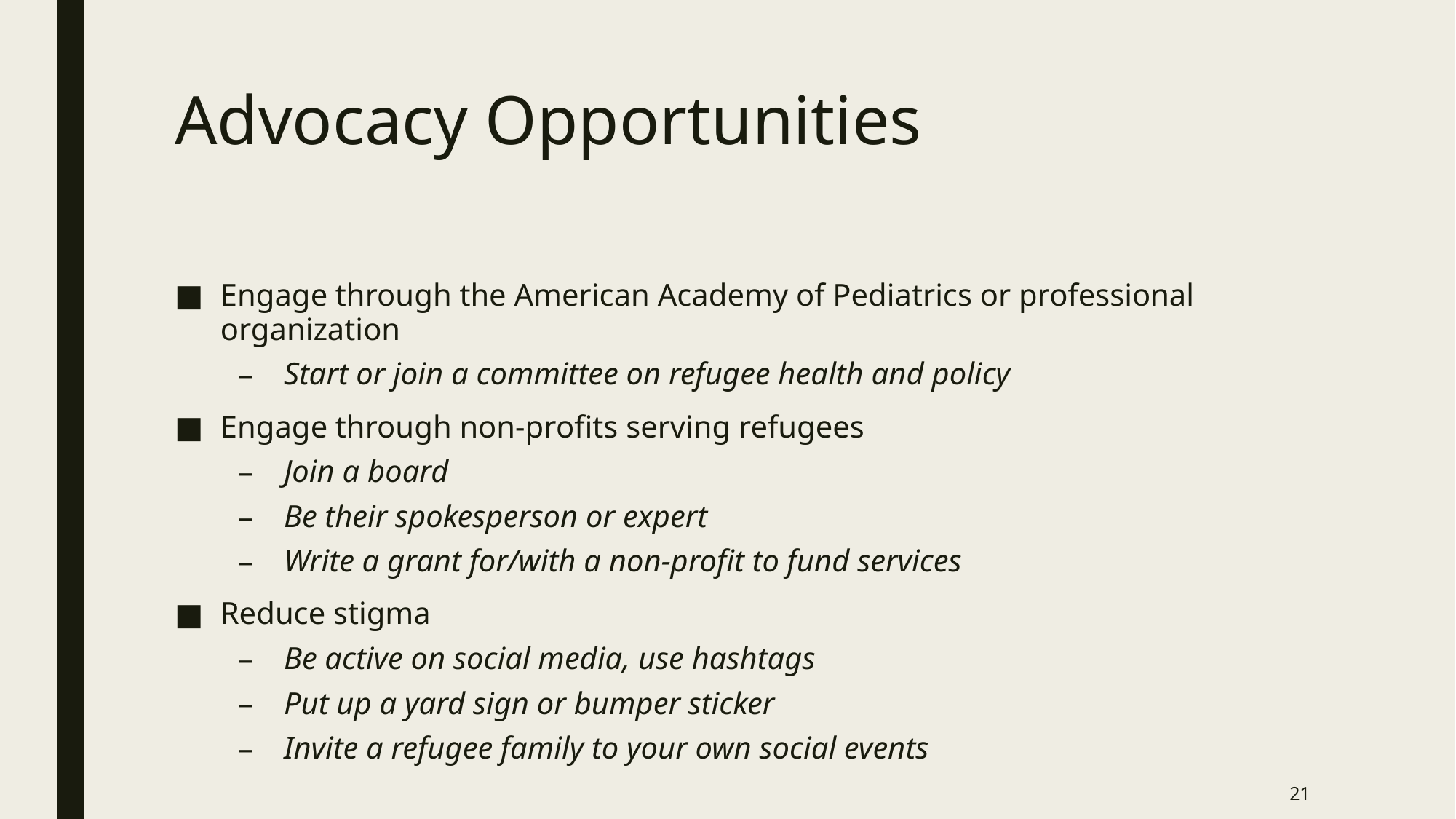

# Advocacy Opportunities
Engage through the American Academy of Pediatrics or professional organization
Start or join a committee on refugee health and policy
Engage through non-profits serving refugees
Join a board
Be their spokesperson or expert
Write a grant for/with a non-profit to fund services
Reduce stigma
Be active on social media, use hashtags
Put up a yard sign or bumper sticker
Invite a refugee family to your own social events
21

## Slide 22
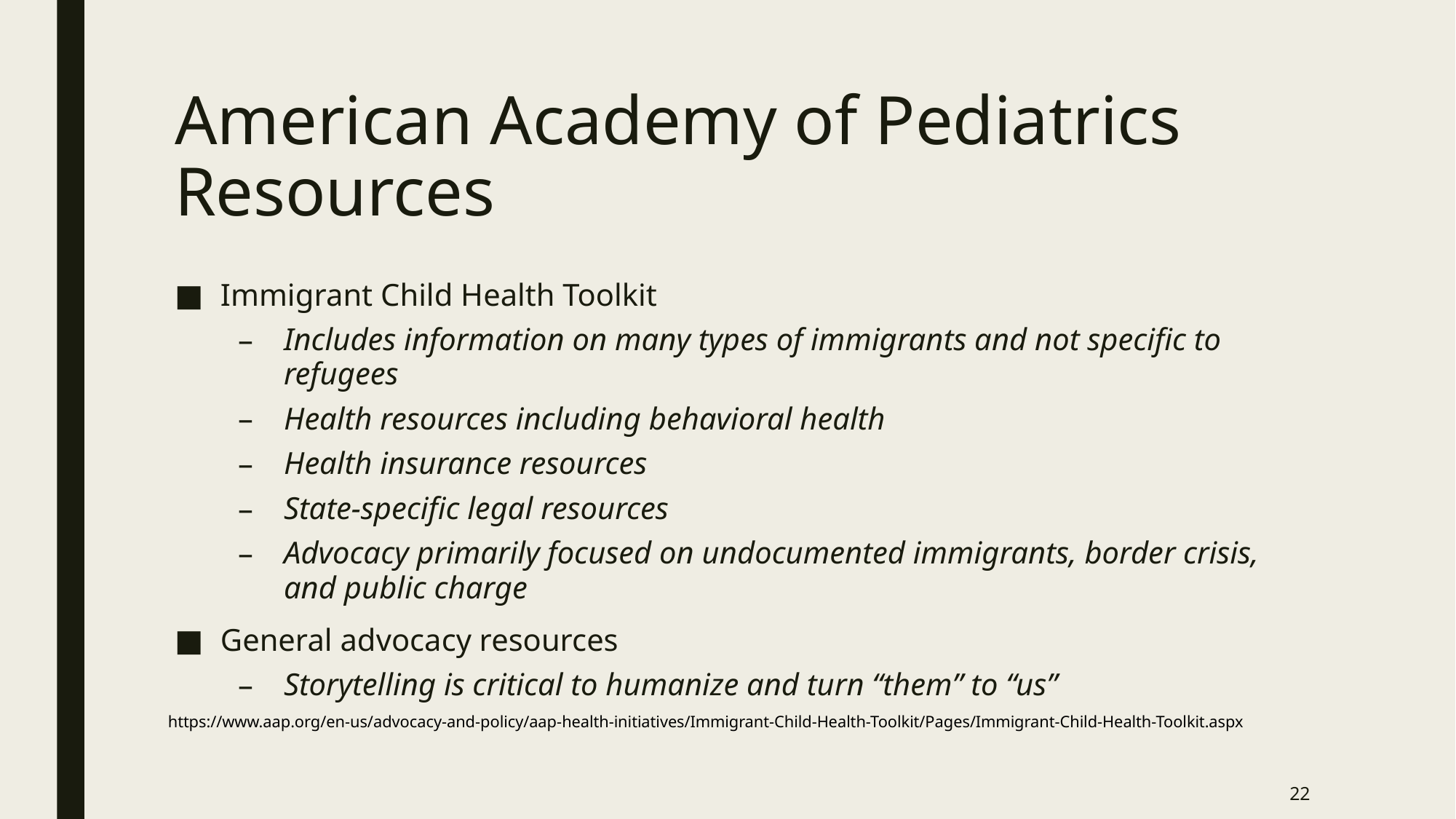

# American Academy of Pediatrics Resources
Immigrant Child Health Toolkit
Includes information on many types of immigrants and not specific to refugees
Health resources including behavioral health
Health insurance resources
State-specific legal resources
Advocacy primarily focused on undocumented immigrants, border crisis, and public charge
General advocacy resources
Storytelling is critical to humanize and turn “them” to “us”
https://www.aap.org/en-us/advocacy-and-policy/aap-health-initiatives/Immigrant-Child-Health-Toolkit/Pages/Immigrant-Child-Health-Toolkit.aspx
22

## Slide 23
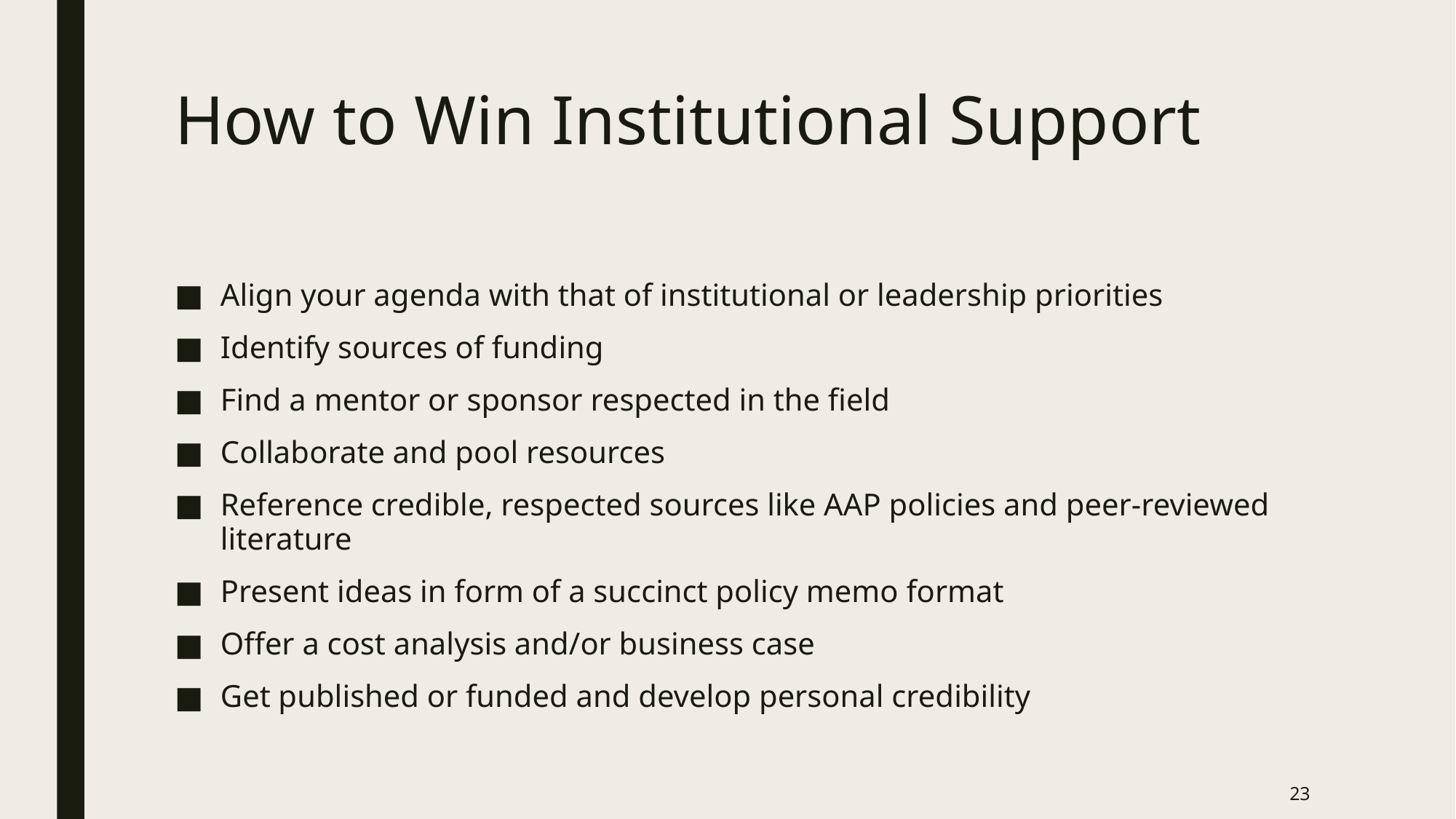

# How to Win Institutional Support
Align your agenda with that of institutional or leadership priorities
Identify sources of funding
Find a mentor or sponsor respected in the field
Collaborate and pool resources
Reference credible, respected sources like AAP policies and peer-reviewed literature
Present ideas in form of a succinct policy memo format
Offer a cost analysis and/or business case
Get published or funded and develop personal credibility
23

## Slide 24
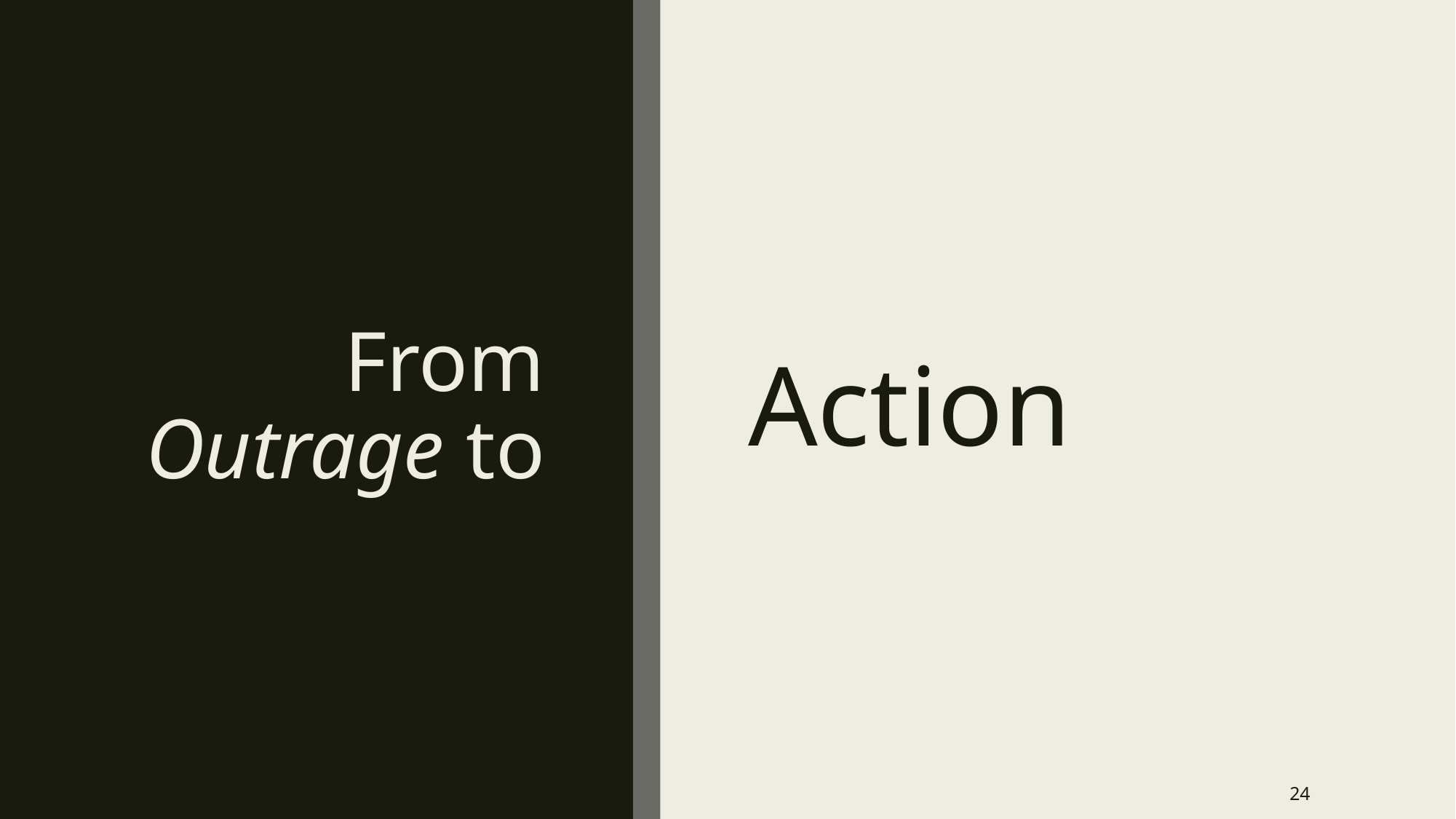

# From Outrage to
Action
24

## Slide 25
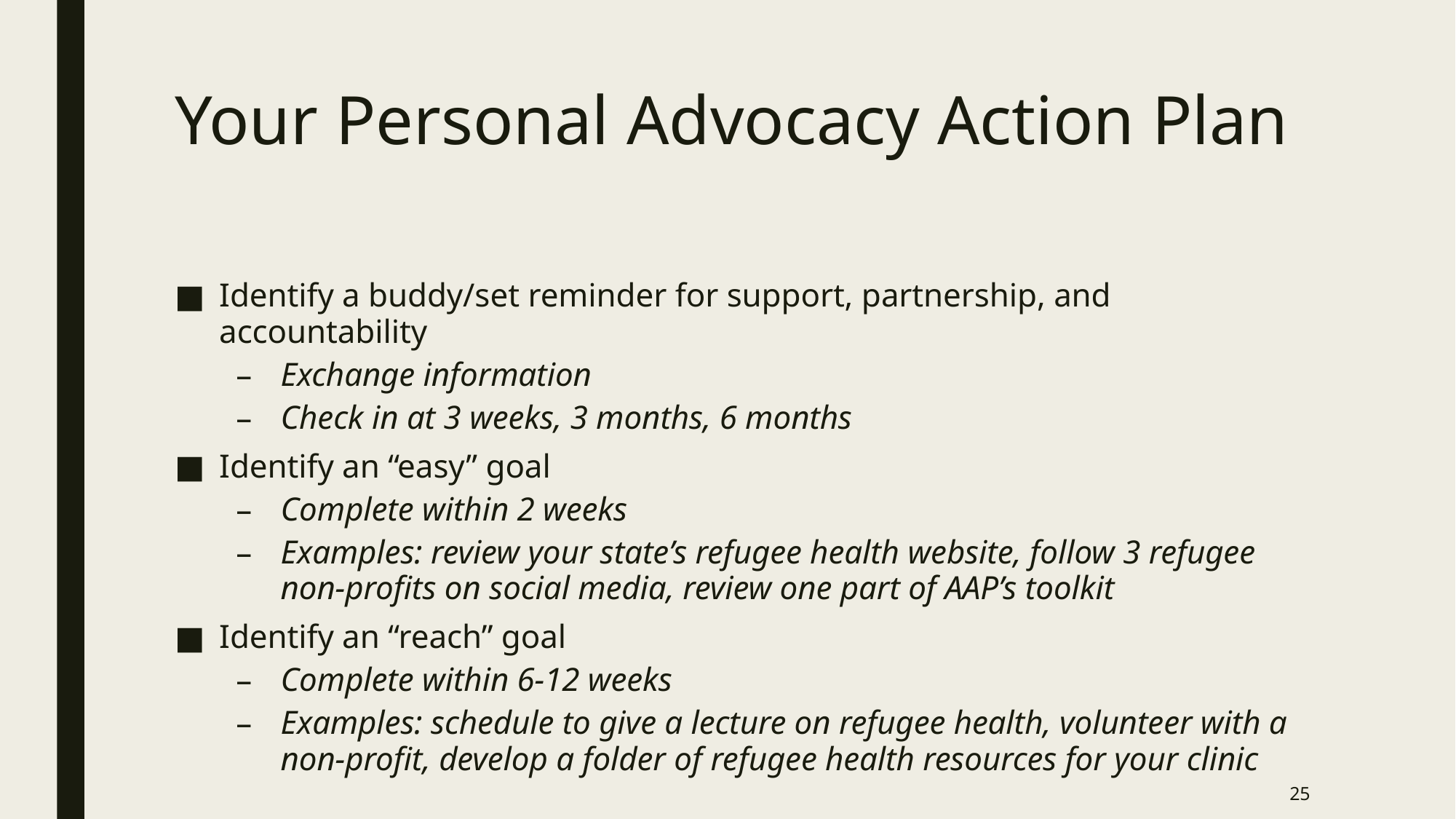

# Your Personal Advocacy Action Plan
Identify a buddy/set reminder for support, partnership, and accountability
Exchange information
Check in at 3 weeks, 3 months, 6 months
Identify an “easy” goal
Complete within 2 weeks
Examples: review your state’s refugee health website, follow 3 refugee non-profits on social media, review one part of AAP’s toolkit
Identify an “reach” goal
Complete within 6-12 weeks
Examples: schedule to give a lecture on refugee health, volunteer with a non-profit, develop a folder of refugee health resources for your clinic
25

## Slide 26
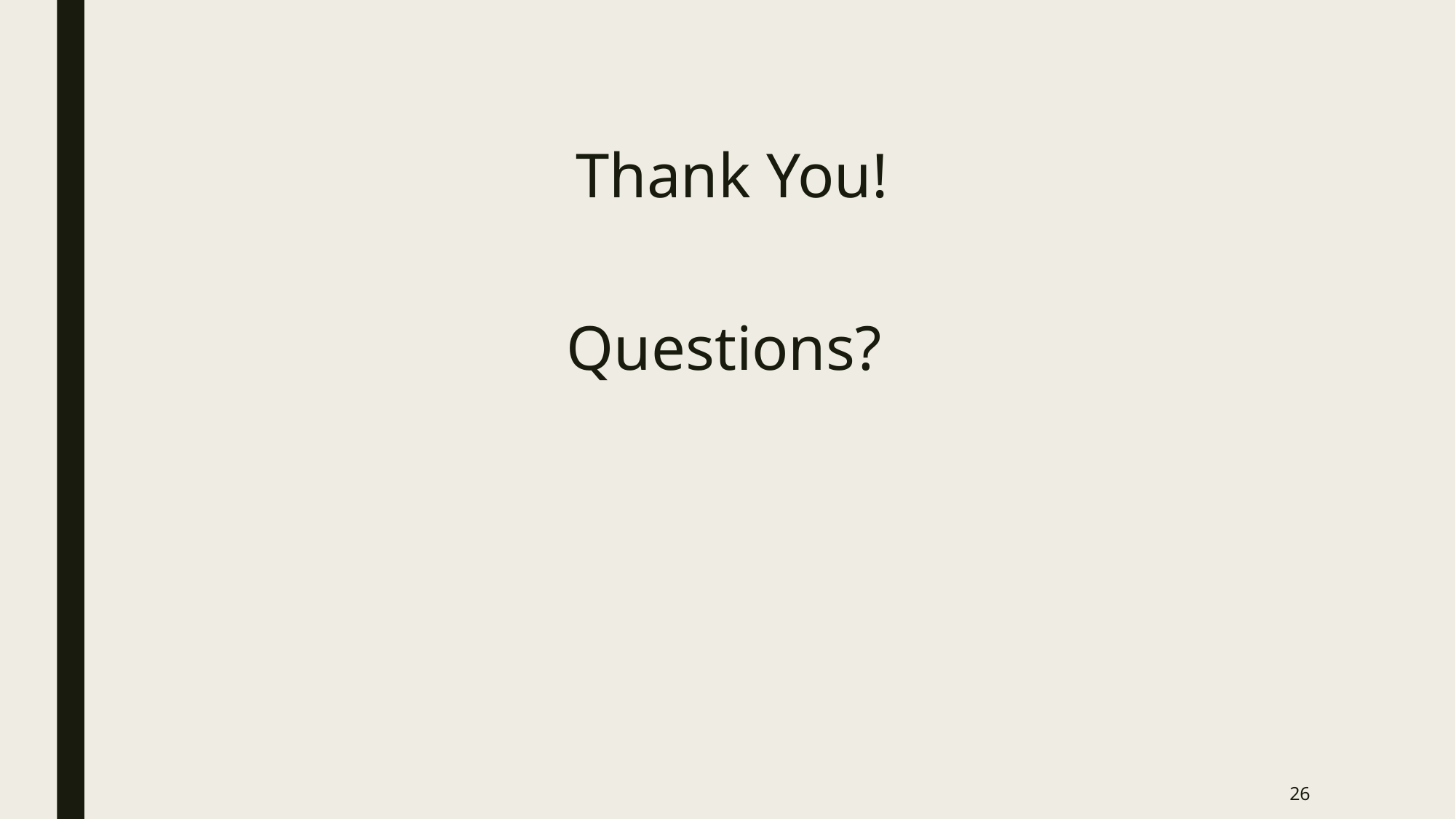

Thank You!
Questions?
26
