# Supplementary material for: Pediatric Refugee Health Care Delivery in the Community Setting: An Educational Workshop for Multidisciplinary Family-Centered Care During Resettlement
Source: MedEdPORTAL. 2020 Nov 3;16:10988. doi: 10.15766/mep_2374-8265.10988 (PMC7666829; doi:10.15766/mep_2374-8265.10988)
Supplement: Supplementary file 1 — Agenda.docxPresentation 1 Intro to Refugees.pptxPresentation 2 Health Screening.pptxCases.docxPresentation 3 Trauma-Informed Care.pptxPresentation 4 Refugee Health Advocacy.pptxRefugee Workshop Evaluation.docx [file mep_2374-8265.10988-s001.zip › G. Refugee Workshop Evaluation.docx]

**Delivering Refugee Healthcare in a Community Setting**

**Pre-workshop Questionnaire**

This pre-workshop questionnaire is anonymous.

1. How would you evaluate your level of **comfort** with taking care of refugee patients overall?

Not very comfortable Very comfortable

1 2 3 4 5

2. How would you evaluate your level of **understanding** of pre-arrival experiences of refugees?

Very poor Excellent

1 2 3 4 5

2. How would you evaluate your level of **understanding** of the screenings required of refugee patients?

Very poor Excellent

1 2 3 4 5

4. How would you evaluate your level of **comfort** with asking about the social and behavioral health history of refugee patients?

Not very comfortable Very comfortable

1 2 3 4 5

**Delivering Refugee Healthcare in a Community Setting**

**Post-workshop Questionnaire**

This post-workshop questionnaire is anonymous.

1. How would you evaluate your level of **comfort** with taking care of refugee patients overall?

Not very comfortable Very comfortable

1 2 3 4 5

2. How would you evaluate your level of **understanding** of pre-arrival experiences of refugees?

Very poor Excellent

1 2 3 4 5

2. How would you evaluate your level of **understanding** of the screenings required of refugee patients?

Very poor Excellent

1 2 3 4 5

4. How would you evaluate your level of **comfort** with asking about the social and behavioral health history of refugee patients?

Not very comfortable Very comfortable

1 2 3 4 5

5. Please provide any feedback about the workshop (e.g. what worked well, what could be improved, etc.).

Thank you for your time.
